# Supplementary material for: Bacteria from the Amycolatopsis genus associated with a toxic bird secrete protective secondary metabolites
Source: Nat Commun. 2024 Oct 2;15:8524. doi: 10.1038/s41467-024-52316-3 (PMC11446937; doi:10.1038/s41467-024-52316-3)
Supplement: Supplementary file 1 — Supplementary Information [file 41467_2024_52316_MOESM1_ESM.pdf]

Supplementary Methods

Content

Analysis of MiSeq sequencing data. 2
Bacterial isolation and strain identification 4
Whole genome-based taxonomic analysis. 7
Bacterial fatty acid analysis 8
Assays for lipolytic activity. 11
Assays for keratinolytic activity. 12
General Analytical Methods. 13
Analysis of bacterial co-cultivation studies. 14
Analysis of fungal co-cultures. 17
MALDI-IMS of co-cultivation experiment. 22
Analysis of metabolite production on minimal media vs. complex media. 27
Analysis of metabolite production on PDA at different time points 28
Isolation and characterization of metabolites 29
Determination of the absolute configuration of the amino acids in pachycephalamide A and demiguisin 72
Detection of peptides in bird feathers. 83
Phylogenetic analysis of biosynthetic enzymes 85
Generation of *Amycolatopsis* sp. PS_44_ISF1 knockout mutants. 92
Droplet-collapsing test of lipopetides. 100
Disc-diffusion antimicrobial activity assays against ecologically relevant indicator strains. 100
Disc-diffusion antimicrobial activity assays against clinically relevant indicator strains. 102
Protease inhibition assays. 103

Analysis of MiSeq sequencing data.

For microbiome analysis, we collected uropygial glands (UGs) from thirteen *P. schlegelii* specimens obtained from three locations in Papua New Guinea under the **research permit 99902341112** in the years 2018 and 2019. Miseq amplicon sequencing yielded 369,799 bacterial sequences (41,088 ± 13,495) from feather samples (n = 9) and 349,301 (29,108 ± 29,592) sequences from UG samples (n = 12) (Supplementary File 1).
Overall, bacterial sequences were assigned to 1,698 amplicon sequence variants (ASVs) with a majority of AVS (1,281) found in feather microbiomes, of which only 94 ASVs were shared between feather and UG microbiomes. Both chao1 richness estimate (lm: $F_{df} = 11.95_1$, p = 0.0032) and the Shannon's diversity index (lm: $F_{df} = 8.858_1$, p = 0.0089) of microbiomes were significantly higher in feathers than in UG (Figure S1A and S1B). Microbial alpha diversities did not differ between the sampling regions of the individuals ($Chao1_{lm}$: $F_{df} = 0.2312_2$, p = 0.7962; Shannon's diversity $index_{lm}$: $F_{df} = 0.0299_2$, p = 0.9705). Microbial composition differed significantly between feather and UG samples (Supplementary Figure 1A-C, PERMANOVA: $F_{df} = 3.824_1$, $R^2 = 0.1433$, p = 0.0004) and according to the sampling regions of the individuals (PERMANOVA: $F_{df} = 2.906_2$, $R^2 = 0.2178$, $p < 0.0001$). However, we didn't detect an effect of the interaction between the sample type and sample region (PERMANOVA: $F_{df} = 1.048_1$, $R^2 = 0.0393$, p = 0.3503), indicating that microbiome compositions are consistent among *P. schlegelii* individuals across different geographic regions. However, the composition of *P. schlegelii* UG microbiomes differed notably from temperate bird species.[1]

Supplementary Table 1. Collection information (research permit 99902341112). Natural History Museum ID, capture site, elevation, longitudinal and latitudinal coordinates, and capture year of *Pachycephala schlegelii* individuals used in this study.

Museum ID (Individual ID)	Collection locality (elevation of capture site is given with meters above sea level – m.a.s.l.)	Collection year	Longitude and latitude
NHMD 615979 (KHB1-12.11.18)	Yawan, Huon Peninsula, 2,400 m.a.s.l.	2018	6.0952755 S 146.91583333 E
NHMD 615980 (KHB2-12.11.18)	Yawan, Huon Peninsula, 2,400 m.a.s.l.	2018	6.0952755 S 146.91583333 E
NHMD 615981 (KHB7-13.11.18)	Yawan, Huon Peninsula, 2,400 m.a.s.l.	2018	6.0952755 S 146.91583333 E
NHMD 615982 (KHB19-14.11.18)	Yawan, Huon Peninsula, 2,400 m.a.s.l.	2018	6.0952755 S 146.91583333 E
NHMD 615983 (KHB11-15.11.18)	Yawan, Huon Peninsula, 2,400 m.a.s.l.	2018	6.0952755 S 146.91583333 E
NHMD 642444 (KHB9-22.7.19)	Mount Wilhelm, 2,200 m.a.s.l.	2019	5.75944444 S 145.18027778 E
NHMD 642445 (KHB7-23.7.19)	Mount Wilhelm, 2,200 m.a.s.l.	2019	5.75944444 S 145.18027778 E
NHMD 642476 (KHB7-28.7.19)	Mount Wilhelm, 2,200 m.a.s.l.	2019	5.75944444 S 145.18027778 E
NHMD 307097	Macgregor Camp, Mt. Scratchley	2019	8.779683 S 147.505417 E
NHMD 307092	Macgregor Camp, Mt. Scratchley	2019	8.779683 S 147.505417 E
NHMD 307108	Macgregor Camp, Mt. Scratchley	2019	8.779683 S 147.505417 E
NHMD 307098	Macgregor Camp, Mt. Scratchley	2019	8.779683 S 147.505417 E
NHMD 307064	Macgregor Camp, Mt. Scratchley	2019	8.779683 S 147.505417 E

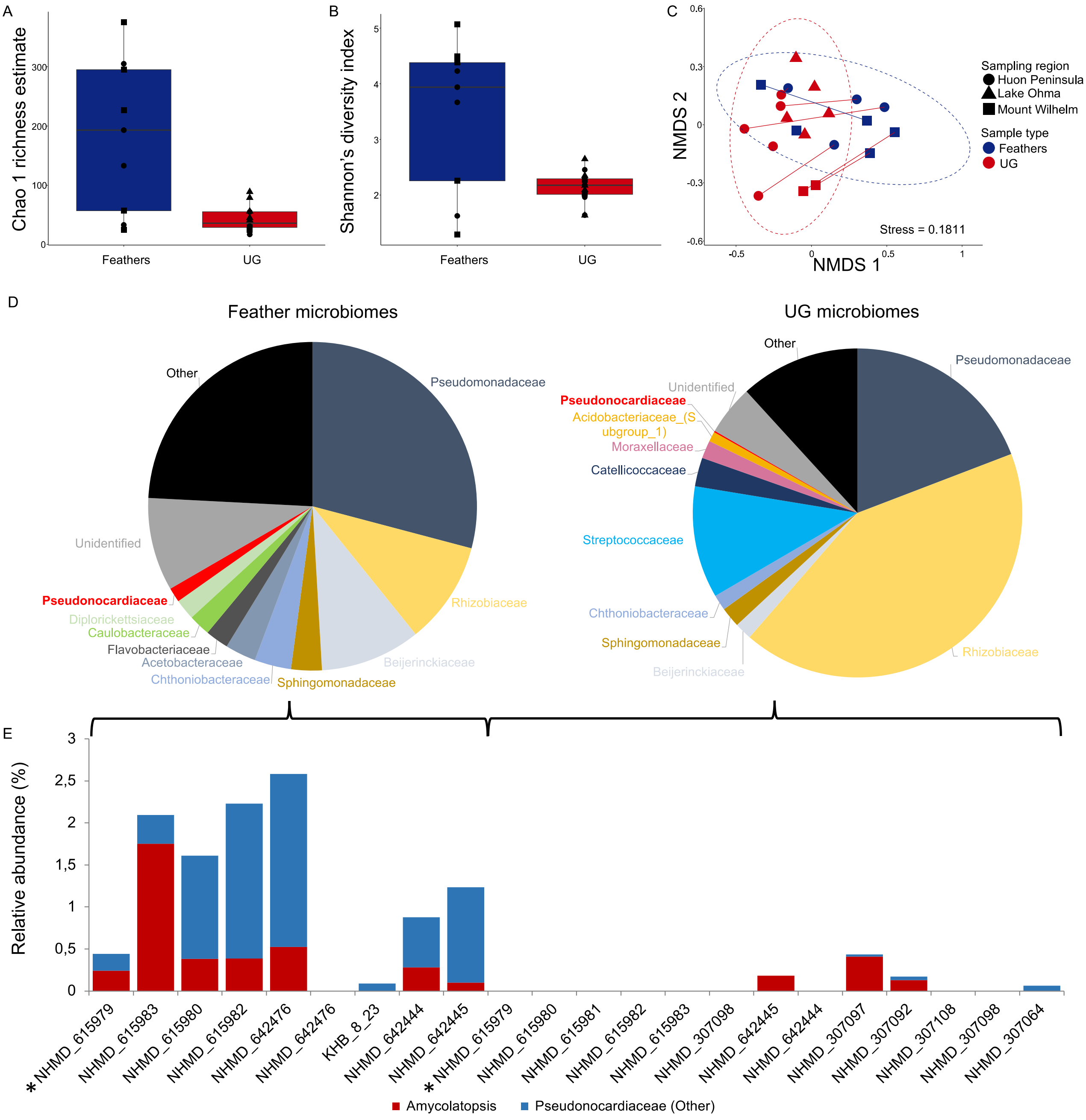

Supplementary Figure 1. Microbiome analysis of feather and uropygial glands (UG) of *Pachycephala schlegelii*. **A** and **B.** Bacterial richness and Shannon's diversity index of feather and UG microbiomes. **C.** NMDS plots depicting the feather and UG microbiome compositional difference. Ellipses indicate the 95% confidence intervals. **D**. Averaged relative abundance of the nine most abundant bacterial families and family Pseudonocardiaceae found across feather and UG samples. **E**. Relative abundance of ASVs belonging to the genus *Amycolatopsis* in each microbiome sample. Natural history museum IDs of each bird individual (except for KHB8-23 individual where we only collected feather samples and released the bird) are given in the x axis and the individual where the *Amycolatopsis* strain was isolated indicated with an asterisk. We detected 16 ASVs belonging to the genus *Amycolatopsis*, where seven out of nine feather samples and three out of 13 UG samples harbored *Amycolatopsis*. While, we were unable to detect *Amycolatopsis* sequences in the UG of NHMD 615979 (the individual we isolated the *Amycolatopsis* strain from), two AVS belonging to *Amycolatopsis* were detected in the feather sample of the individual.
Source data are provided as a Source Data file.

Bacterial isolation and strain identification

Bacterial strains were isolated from the uropygial gland (UG) secretions of five *Pachycephala schlegelii* (Regent Whistler) in Papua New Guinea in 2018 (Supplementary Table 1: **NHMD 615979,** NHMD 615980, NHMD 615981, NHMD 615982 and NHMD 615983). The outside of the UG were sterilized with 70% EtOH and massaged slowly until the secretions emerged. The secretions were collected with a sterile Copan mini FLOQ swabsTM (Brescia, Italy) and applied directly onto a Potato Dextrose Agar medium (PDA, 32 g of PDA mixed with 800 mL of water) containing 50 mg cycloheximide per liter of media. Plates were kept in room temperature under aerobic conditions. Once the colony forming units (CFUs) appeared, morphologically different colonies were isolated and reinoculated in new PDA media without cycloheximide. This process let to the isolation of 23 bacterial strains. One CFU (named PS_44_ISF1) isolated from the biosample NHMD 615979 demonstrated filamentous bacterial growth and a visible emerging inhibition zone in proximity to other bacterial isolates.

Strain identification. Strain PS_44_ISF1 was sub-cultured on MS agar, and single colonies stored in 25% glycerol at -80 °C. Bacterial DNA from the strain was acquired using Qiagen DNeasy® Blood and Tissue kit (Hilden, Germany), with a 24-hour incubation period. Initial Sanger sequencing was conducted at the Eurofins genomics (Copenhagen, Denmark), with the bacterial 16S rRNA gene primer pair 27F and 1492R to identify the bacterial isolate.[2]

Supplementary Table 2. Recipes of media used in this study. Prior to use, all media were autoclaved at 121 °C for 20 min. For *E. coli* was cultivated on Luria-Bertani (LB) medium/agar. For selection of transformants and ex-conjugants, media were supplemented with antibiotics (depending on the purpose): 50 µg/mL apramycin (Apr), 25 µg/mL chloramphenicol (Cam), 25 µg/mL kanamycin (Kan), or 25 µg/mL nalidixic acid (NA). If not stated differently, all media components were purchased at Carl Roth, Karlsruhe.

Medium	Composition (per L)	Purpose
MS broth/ agar	20 g *D(-)* mannitol (Sigma-Aldrich), 20 g soybean flour (Sigma-Aldrich), for agar: 2% agar (w/v)	Standard cultivation/ conjugation
ISP2 broth/ agar	4 g yeast extract, 10 g malt extract, 4 g dextrose, pH 7.2, for agar: 2% agar (w/v)	Standard cultivation/ metabolite production medium
CSA	10 g starch (soluble), 1 g casein hydrolysate, 0.5 g K_2HPO_4, 1.5% agar (w/v), pH 7.0	Minimal medium
Supplemented minimal medium, solid (SMMS) (modified Kieser et al.)[20]	2 g casamino acids, 3 g Tris (25 mM), 0.1 g NaH_2PO_4 (1 mM), 0.17 g K_2HPO_4 (1 mM), 9 g glucose (50 mM), 1 mL trace elements, 1% agar (w/v), pH 7.2	Minimal medium
Trace elements (for SMMS)	1 g $ZnSO_4x7H_2O$, 1 g $FeSO_4x7H_2O$, 1 g $MnCl_2x4H_2O$, 1 g $CaCl_2x6H_2O$, 1 NaCl	
MM (Kieser et al.) [20]	0.5 g L-asparagine, 0.5 g K2HPO4, 0.2 g $MgSO_4x7H_2O$, 0.01 g $FeSO_4x7H_2O$, 10 g glucose, 1% agar (w/v), pH 7.2	Minimal medium
ISP4	10 g soluble starch, 2 g $CaCO_3$, 1 g K_2HPO_4, 1 g $MgSO_4x7H_2O$, 1 g NaCl, 2 g $(NH_4)_2SO_4$, 1 ml trace elements, 2% agar (w/v), pH 7.2	Minimal media
LB broth/ agar	25 g LB (Luria/Miller), for agar: 1.5% agar (w/v)	Cultivation of *E. coli*
PDB/ PDA	26.5 g potato extract glucose broth, for PDA: 2% agar (w/v)	metabolite production medium/ transcriptome analysis/ co-cultivation assays
MH broth/ agar	21 g Mueller Hinton Broth, for agar: 1.5% agar (w/v)	antimicrobial activity assays
FB[23]	0.5 g NaCl, 0.3 g K_2HPO_4, 0.4 g KH_2PO_4, 5 g feathers, 1 mL trace element solution, pH 7.2	Keratinolytic activity screening
FMB/FMA	0.5 g NaCl, 0.3 g K_2HPO_4, 0.4 g KH_2PO_4, 5 g yeast extract, 5 g feathers, pH 7.2, for FMB: 1.8% agar	Keratinolytic activity screening
Chromogenic lipase substrate agar (Singh et al. 2006)[24]	0.01 % Phenol Red (w/v), 1% lipid substrate (v/v), 10 mM $CaCl_2$, pH 7.4, 2% agar (w/v)	Lipolytic activity screening
Tween-20 agar (Gopinath et al. 2005)	10 g peptone, 5 g NaCl, 0.1 g $CaCl_2$ x $2H_2O$, 10 mL Tween-20, pH 7.0, 2% agar	Lipolytic activity screening
Trace element solution (100 mL)	0.1 g $FeSO_4$ x $7H_2O$, 0.1 g $MnCl_2$ x 4 H_2O, 0.1 g $ZnSO_4$ x 7 H_2O, pH 7.2	Preparation of FB/ ISP5

Supplementary Table 3. Strains used in this study.

Strain	Genotype	Abbreviated name	Reference
Amycolatopsis sp. M39	wild type	M39	[55]
A. saalfeldensis DSM 44993	wild type	-	[26]
Amycolatopsis sp. PS_44_ISF1	wild type	PS_44_ISF1	this study
Bacillus thuringiensis DSM1040611	wild type		[56]
Bacillus licheniformis DSM13	wild type		
Kocuria rhizophila DSM11926	wild type		
Pseudomonas monteilii DSM1388	wild type		
Staphylococcus epidermidis DSM103867	wild type		
Aspergillus fumigatus FGSC A1160	wild type		
Aspergillus niger N402	wild type		
Candida albicans SC5314	wild type		
Amycolatopsis sp. PS_44_ISF1 *Δ817*	single cross-over mutant of PS_44_ISF1 with integrated pKJ55-*817*-ko, Apr^R	PS_44_ISF1 *Δ817*	this study
Amycolatopsis sp. PS_44_ISF1 *Δ971 (pchP3)*	single cross-over mutant of PS_44_ISF1 with integrated pKJ55-*971*-ko, Apr^R	PS_44_ISF1 *Δ971 (ΔpchP3)*	this study
Amycolatopsis sp. PS_44_ISF1 *Δ1663*	single cross-over mutant of PS_44_ISF1 with integrated pKJ55-*1663*-ko, Apr^R	PS_44_ISF1 *Δ1663*	this study
Amycolatopsis sp. PS_44_ISF1 *Δ276 (dmgP5)*	single cross-over mutant of PS_44_ISF1 with integrated pKJ55-*276*-ko, Apr^R	PS_44_ISF1 *Δ276 (ΔdmgP5)*	this study
Amycolatopsis sp. PS_44_ISF1 *Δ186*	single cross-over mutant of PS_44_ISF1 with integrated pKJ55-*971*-ko, Apr^R	PS_44_ISF1 *Δ186*	this study
Escherichia coli DH5α	general cloning strain (construction of pKJ55-ko vectors via Gibson assembly)	-	laboratory strain
E. coli ET12567/pUZ8002	nonmethylating plasmid donor strain for intergeneric conjugation, Kan^R, Cam^R	-	[57]

Whole genome-based taxonomic analysis.

The genome sequence of *Amycolatopsis* sp. PS_44_ISF1 (following called user genome) was uploaded to the Type (Strain) Genome Server (TYGS) for a whole genome-based taxonomic analysis (JOB ID: 6aef1399-4d79-451e-91b7-36a88c7160d8).[3] The TYGS analysis was subdivided into the following steps: First, selected user genomes were compared against all type strain genomes available in the TYGS database using the MASH algorithm,[4] a fast approximation of intergenomic relatedness, and the ten type strains with the smallest MASH distance were chosen. 16S rDNA sequences were extracted from the user genomes using RNAmmer[5] and subsequently BLASTed[6] against 16S rDNA of all type strains available in the TYGS database. The best 50 matching type stains (according to bitscore) were chosen and the distances to the user genome were calculated using the Genome BLAST Distance Phylogeny approach (GBDP).[7] The distances were used to determine the 10 closest type strain genomes for each user genome. For phylogenomic inference, pairwise comparisons among the set of genomes were conducted using GBDP. 100 distance replicates were calculated each. Digital DDH values and confidence intervals were calculated using the recommended settings of the GGDC 3.0 (Supplementary File 2).[8] The intergenomic distances were used to infer a balanced minimum evolution tree with branch support via FASTME 2.1.6.1[9] and visualized with PhyD3.[10] Branch support was inferred from 100 pseudo-bootstrap replicates each. *Streptomyces avermitilis* DSM 46492 was used as outgroup (Supplementary Figure 2). The type-based species clustering using a 70% dDDH radius around each of the 35 type strains was done, while subspecies clustering was done using a 79% dDDH threshold as previously introduced.[11]

Supplementary Table 4. NCBI accession numbers for genome and RNA-Seq sequences from PS_44_ISF1.

**Submission**	Acc. Nr.	Data	Note
Bio Project	PRJNA873226		Release date 01.09.2023
WGS - Genome	JANUXO000000000	ONT, PB, I	
SRA ONT	SRR21206837	ONT	
SRA PB	SRR21206836	PB	
SRA I	SRR21206835	I	
SRA RNA CSA	SRR21206834	I	
SRA RNA PDA	SRR21206833	I	

ONT: Oxford Nanopore, PB: Pac Bio, I: Illumina

Bacterial fatty acid analysis

Amycolatopsis sp. PS_44_ISF1 pre-cultures (50 ml) were prepared in ISP2 broth and cultivated for seven days at 30 °C and shaking at 150 rpm. ISP2 agar plates were inoculated with 100 µl of the pre-culture and further incubated at 30 °C (for media recipes, see Supplementary Table 2). After seven days of incubation, three biological replicates (n=3) of bacterial culture were harvested, frozen in liquid nitrogen and stored at -80 °C until shipment. Fatty acid content was determined by GCMS, conducted at the DSMZ. Predominant components of the fatty acid profile of *Amycolatopsis* sp. PS_44_ISF1 are the branched fatty acids *i*-$C_{15:0}$ (20.63, respectively), i-$C_{16:0}$ (14.17%), i-$C_{17:0}$ (10.34%) and ai-$C_{17:0}$ (9.40%), followed by saturated fatty acid $C_{16:0}$ (12.28%). Additionally, considerable amounts of hydroxylated and unsaturated fatty acids are detected, including 2-OH i-$C_{15:0}$ (4.44%), 2-OH i-C16:0 (1.48%) and C16:1 (4.75%).

Supplementary Table 5. Fatty acid composition of PS_44_ISF1 cultures and related *Amycolatopsis* strains according to Supplementary Methods 5.

fatty acid	PS_44_ISF1	**1** [26]	**2** [27]	**3** [28]	**4** [28]	**5** [29]	**6** [29]	**7** [29]	**8** [29]
C14:0 iso	1.55	9	6.4	3.6	1.8	2.2	4.4	1.8	9.8
C15:0 iso	**20.63**	9	22.5	5.7	11.2	14.3	13	11.2	8.7
C15:0 ISO 2OH	4.44	n.s.	8.7	n.s.	n.s.	n.s.	n.s.	n.s.	n.s.
C15:0 anteiso	2.48	n.s.	9.4	0.7	2.7	3.2	3.2	n.d.	n.d.
C16:0 iso	**14.17**	42	10.3	27.1	25	14.6	17.6	19.4	26.1
C16:1	4.75	n.s.	n.s.	n.s.	n.s.	0.9	n.d.	n.d.	4
C16:0	**16.25**	n.s.	3.3	7.2	11.3	20.2	16.2	8.8	10.2
C16:0 9-Methyl	3.64	n.s.	n.s.	n.s.	n.s.	n.s.	n.s.	n.s.	n.s.
C16:0 ISO 2OH	2.06	6	2.8	7.9	3.9	n.s.	n.s.	n.s.	n.s.
C17:0 iso	**10.34**	10	3.4	1.8	6.5	5	5	5.3	1.6
C17:0 anteiso	**9.40**	n.s.	n.s.	3.7	6.8	3.5	3.1	17.7	n.d.
C17:0	1.84	7	11.4	9.3	4.3	4.8	14.5	7.5	7.8
C18:0	3.11	n.s.	n.s.	3.3	1.8	17.5	13.2	6.1	6.2

1-*Amycolatopsis saalfeldensis* DSM 44993, 2-*A. decaplanina* DSM 44594, 3-*A. nigrescensis* DSM 44992, 4-*A. minnesotensis* NRRL B-24435, 5- *A. jejuensis* NRRL B-24427, 6- *A. halotolerans* NRRL B324428, 7- *A. sulphurea* IMSNU 20060, 8- *A. albidoflavus* IMSNU 22139. n.s. – not specified, n.d. not detected.

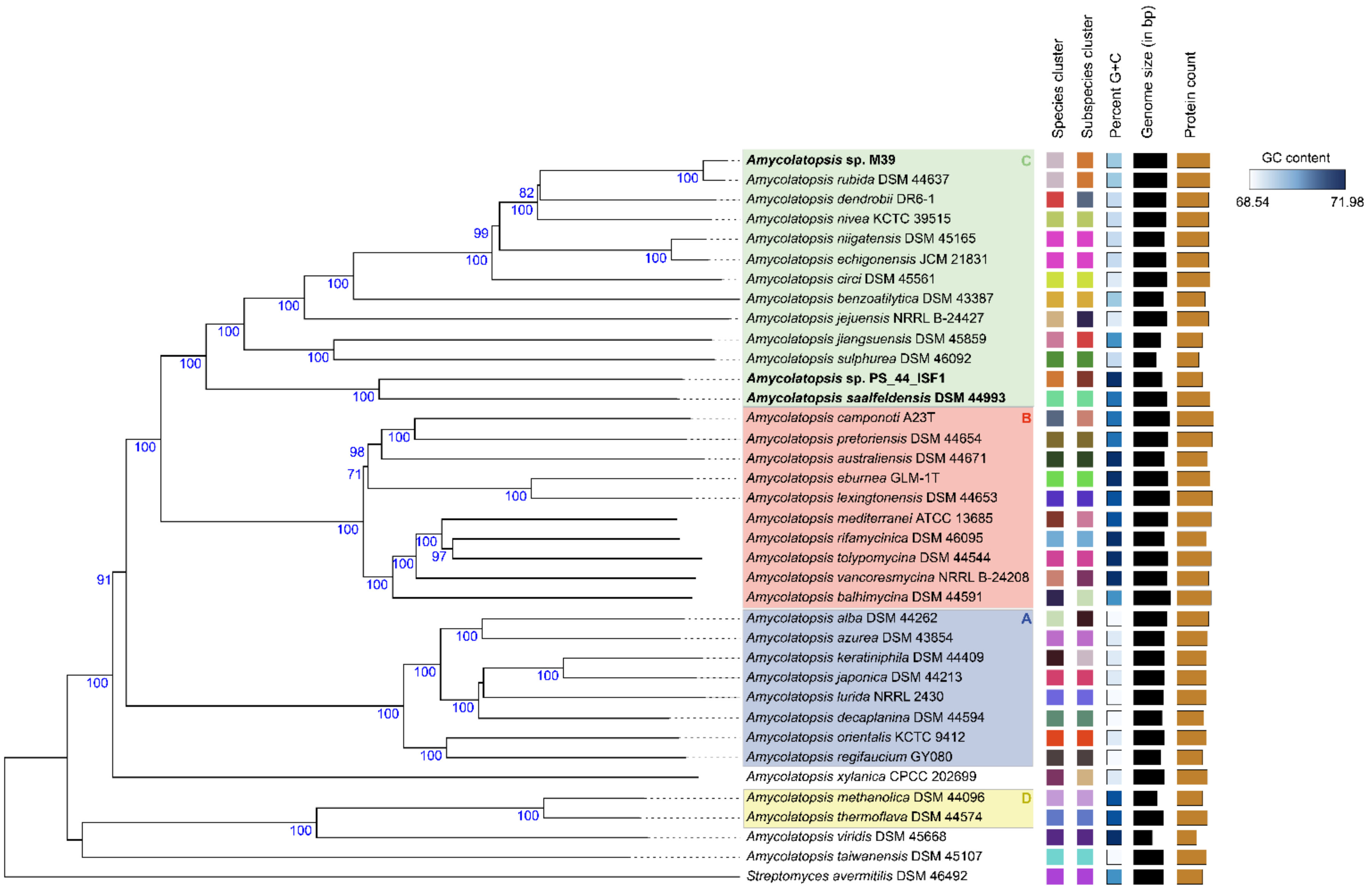

Supplementary Figure 2. Phylogenomic placement of *Amycolatopsis* sp. PS_44_ISF1. Tree inferred with FastME 2.1.6.1[19] from GBDP distances calculated from genome sequences. The branch lengths are scaled in terms of GBDP distance formula d_5. The numbers above branches are GBDP pseudo-bootstrap support values > 60 % from 100 replications, with an average branch support of 94.9 %. *Streptomyces avermitilis* DSM 46492 was used as outgroup. The four major phylogenetic lineages (A-D) of *Amycolatopsis*[21] are highlighted by colored boxes. Strains were assigned into a species and subspecies cluster by TYGS analysis[12] and GC-content, genome size and protein count were plotted for each strain. Phylogenomic groups (A-D) were adopted from Adamek et al.[13]

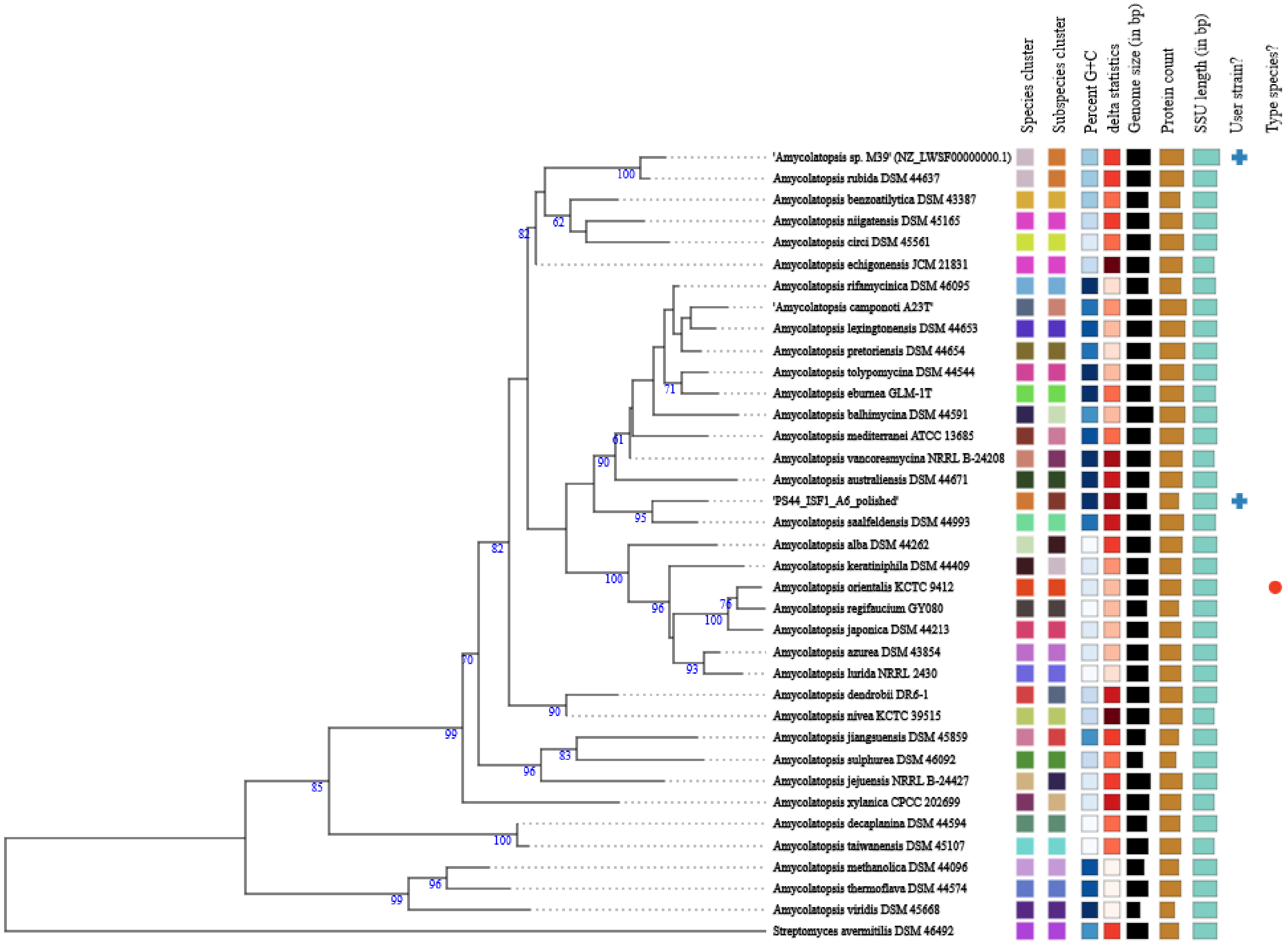

Supplementary Figure 3. Phylogenetic placement of *Amycolatopsis* sp. PS_44_ISF1 based on SSU. Tree inferred with FastME 2.1.6.1 from GBDP distances calculated from 16S rDNA gene sequences. The branch lengths are scaled in terms of GBDP distance formula d_5. The numbers above branches are GBDP pseudo-bootstrap support values > 60 % from 100 replications, with an average branch support of 69.6 %. The tree was rooted at the midpoint. *Streptomyces avermitilis* DSM 46492 was used as outgroup.

Assays for lipolytic activity.

To determine if *Amycolatopsis* sp. PS_44_ISF1 can survive in the oily environment of the bird's uropygial gland, it was screened for lipase and esterase activity. Pre-cultures of PS_44_ISF1 were grown for 7 days at 30 °C in ISP2 medium. For screening of esterase activity, a basal medium (900 mL) containing 10 g peptone, 5 g NaCl, 0.1 g $CaCl_2$ and 20 g agar was prepared, and the pH was set to 7 (modified after Gopinath et al. 2005).[14] Tween-20 (10 mL, Sigma-Aldrich) was dissolved in 100 mL of warm distilled water and autoclaved separately. After sterilization at 120 °C for 20 min, the medium was cooled to 50 °C and the Tween-20 solution was added. The mixture was stirred vigorously and poured in sterile petri dishes. Each plate was inoculated with 3 spots of 20 µl of PS_44_ISF1 pre-culture and incubated at 30 °C (n=3). After 7 days, lipolytic activity was indicated by the appearance of a visible precipitate, resulting from the formation of calcium salt formed by the fatty acid liberated by the enzyme.

As complementary method, chromogenic lipase substrate agar plates were prepared according to Singh et al. (2006).[15] Olive oil (Natives Olivenöl exta, Alnatura, Darmstadt) and lanolin (Carl Roth, Karlsruhe) were used as lipid/ wax substrate. Lanolin resembles more closely the composition of bird uropygial gland (UG) excretions, which mainly consist of long-chain diester waxes.[16] Phenol red is a pH indicator with an endpoint at pH 7.3-7.4 where it is pink, a slight decrease in pH to 7.1-7.0 turns it yellow.[17] The chromogenic agar was set to a pH of 7.4 by using NaOH. Each plate (n=3) was inoculated with 2 spots of 20 µl of *Amycolatopsis* sp. PS_44_ISF1 pre-culture and incubated at 30 °C for 7 days. Upon lipolysis, liberated fatty acids lead to a slight drop in pH and a color change to yellow is observed. Each treatment was prepared in triplicates.

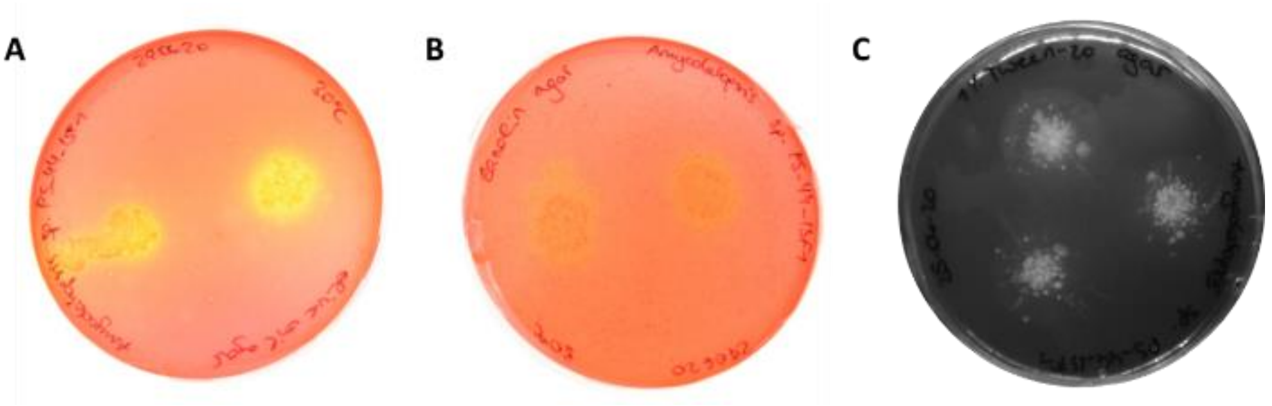

Supplementary Figure 4. Lipolytic activity of *Amycolatopsis* sp. PS_44_ISF1. A. Assay on lipolytic activity of PS_44_ISF1 on chromogenic agar containing olive oil and the pH indicator phenol red. Release of fatty acids by lipase activity leads to a slight drop in pH resulting in a color change of phenol red from red to yellow. **B**. Assay on lipolytic activity of *Amycolatopsis* sp. PS_44_ISF1 on chromogenic agar containing lanolin. C. Assay on esterase activity of *Amycolatopsis* sp. PS_44_ISF1 on $CaCl_2$-containinf screening agar containing Tween-20. Released fatty acids by esterase activity form salts with Ca^{2+} and are visible as white precipitate.

Assays for keratinolytic activity.

To screen for keratinolytic activity of *Amycolatopsis* sp. PS_44_ISF1, selective keratin agar plates (pH 7.5) were prepared, containing 20 g horn and hoof meal powder (Compo, Münster), 5 g peptone, 5 g yeast extract, 1 g H_2HPO_4, 0.2 g $MgSO_4$ x 7 H_2O and 18 g agar per liter water.[18] Pre-cultures of PS_44_ISF1 in ISP2 medium were grown for 7 days at 30 °C, then 50 µl of pre-culture were inoculated on selective keratin agar plates and incubated at 30 °C for 7 days. Upon keratinolysis, a zone of hydrolysis is visible around the bacterial colonies. In another screening approach, selective feather broth (FB, pH 7.2) was prepared, containing 0.5 g NaCl, 0.3 g K_2HPO_4, 0.4 g KH_2PO_4, 5 g finely chopped feathers (Rayher., Marbach am Neckar), 1 mL trace element solution (modified after Dada et al. (2019),[19] see Supplementary Table 2). The feather broth was inoculated with 100 µl of PS_44_ISF1 pre-culture in three biological replicates and incubated for 7 days at 30 °C. After the incubation period, lysis of the feathers was examined.

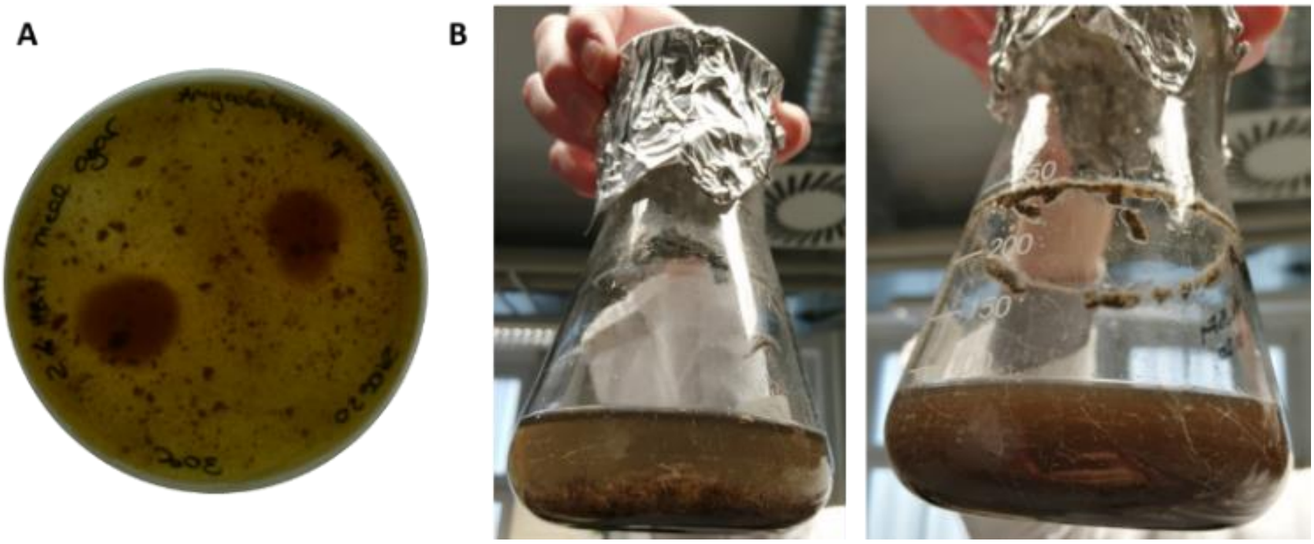

Supplementary Figure 5. Keratinolytic activity of PS_44_ISF1. A. Assay on keratinolytic activity of PS_44_ISF1 on 2% horn & hoof meal (H&H) agar. Halos around the bacterial culture indicate keratinolysis. **B.** Assay on keratinolytic activity of PS_44_ISF1 feather broth containing 0.5% finely chopped feathers after seven days of incubation (with n=3). **B Left**: Negative control without inoculation with PS_44_ISF1. Bird feathers are still intact. **B Right**: In the presence of strain PS_44_ISF1 feathers are completely digested to a fine powder.

General Analytical Methods.

Chemicals. If not stated differently, all media ingredients and chemicals were purchased from Carl Roth. Methanol (Th. Geyer, Renningen); water for analytical and preparative HPLC (Millipore, Germany), formic acid (Carl Roth, Germany); acetonitrile (Th. Geyer, Renningen), DMSO (Carl Roth, Germany).

Instruments: UV spectra were measured at 25 °C on a UV-2600 spectrophotometer (Shimadzu). Optical rotations were recorded in MeOH on a P-1020 polarimeter (JASCO). IR spectra were recorded on an FT/IR-4100 ATR spectrometer (JASCO). ^{1}H NMR (600MHz) and ^{13}C NMR (150 MHz) spectral data were performed on a Bruker AVANCE III 600 MHz spectrometer, equipped with a Bruker Cryo platform. The chemical shifts are reported in parts per million (ppm) relative to the solvent residual peak of CD_3OH-d_3 (^{1}H: 3.30 ppm; ^{13}C: 49.0 ppm) or DMSO-d_6 (^{1}H: 2.50 ppm; ^{13}C: 39.5 ppm). Semi-preparative HPLC was performed on a Shimadzu HPLC system using a Phenomenex Luna C18(2) 250 x 10 mm column (particle size 5 µm, pore diameter 100 Å).

HR-UPLC-ESI-MS/MS measurements: High resolution ultra-high performance liquid chromatography electrospray ionization mass spectrometry (HR-UPLC-ESI-MS) measurements were carried out on a Dionex Ultimate3000 system (Thermo Scientific) combined with a Q-Exactive Plus mass spectrometer (Thermo Scientific) equipped with an elecrospray ion (ESI) source and a Luna Omega C18 column (100 x 2.1 mm, particle size 1.6 µm, pore diameter 100 Å). The column oven was set to 40 °C, scan range of MS was set to *m/z* 200 to 2,000 with a scan speed of 10,000 u/s and event time of 0.25 s under positive mode. DL temperature was set to 250 °C with an interface temperature of 350 °C and a heat block of 400 °C. The nebulizing gas flow was set to 1.5 L/min and dry gas flow to 15 L/min. A combination of data dependent MS2 analysis and Top10 experiments was applied for MS2 measurements. MS2 measurements were performed in centroid mode with a resolving power of 17,500 at *m/z* 200, an isolation window of 1 *m/z* and stepped normalized collision energy of 20/30/40. Each sample was analysed using the following UHPLC gradient: 0-1 min, 5% B, 1-7 min, 5-97% B, 7-9 min, 97% B, 9-10 min; 97-5% B, 10-13 min, 5% B) with an injection volume of 5 µl and a flow rate of 0.3 mL/min. All eluents of the UHPLC system were acidified with 0.1 % formic acid. H_2O was used as eluent A while acetonitrile was used as eluent B.

For each analytical runs, washing steps and blank runs were included (at least every 5 sample) and served as control samples to check for cross-contamination. For each biological sample, one technical replicate was measured. For structure elucidation MS/MS spectra were predicted using CFM-ID 4.0 or MassFrontier 8.0 (Thermo Fisher Scientific) and compared to the experimental MS/MS spectra.

Analysis of bacterial co-cultivation studies.

Amycolatopsis sp. PS_44_ISF1 was cultivated on MS agar for 7 days at 30 °C. After cultivation, a PS_44_ISF1 spore solution was prepared according to the procedure of Kieser et al. (2000).[20] The resulting spore solution was set to an OD_{600} of 0.1 and two spots (5 µl) of this solution was placed onto a fresh PDA agar plate. Plates were incubated at 30 °C for 2 days, then 5 µl of a freshly grown culture (adjusted to an OD600 of 0.1) of different competitor strains were added in proximity (approximately 1 cm radius) of the *Amycolatopsis* culture. Competitor strains included feather-degrading bacteria *Bacillus licheniformis* DSM13, *Pseudomonas monteilii* DSM1388, *Kocuria rhizophila* DSM11926, and non-degrading feather isolates *B. thuringiensis* DSM104061 and *Staphylococcus epidermidis* DSM103867. Incubation at 30 °C was continued for another 7 days and photos were taken after 3 and 7 days of incubation. After seven days of incubation, different areas (A-D, Figure 12 and Figure 13) of the co-cultivation plates, control plates and PDA control plates were cut out and transferred into a pre-weighed 50 mL beaker. The sample weight was determined, and the sample was extracted with MeOH overnight at room temperature, and the solvent was filtered and concentrated *in vacuo*. Each sample was dissolved in 100% MeOH to a final concentration of 50 µg/ml and subjected to HR-UPLC-ESI-MS analysis. The production of each metabolite of interest was evaluated by the comparison of the peak area using the extracted ion mode (EIC) and normalized to the wet weight of agar extracted (technical replicate: n=1; peak area/mg wet weight).

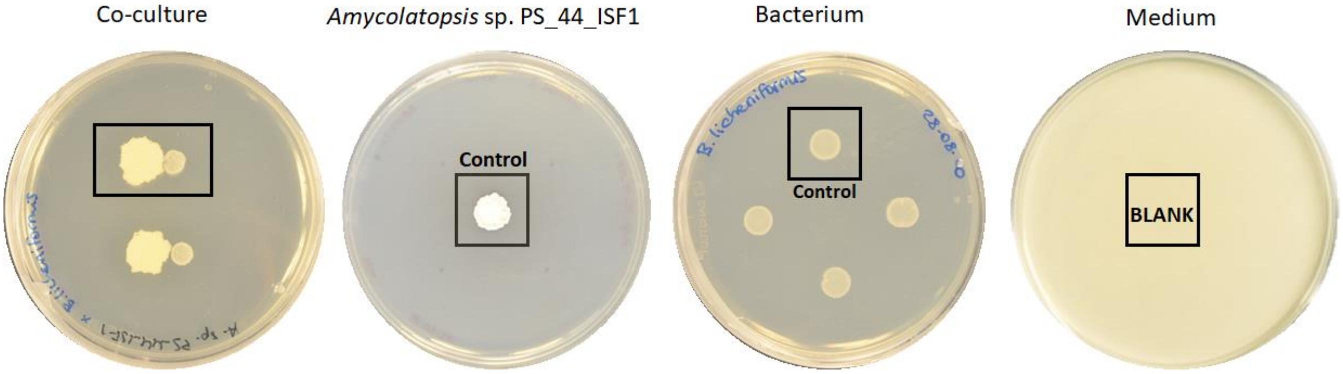

Supplementary Figure 6. Set-up of bacterial co-cultivation studies. From left to right: Co-cultivation plates with *Amycolatopsis* sp. PS_44_ISF1 (left) and bacterial competitor (right), axenic controls of *Amycolatopsis* sp. PS_44_ISF1, the bacterial competitor (here *Bacillus licheniformis* as an example) and a medium control plate (PDA). A-D represent the areas used for further extraction and HRMS analysis.

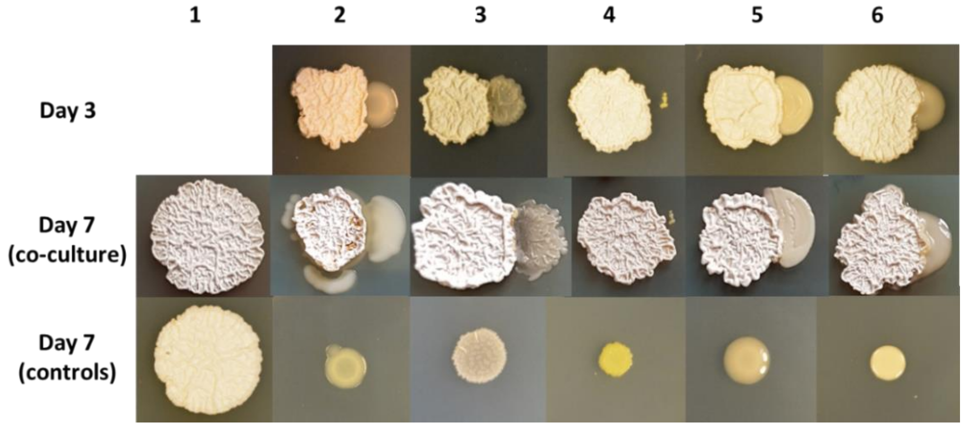

Supplementary Figure 7. Co-cultivation of *Amycolatopsis* sp. PS_44_ISF1 with feather-degrading and non-degrading bacterial feather pathogens. Representative plates of co-cultures of *Amycolatopsis* sp. PS_44_ISF1 (1) with *Pseudomonas monteilii* (2), *Bacillus licheniformis* (3), *Kocuria rhizophila* (4), *Bacillus thuringiensis* (5) and *Staphylococcus epidermidis* (6) after three and seven days of incubation (n=3). Axenic cultures of strains 1-6 served as controls (day 7, controls).

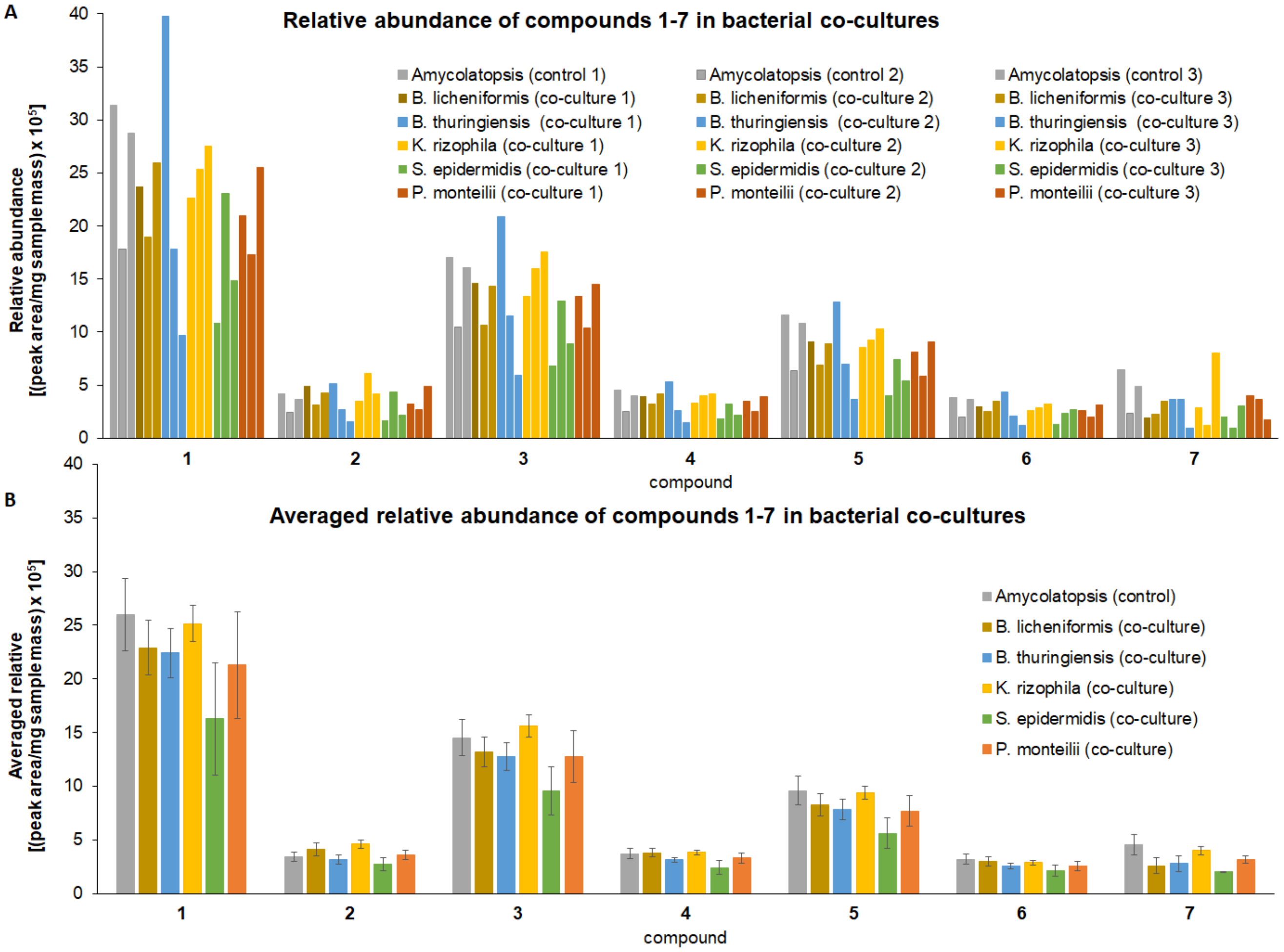

Supplementary Figure 8. Relative abundance of compounds 1-7 in co-cultures of *Amycolatopsis* sp. PS_44_ISF1 with bacterial feather pathogens.

A) Relative abundance of compounds **1-7** from axenic control culture (grey) and coculture with *Bacillus licheniformis* (brown), *Bacillus thuringiensis* (blue), *K. rhizophila* (yellow), *Staphylococcus epidermidis* (green) and *Pseudomonas monteilii* (brown) (data of each biological replicate is shown as single data point, relative peak area/mg wet weight), and B) averaged abundance of compounds **1-7** calculated from three biological replicates (n = 3 ± standard deviation error). Samples were harvested after seven days of incubation (Supplementary Figure 6) and analysed for EICs ($[M+H]^+$ ± 5 ppm/normalized to the weight of the extracted agar) for **1** (731.4662), **2** (745.4818), **3** (759.4975), **4** (773.5131), **5** (787.5288), **6** (801.5444), and **7** (694.3519). Source data are provided as a Source Data file.

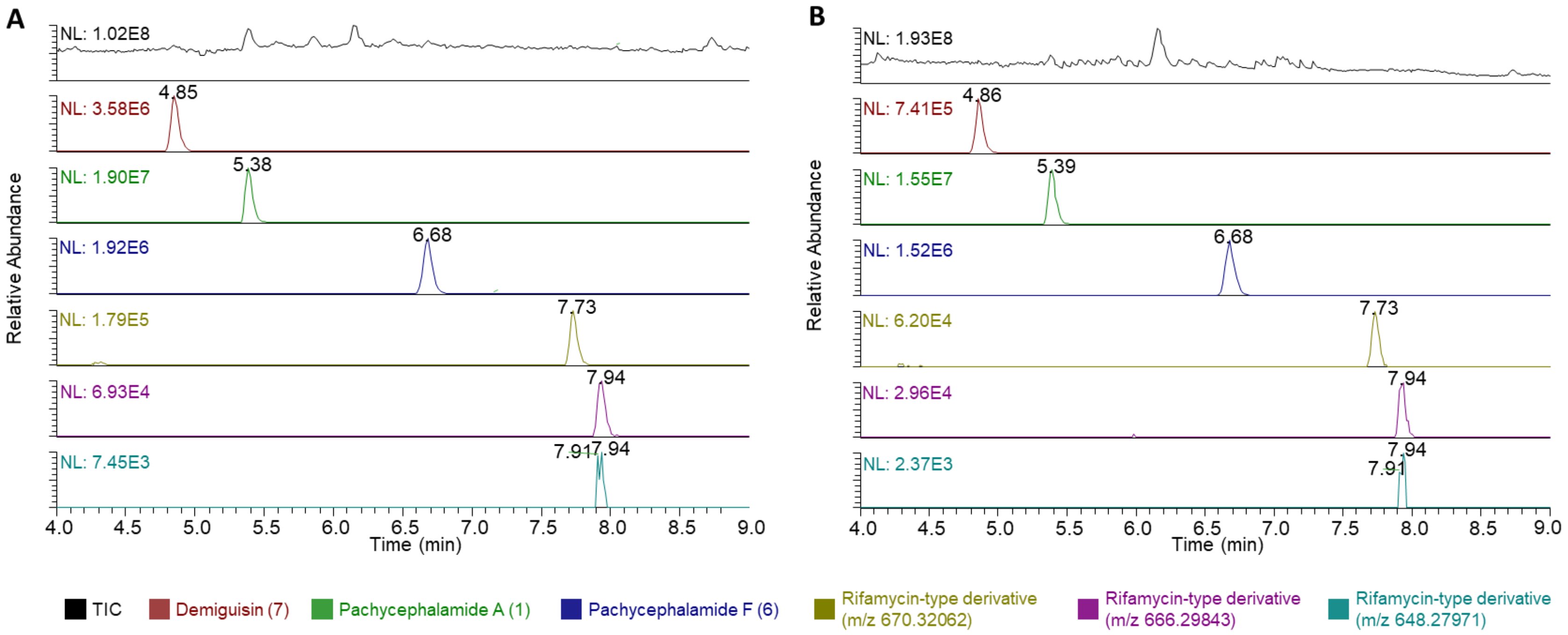

Supplementary Figure 9. Representative examples of targeted analysis of secreted metabolites during co-cultivation of PS_44_ISF1 with *K. rhizophila*. Extracted ion chromatograms (EICs with $[M+H]^+ \pm 5$ ppm)) of demigusin (red, $[M+H]^+$ 694.3519), pachycephalamides A (green, $[M+H]^+$ 731.4662) and F (blue, $[M+H]^+$ 801.5444) and three derivatives assigned to the rifamycin family (yellow, purple, turquoise) produced in axenic controls of PS_44_ISF1 (**A**) and in co-culture with *K. rhizophila* (**B**).

Analysis of fungal co-cultures.

50 µl of a 7-day-old *Amycolatopsis* sp. PS_44_ISF1 pre-culture in ISP2 were inoculated at the center of a PDA agar plate (150 x 20 mm) and incubated for 7 days at 30 °C. Due to different growth rates and behavior, pre-cultures of fungal competitor strains were prepared at different time points. On the same day, *Pseudoxylaria* sp. X802 was transferred on fresh PDA medium. After three days of incubation, cultures of *Aspergillus niger* N402 and *A. fumigatus* FGSC A1160 on PDA were prepared, *Candida albicans* SC5314 pre-cultures were inoculated in YPD medium from a cryostock on the last day of incubation. After 7 days of incubation, competitor strains were added in proximity to the *Amycolatopsis* culture. In case of *Pseudoxylaria*, 1 cm^2 cubes of the 7-day-old culture were cut out and placed at four positions surrounding the bacterial culture. For *Aspergillus* strains, spore solutions of the 5-day-old cultures were prepared in YPD broth ($1x10^6$ spores/mL) and 10 µL of the spore solution was placed at four positions surrounding the bacterial culture. For *C. albicans*, the overnight culture was inoculated into 5 ml of YPD and incubated for 4-5 h at 37 °C and 180 rpm. Afterwards, the culture was diluted to an OD_{600} of 0.1, and the resulting cell suspension was streaked three times over the entire plate (using a sterile cotton swab), also touching the *Amycolatopsis* culture. After adding the competitor strains, incubation was continued for 2 days for *C. albicans* co-cultures and for 7 days for all other fungal co-cultures, and photos were taken every day. Axenic cultures of *Amycolatopsis* sp. PS_44_ISF1 and each competitor strain inoculated at the same time as the co-cultures served as controls. Each condition was performed in triplicates (n=3). Afterwards, different areas (A-G, Supplementary Figure 10) of the co-cultivation plates, control plates and PDA control plates were cut out and transferred into a pre-weighed 50 mL beaker. The sample weight was determined, and the sample was extracted with MeOH. After overnight extraction at room temperature, the solvent was filtered and concentrated *in vacuo*. Each sample was dissolved in 100% MeOH to a final concentration of 50 µg/ml and subjected to HR-UPLC-ESI-MS analysis. The production of each metabolite of interest was evaluated by the comparison of the peak area using the extracted ion mode (EIC) and normalized to the wet weight of agar extracted (technical replicate: n=1; peak area/mg wet weight).

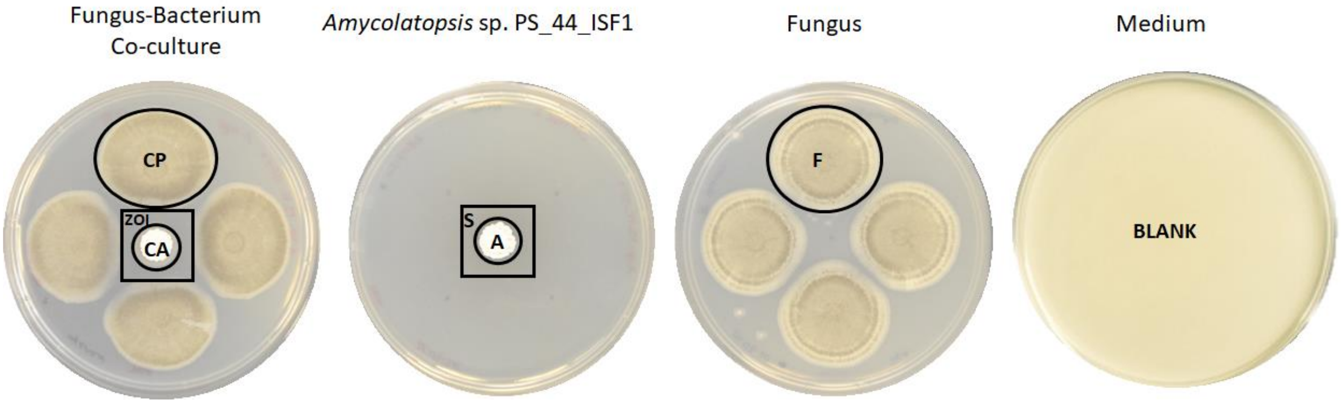

Supplementary Figure 10. Set-up of fungal co-cultivation studies. From left to right: Co-cultivation plates with *Amycolatopsis* sp. PS_44_ISF1 in the center surrounded by fungal competitor at four positions, axenic controls of *Amycolatopsis* sp. PS_44_ISF1, the fungal competitor (here *A. niger* as an example) and a medium control plate (PDA). A-G represent the areas used for further extraction and HRMS analysis. CA: Co-culture *Amycolatopsis* sp. PS_44_ISF1, ZOI: Zone of inhibition, CP: Co-culture fungus, S: surrounding agar in control, A: *Amycolatopsis* sp. PS_44_ISF1, F: fungus.

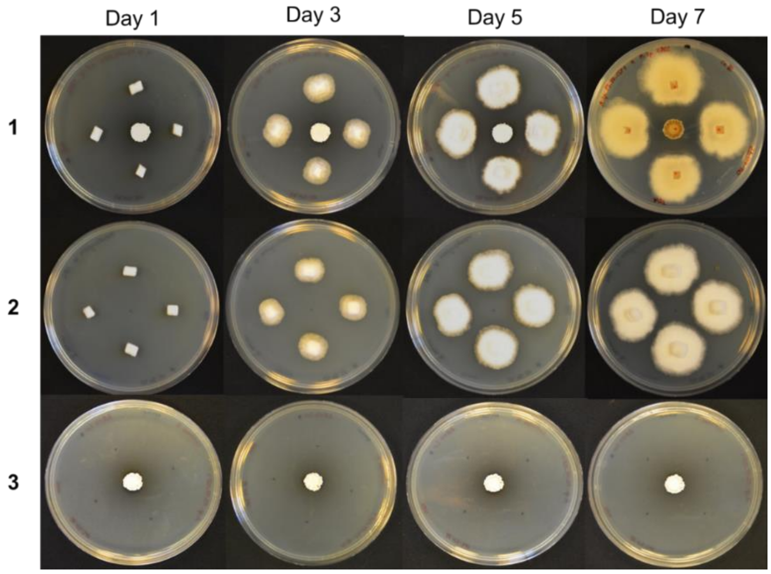

Supplementary Figure 11. Co-cultivation of *Amycolatopsis* sp. PS_44_ISF1 with *Pseudoxylaria* sp. X802. Representative co-cultures (1) and axenic competitor (2) and *Amycolatopsis* controls (3) (n=3). Photos were taken every second day.

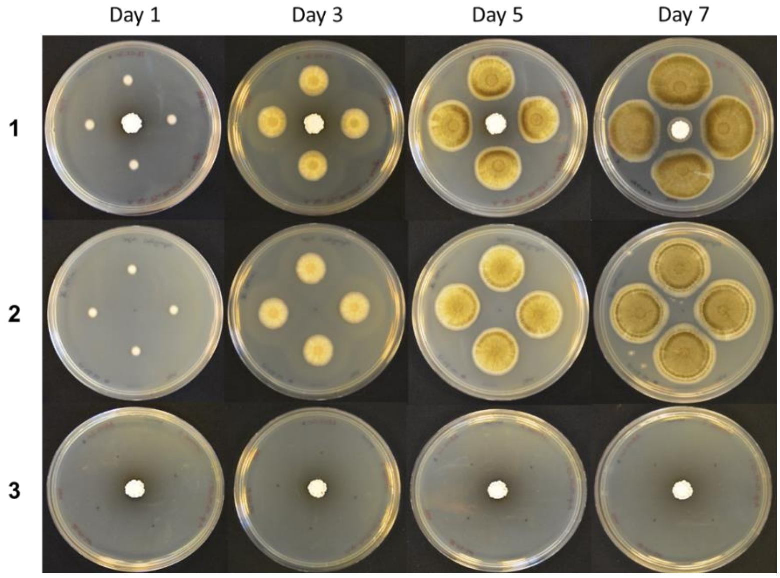

Supplementary Figure 12. Co-cultivation of *Amycolatopsis* sp. PS_44_ISF1 with *Aspergillus niger* N402. Representative co-cultures (1) and axenic competitor (2) and *Amycolatopsis* controls (3) (n=3). Photos were taken every second day.

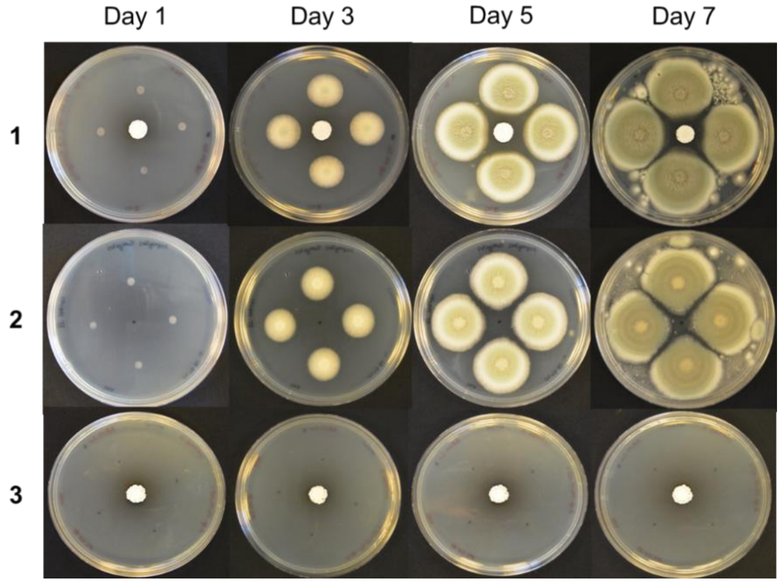

Supplementary Figure 13. Co-cultivation of *Amycolatopsis* sp. PS_44_ISF1 with *Aspergillus fumigatus* FGSC A1160. Representative co-cultures (1), and axenic competitor (2) and *Amycolatopsis* controls (3) (n=3). Photos were taken every second day.

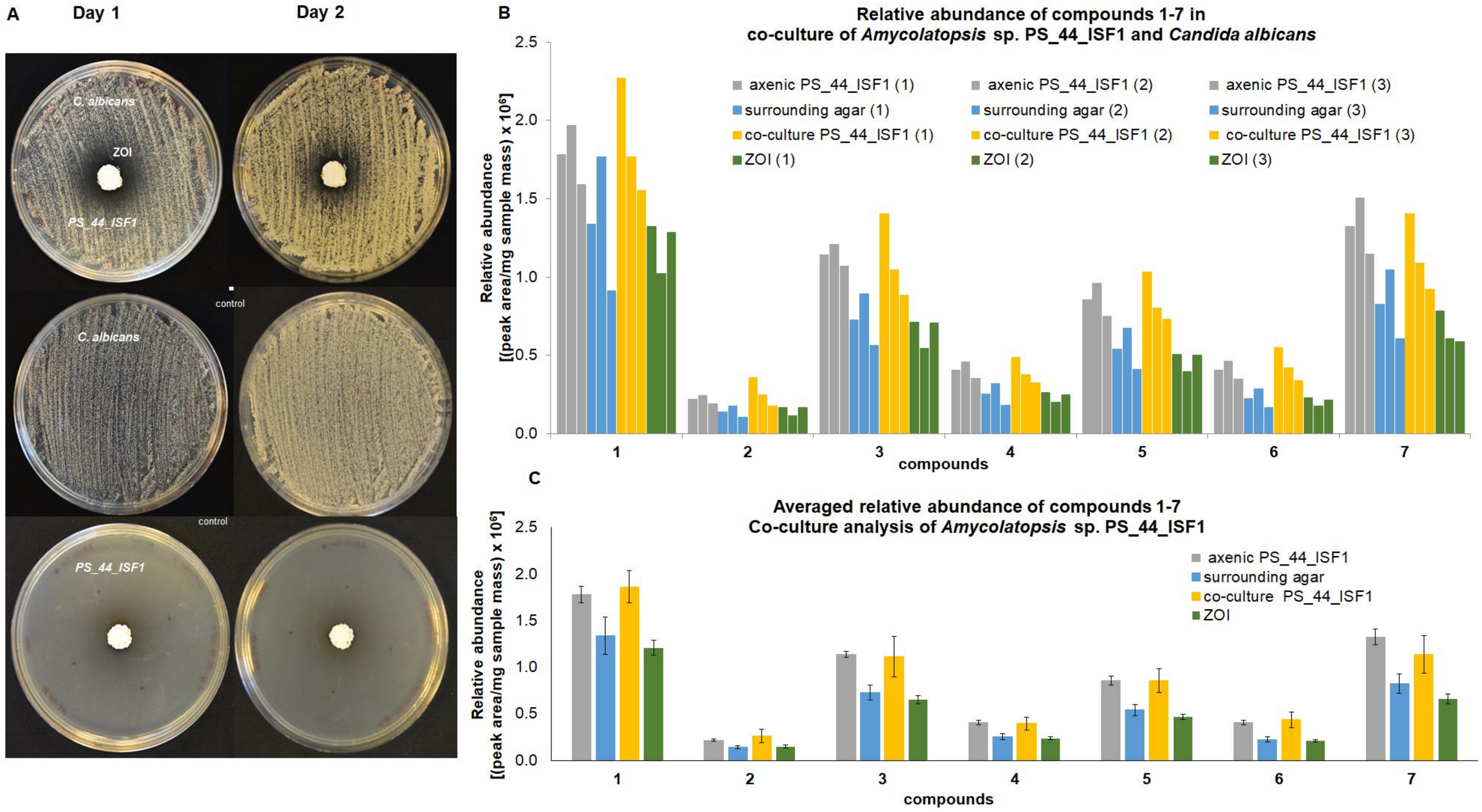

Supplementary Figure 14. Relative abundance of compound 1-7 in co-cultivation of *Amycolatopsis* sp. PS_44_ISF1 with *Candida albicans*. A) Representative pictures of co-culture, axenic competitor *C. albicans*, and axenic culture of *Amycolatopsis* sp. PS_44_ISF1 after one and two days of incubation (n =3).
B) Relative abundance of compounds **1**-**7** (data of each biological replicate is shown as single data point, relative peak area/mg wet weight) in an axenic culture of *Amycolatopsis* (grey) and surrounding agar (blue), compared to *Amycolatopsis* in co-culture with *C. albicans* (yellow), and in the inhibition zone (ZOI, green), and C) averaged abundance of compounds **1**-**7** (average peak area/mg wet weight ± standard error, n = 3) in an axenic culture of *Amycolatopsis* (grey) and surrounding agar (blue), compared to *Amycolatopsis* in co-culture with *C. albicans* (yellow), and in the inhibition zone (ZOI, green).
Abundance was calculated from peak areas extracted from EICs ($[M+H]^+$ ± 5 ppm) for **1** (731.4662), **2** (745.4818), **3** (759.4975), **4** (773.5131), **5** (787.5288), **6** (801.5444), and **7** (694.3519). Source data are available in the Source Data file.

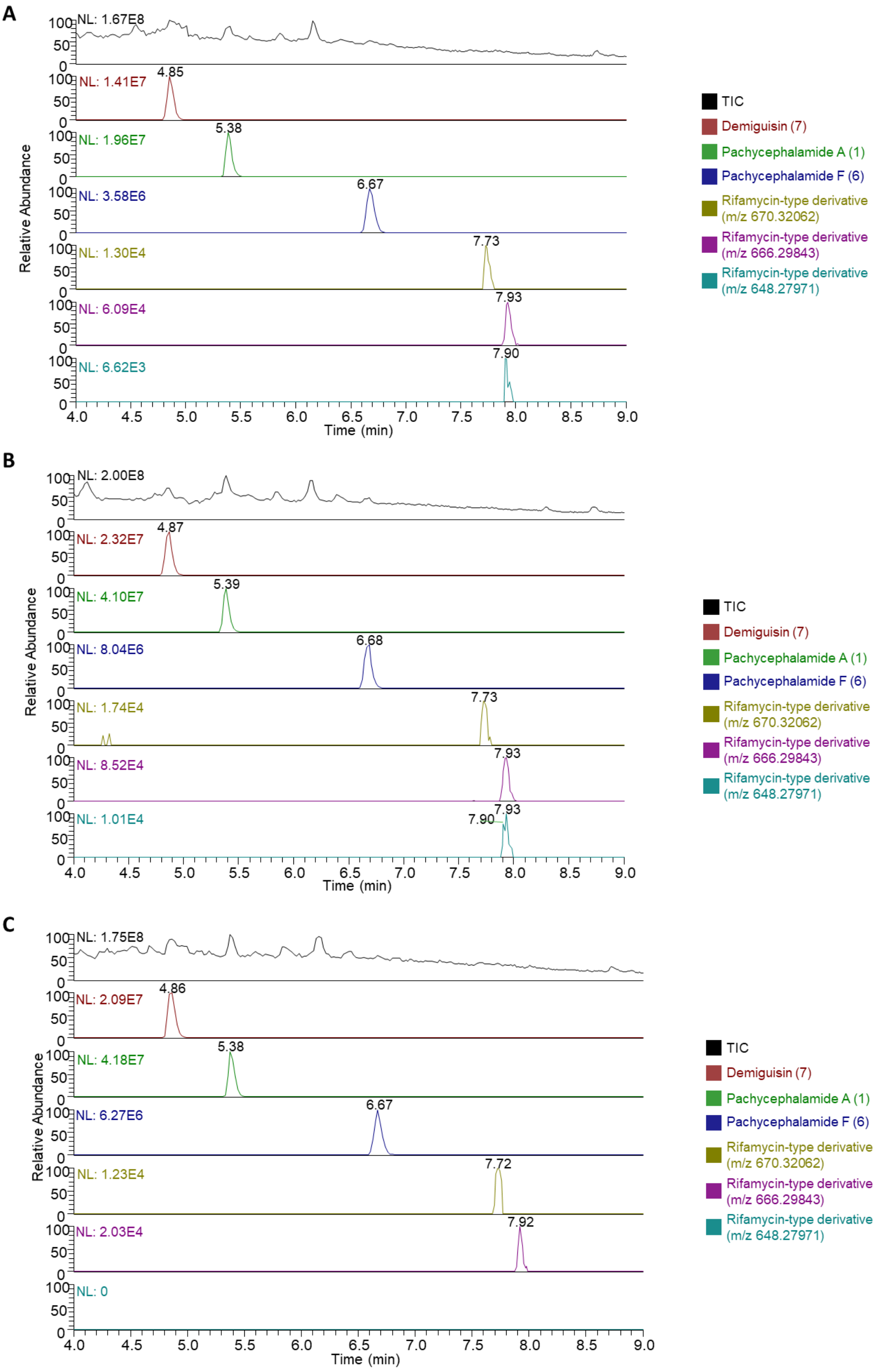

Supplementary Figure 15. Secreted metabolites during co-cultivation of PS_44_ISF1 with *C. albicans*. Extracted ion chromatograms (EICs) of demigusin (red), pachycephalamides A (green) and F (blue) and three derivatives assigned to the rifamycin family (yellow, purple, turquoise) produced in axenic controls of PS_44_ISF1 (A), in co-culture with *C. albicans* (B) and secreted in the inhibition zone (C) with n=3.
Abundance was calculated from peak areas extracted from EICs ($[M+H]^+$ ± 5 ppm) for **1** (731.4662), **2** (745.4818), **3** (759.4975), **4** (773.5131), **5** (787.5288), **6** (801.5444), and **7** (694.3519).

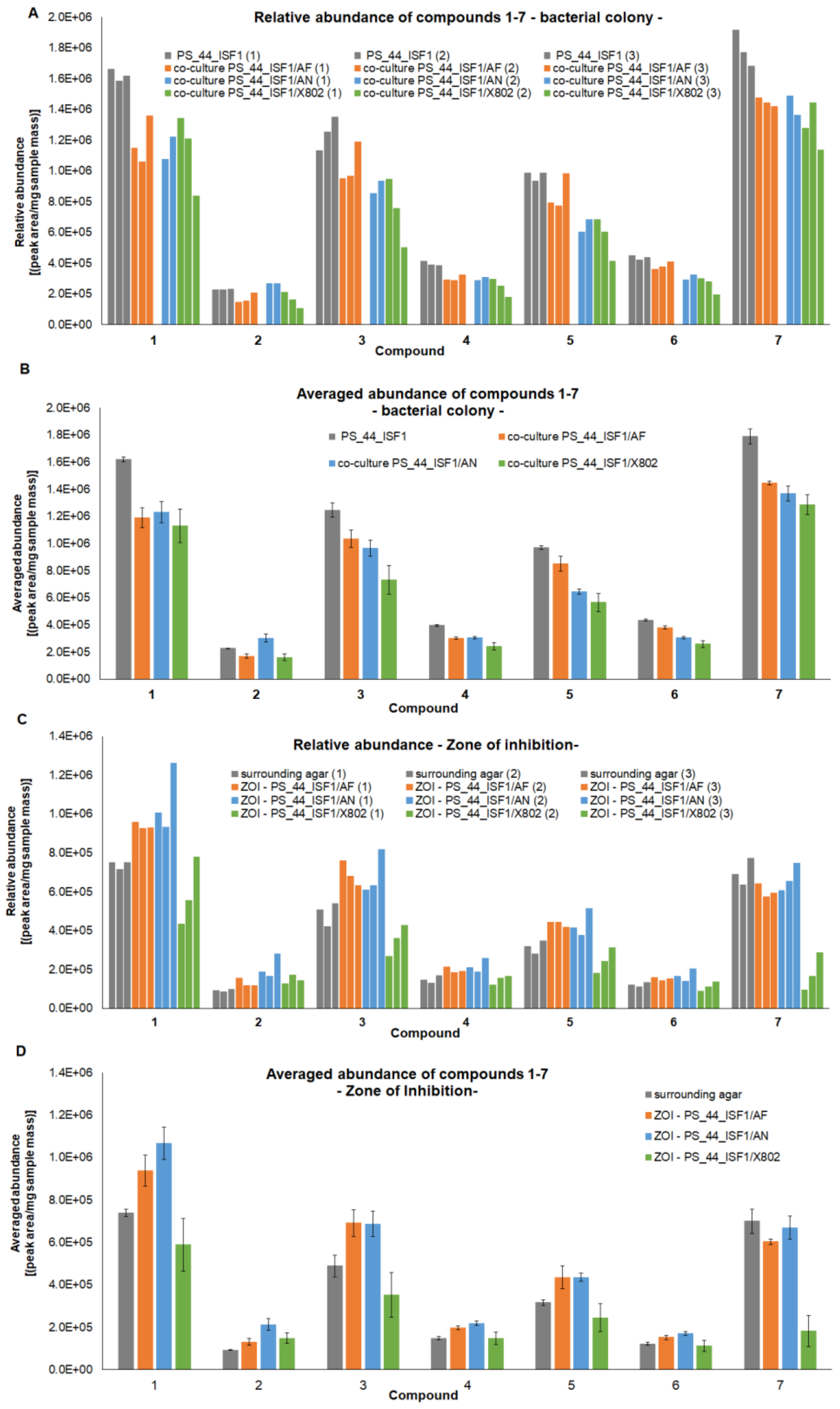

Supplementary Figure 16. Abundance of compounds 1-7 in bacterial colony of *Amycolatopsis* sp. PS_44_ISF1. A) Relative abundance of compounds **1**-**7** in axenic bacterial colony and in co-culture with *Aspergillus fumigatus* (AF), *Aspergillus niger* (AN) and *Pseudoxylaria* sp. X802 (802) (data of each biological replicate is shown as single data point, peak area/mg wet weight), and B) averaged abundance in axenic bacterial colony and in co-culture (n=3 ± standard error). C) Relative abundance of compounds **1**-**7** within the surrounding agar (axenic culture) and zone of inhibition (ZOI) in co-culture with *Aspergillus fumigatus* (AF), *Aspergillus niger* (AN) and *Pseudoxylaria* sp. X802 (802) (data of each biological replicate is shown as single data point, peak area/mg wet weight), and D) averaged abundance (n=3 ± standard error).

Abundance was calculated from peak areas extracted from EICs ($[M+H]^+$ ± 5 ppm) for **1** (731.4662), **2** (745.4818), **3** (759.4975), **4** (773.5131), **5** (787.5288), **6** (801.5444), and **7** (694.3519), and normalized to the weight of the extracted agar. Source data are available in the Source Data file.

MALDI-IMS of co-cultivation experiment.

Amycolatopsis sp. PS_44_ISF1 was cultivated on MS agar for 7 days at 30 °C. Indium-Tin-Oxide (ITO)-coated slides (IntelliSlides, Bruker) were coated by aluminum foil and sterilized by autoclaving. Slides were placed into a petri dish (90x16 mm) and 10 ml of PDA were distributed evenly on top of the slide. After cultivation, a PS_44_ISF1 spore solution was prepared according to the procedure of Kieser et al. (2000).[20] The resulting spore solution was set to an OD_{600} of 0.1 and 5 µl of this solution was placed in the center of the PDA-coated slides. *Amycolatopsis* cultures were incubated at 30 °C for a total period of six days. After one (= day 2) and three days (= day 4) of incubation 5 µl of a competitor strain culture or spore solution (OD_{600} = 0.1) was added in a proximity of 1 cm to the *Amycolatopsis* culture. Competitor strains used included three feather-degrading bacteria (*B. licheniformis* DSM13, *Kocuria rhizophila* DSM11926, *Pseudomonas monteilii* DSM1388), a filamentous fungus (*Aspergillus niger*) and the yeast *C. albicans* SC5314. Incubation of co-cultures were pursued until the end of the experiment. Axenic cultures of *Amycolatopsis* sp. PS_44_ISF1 and each competitor strain inoculated at the same time as the co-cultures served as controls.

Cultures on Indium-Tin-Oxide (ITO)-coated slides were dried for 5h at 37 °C. The dried samples were then sprayed with a saturated solution (20 mg/mL) of universal MALDI matrix (1:1 mixture of 2,5-dihydroxybenzoic acid and α-cyano-4-hydroxy-cinnamic acid; Bruker Daltonics, Bremen Germany) prepared in acetonitrile/methanol/water (70:25:5, v/v/v), using the automatic system ImagePrep device 2.0 (Bruker Daltonics, Bremen Germany) in 60 consecutive cycles (the sample was rotated 180° after 30 cycles) of 41 s (1 s spraying, 10 s incubation time, and 30 s of active drying) similar to Hoffmann and Dorrestein (2015).[21] The sample was analyzed in an UltrafleXtreme MALDI TOF/TOF (Bruker Daltonics, Bremen Germany), which was operated in positive reflector mode using flexControl 3.0. The analysis was performed in the 100-3000 Da range, with 30% laser intensity (laser type 4), accumulating 1000 shots by taking 50 random shots at every raster position. Raster width was set at 300 µm. Calibration of the acquisition method was performed externally using Peptide Calibration Standard II (Bruker Daltonics, Bremen Germany) containing Bradykinin1-7, Angiotensin II, Angiotensin I, Substance P, Bombesin, ACTH clip1-17, ACTH clip18-39, and Somatostatin 28. Spectra were processed with baseline subtraction in flexAnalysis 3.3 and aligned using several endogenous peaks (compounds present in the culture media). Processed spectra were uploaded in flexImaging 3.0 for visualization and SCILS Lab 2015b for analysis and representation. Chemical images were obtained using Total Ion Count normalization and medium denoising. The color map used in these images is called Viridis and is scientifically derived color map developed to provide an undistorted representation of the data, as well as be accessible to persons experiencing color-blindness.[22] Before analysis, a methanolic extract of PS_44_SF1 was measured using to concentrations (50 µg/mL and 1 mg/mL) to evaluate how the target compounds ionize in the MALDI-MS and which molecular ions (adducts) are predominantly observed. Compounds **1**-**6** were the most abundant metabolites while compound **7** was not reliably detected in the measured samples. Although we observed well ionization in ESI-HRMS for all compounds, compound **7** did not ionize as well in the MALDI-MS, which may have led to an underrepresentation of this compound in the dataset. In axenic cultures, a radial distribution of **1**-**7** was observed around PS_44_ISF1 colonies. In co-culture, compounds **1**-**6** show higher abundance at the interaction zone between the two interaction partners (e.g. Supplementary Figure 17, A_c, D_c, E_c). In other cases, compounds were enriched at the opposite side of the PS_44_ISF1 colony. As 2D-MALDI-IMS only measures the ion abundance on the surface of the agar and the MALDI laser is not able to penetrate bacterial colonies, we have to account for limitations regarding the depth profile of secreted metabolites throughout the agar medium.[23]

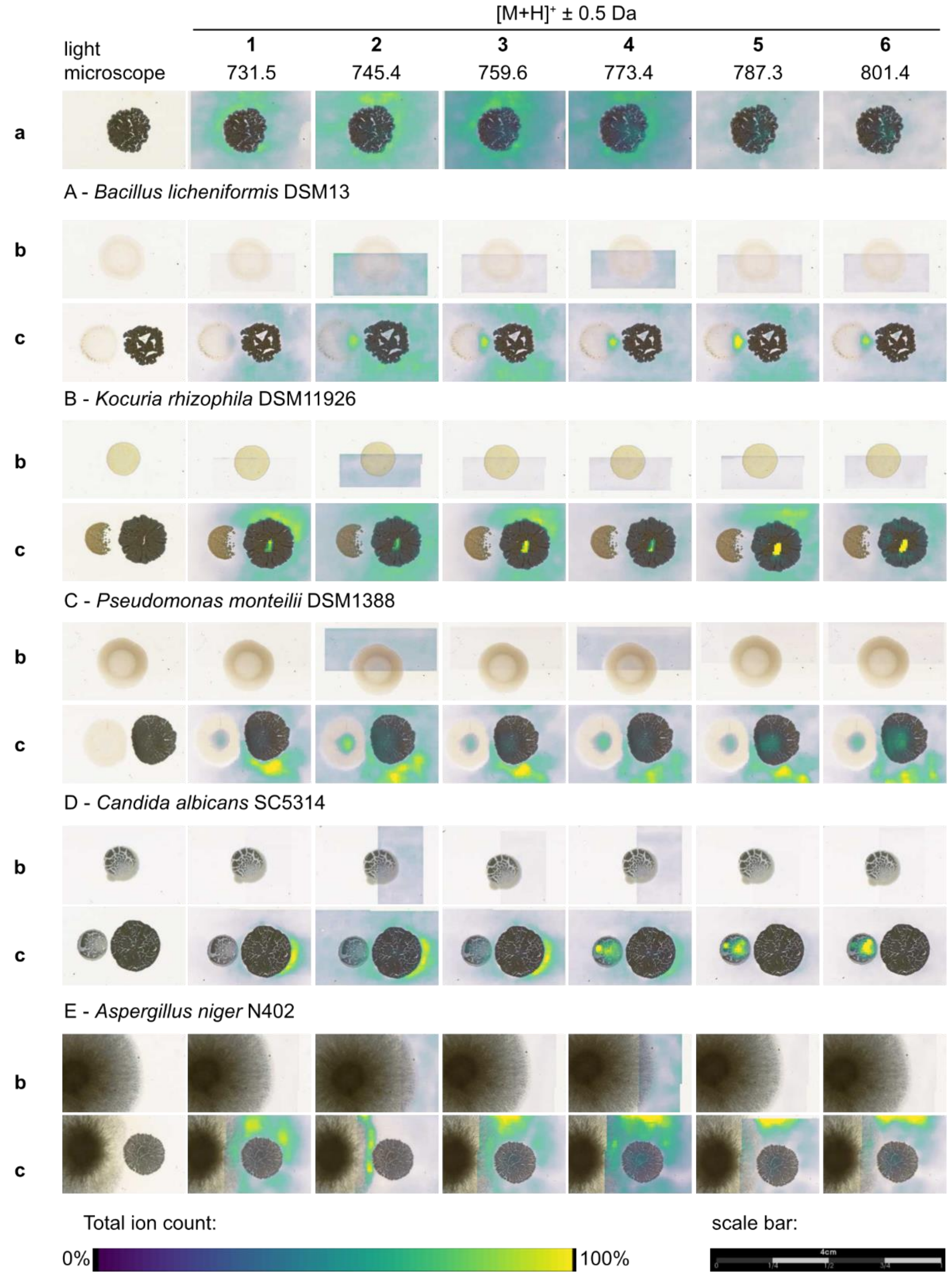

Supplementary Figure 17. MALDI imaging analysis of pachycephalamide (1-6) production during co-cultivation of *Amycolatopsis* sp. PS_44_ISF1 with feather-degrading bacterial and fungal competitors. *Amycolatopsis* was incubated for a total period of 6 days. Competing strains were added on day 2 and 4, and co-cultures were further incubated until the end of the experiment. MALDI-IMS was conducted for each sample (n=3) and chemical images of lipopeptide production ($[M+H]^+$ ± 0.5 Da) were obtained by total ion count normalization and medium denoising. Competing strains included three feather degrading bacteria (A – *B. licheniformis*, B – *K. rhizophila*, C – *P. monteilii*), the yeast *C. albicans* (D) and the filamentous fungus *A. niger*. Samples included axenic controls of *Amycolatopsis* sp. PS_44_ISF1 (a) and each competing strain (b) as well as the co-cultures thereof (c). Chemical images were taken for both treatments (addition of the competing strain on day 2 and on day 4), however, as the results were comparable, only one experiment is visualized here.

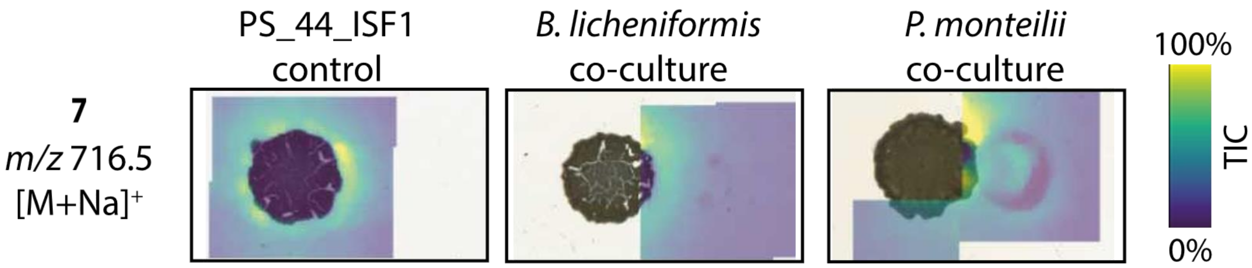

Supplementary Figure 18. MALDI imaging analysis of demiguisin (7) production during co-cultivation of *Amycolatopsis* sp. PS_44_ISF1 with feather-degrading bacterial competitors. *Amycolatopsis* was incubated for a total period of six days. Competing strains were added on day 2 and 4, and co-cultures were further incubated until the end of the experiment. Competing strains included three feather degrading bacteria (*B. licheniformis*, *K. rhizophila*, *P. monteilii*), the yeast *C. albicans* and the filamentous fungus *A. niger*. MALDI-IMS was conducted for each sample and chemical images of lipopeptide production ($[M+Na]^+$ ± 0.5 Da) were obtained by total ion count normalization and medium denoising. Compound **7** did not ionize well in the MALDI-IMS (Supplementary Figure 19), therefore, it was not detected in all measured samples.

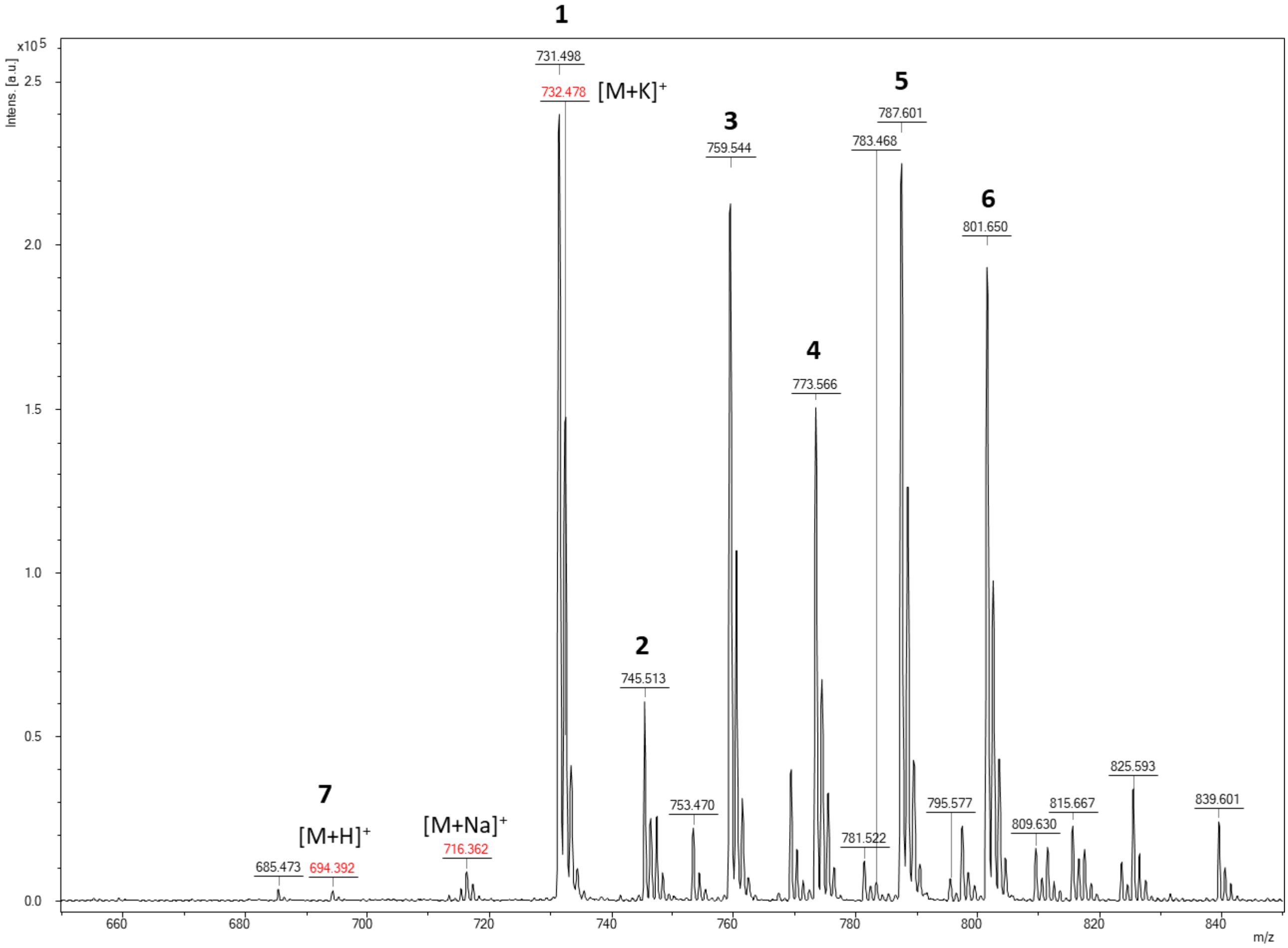

Supplementary Figure 19. Ionisation of compounds 1-7 in MALDI-IMS. The *m/z* values of demiguisin (7) ions are highlighted in red. While compounds **1**-**6** (m/z 731.498 – 201.650) ionized well with high peak intensities of the $[M+H]^+$ ion, peak intensities of compound 7 were low and m/z 716.632 $[M+Na]^+$ was used for analysis.

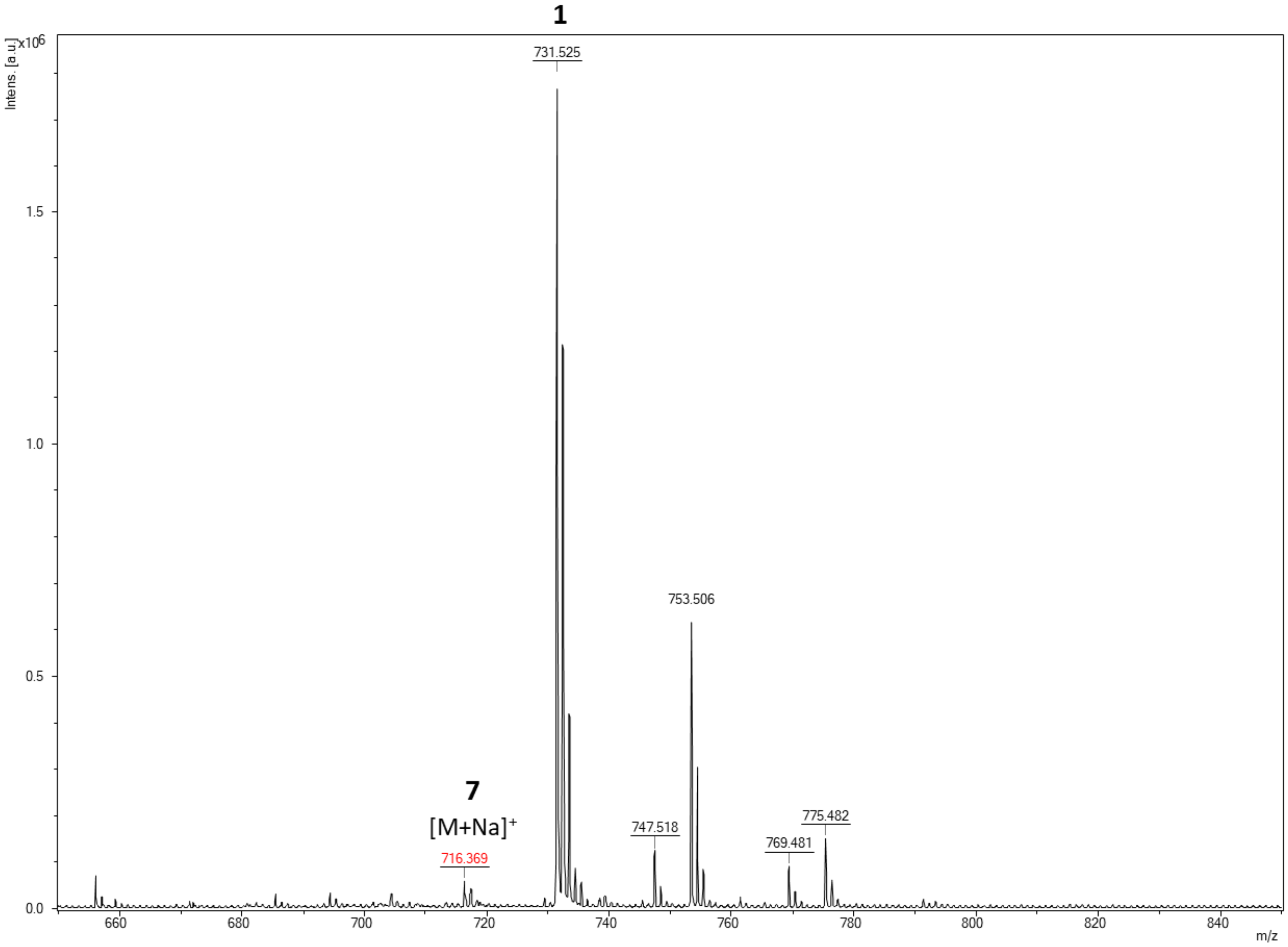

Supplementary Figure 20. MALDI measurement of demiguisin (7) and pachycephalamide A (1) in a mixture (1:1). Demiguisin and pachycephalamide A were dissolved in 50% MeOH (final concentration: 1 mg/ml). The *m/z* values of demiguisin ions are highlighted in red.

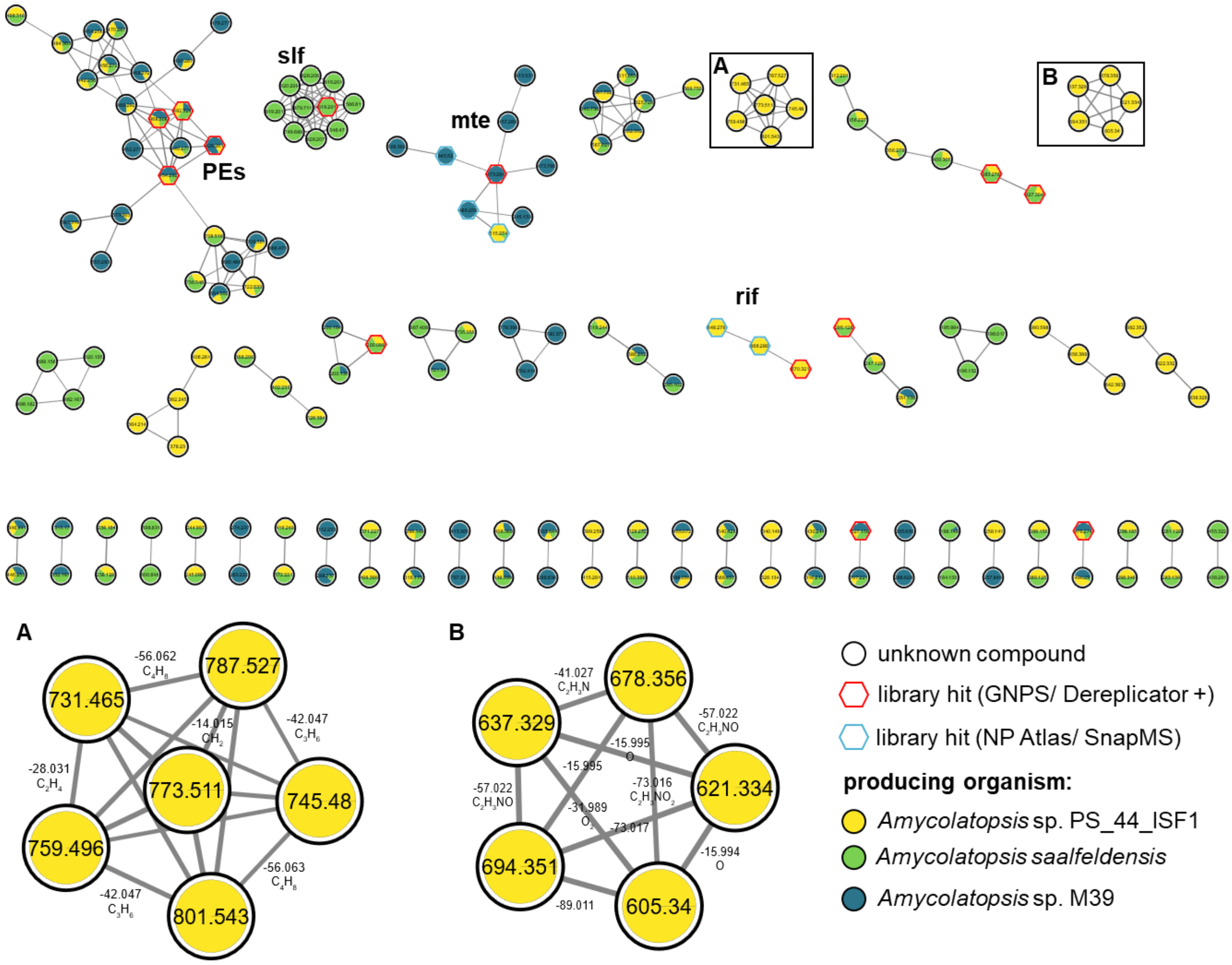

Supplementary Figure 21. Comparative metabolic network analysis of metabolites produced by *Amycolatopsis* spp. Tandem mass spectrometry molecular networks were created using the GNPS platform (http://gnps.ucsd.edu). Circles represent unknown metabolites and hexagon-shaped nodes represent known compounds from natural product databases. Mte: macrotermycin, slf: saalfelduracin, PEs: phosphoethanolamines, rif: rifamycins. The dataset was uploaded to the MAssIVE server with the accession code MSV000093302 [https://massive.ucsd.edu/ProteoSAFe/dataset.jsp?task=7c1ba2e849ed42f88592c69f6a599d83].

Analysis of metabolite production on minimal media vs. complex media.

Amycolatopsis sp. PS_44_ISF1 pre-cultures (50 ml) were prepared in ISP2 broth and cultivated for seven days at 30 °C and shaking at 150 rpm. Agar plates with hree different minimal media (CSA, MM, SMMS) and three complex media (ISP2, PDA, MS) were inoculated with 100 µl of the pre-culture and further incubated at 30 °C for seven days (for media recipes, see Supplementary Table 2). Afterwards, three biological replicates of each medium were extracted. Agar was cut into small pieces (0.5 cm x 0.5 cm) and transferred into a pre-weighed 100 mL beaker. After determining the weight of the transferred agar, it was extracted with 40 mL EtOAc overnight at room temperature. The solvent was then filtered off and evaporated *in vacuo*. The resulting extract was dissolved in 100% MeOH to a final concentration of 50 µg/ml and subjected to HR-UPLC-ESI-MS analysis. The production of each metabolite of interest was evaluated by the comparison of the peak area using the extracted ion mode (EIC) and normalized to the wet weight of agar extracted (technical replicate: n=1; peak area/mg wet weight).

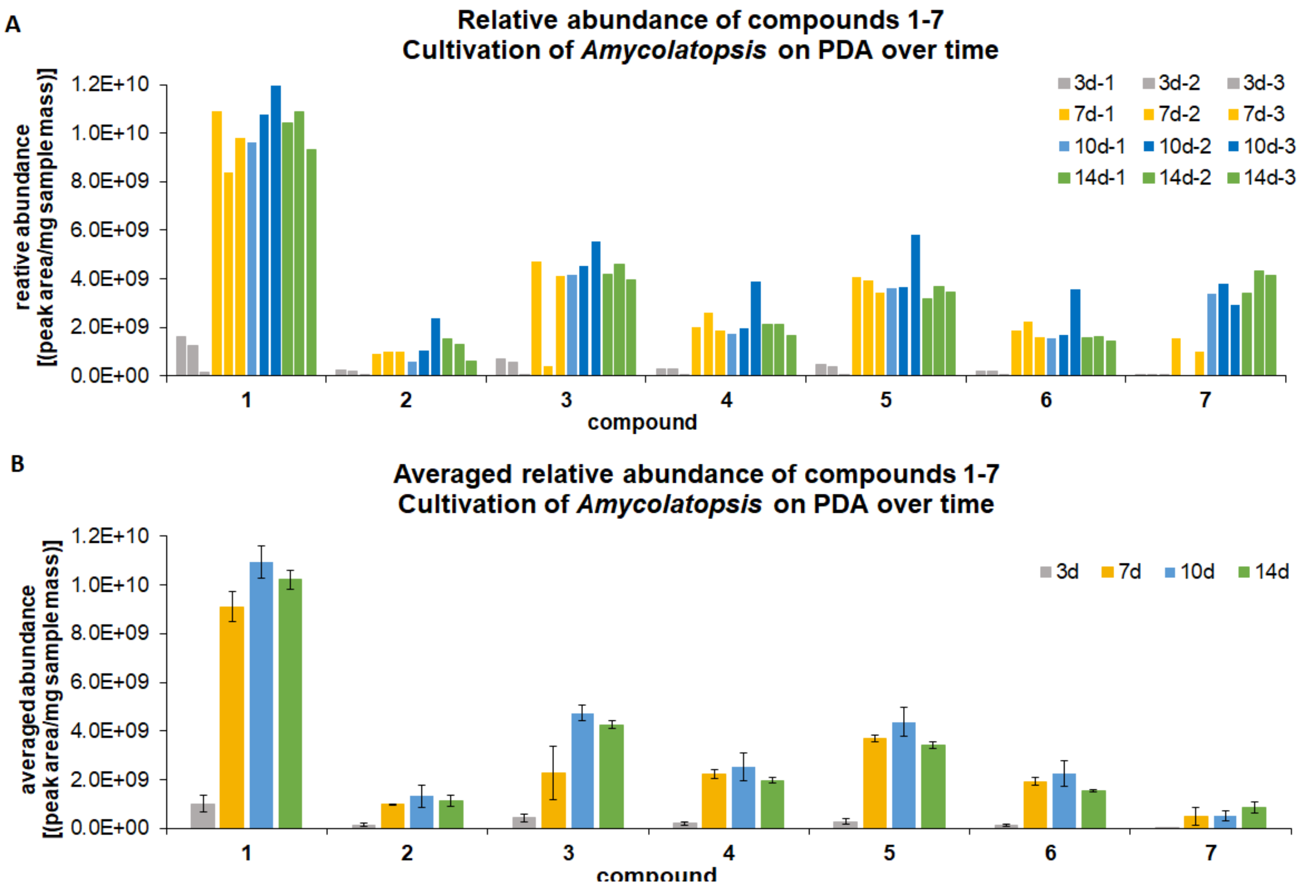

Supplementary Figure 22. Media-dependent abundance of compound 1-7 after cultivation on PDA over a cultivation period of 7 days. A) Relative abundance of compounds **1-7** in plate cultures of *Amycolatopsis* sp. PS_44_ISF1 and extraction with MeOH (data of each biological replicate is shown as single data point, peak area/mg wet weight); and B) averaged abundance (peak area ± standard error, n = 3).
Abundance was calculated from peak areas extracted from EICs ($[M+H]^+$ ± 5 ppm) for **1** (731.4662), **2** (745.4818), **3** (759.4975), **4** (773.5131), **5** (787.5288), **6** (801.5444), and **7** (694.3519), and normalized to the weight of the extracted agar. Source data are available in the Source Data file.

Analysis of metabolite production on PDA at different time points

Amycolatopsis sp. PS_44_ISF1 pre-cultures (50 ml) were prepared in ISP2 broth and cultivated for seven days at 30 °C and shaking at 150 rpm. PDA agar plates were inoculated with 100 µl of the pre-culture and further incubated at 30 °C for a period of 14 days (for media recipes, see Supplementary Table 2). Three biological replicates (1-3) of each medium were extracted after 3, 7, 10 and 14 days of incubation. Agar was cut into small pieces (0.5 cm x 0.5 cm) and transferred into a pre-weighed 100 mL beaker. After determining the weight of the transferred agar, it was extracted with 40 mL MeOH overnight at room temperature. The solvent was then filtered off and evaporated *in vacuo*. The resulting residue was dissolved in 100% MeOH to a final concentration of 50 µg/ml and subjected to HR-UPLC-ESI-MS analysis. The production of each metabolite of interest was evaluated by the comparison of the peak area using the extracted ion mode (EIC) and normalized to the wet weight of agar extracted (technical replicate: n=1; peak area/mg wet weight).

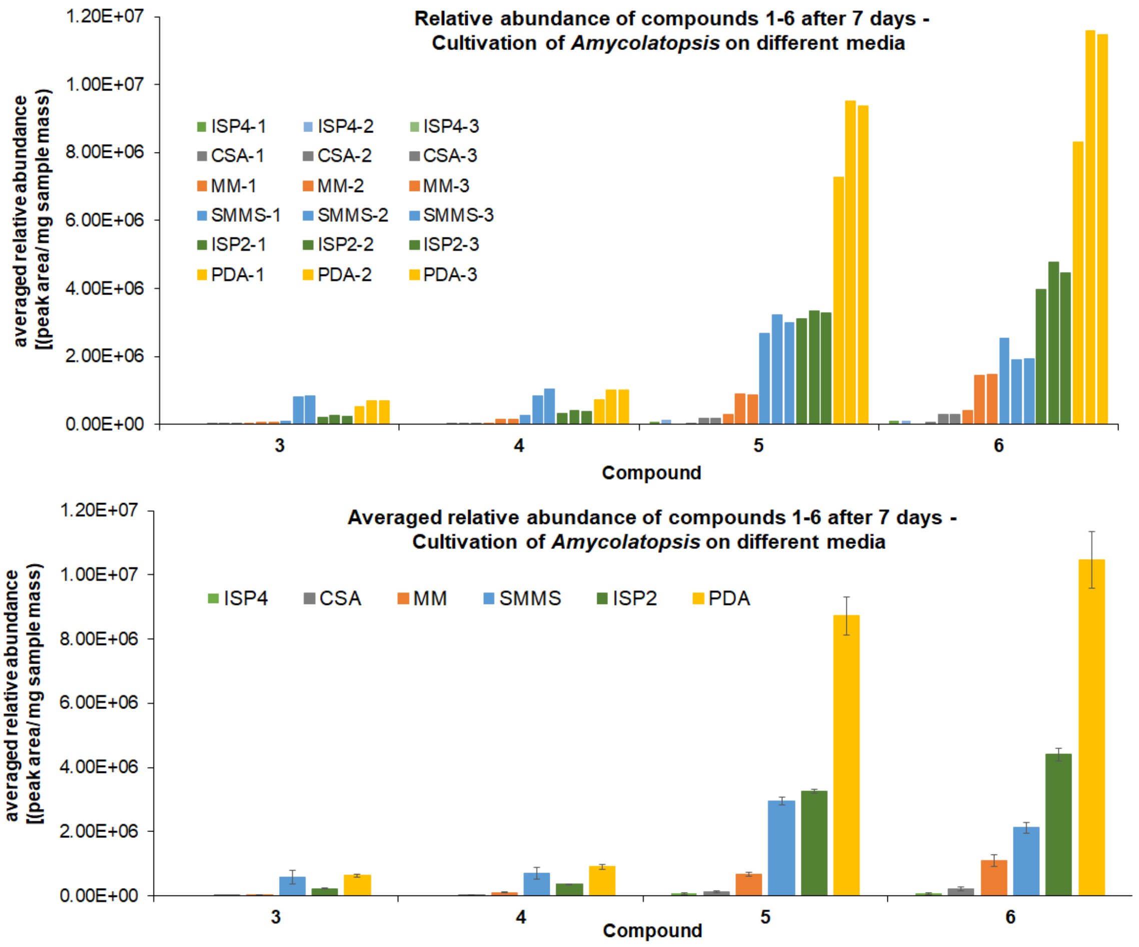

Supplementary Figure 23. Time-dependent abundance of compound 1-7 after cultivation on PDA over a cultivation period of 14 days. A) Relative abundance of compounds **1-7** in plate cultures of *Amycolatopsis* sp. PS_44_ISF1 after 3, 7, 10 and 14 days of incubation and extraction with MeOH (data of each biological replicate is shown as single data point, peak area/mg wet weight); and B) averaged abundance (peak area ± standard error, n = 3).
Abundance was calculated from peak areas extracted from EICs ($[M+H]^+$ ± 5 ppm) for **1** (731.4662), **2** (745.4818), **3** (759.4975), **4** (773.5131), **5** (787.5288), **6** (801.5444), and **7** (694.3519), and normalized to the weight of the extracted agar. Source data are available in the Source Data file.

Isolation and characterization of metabolites

For metabolite extraction from agar plates, 500 µl 7-day-old pre-cultures of *Amycolatopsis* spp. were streaked on 120 PDA (peptides) agar plates (90x16 mm). There cultures were sealed with parafilm and incubated at 30 °C for another 7 days. Plates were cut in small cubes (1x1 cm) and extracted with 6 L of methanol overnight. Afterwards, the solvent was filtered off and evaporated *in vacuo*. The dried extract was then redissolved in 20% methanol and subjected to solid phase extraction (SPE). A 10 g C_{18} column was activated with 2 column volumes (CV) of 100% methanol and equilibrated with 20% methanol. Afterwards the extract was loaded, washed with 20% methanol, and eluted with 50% and 100% methanol (2 CV). All three fractions were concentrated *in vacuo*. For peptide isolation, the combined 50% and 100% fractions were used for further processing.

Isolation of pachycephalamides A-F (1-6). A portion of the crude fraction (98.5 g) of was resuspended with Celite in MeOH and dried *in vacuo* to generate Celite-adsorbed extract in a round flask. The Celite-adsorbed extract was loaded onto 20 g of the prepacked C18 SPE Sepak resin. The extract was fractionated by elution with a step gradient composed of water and methanol (10%, 20%, 40%, 60%, 80%, and 100%). Pachycephalamides A-F eluted in the 60% and 80% methanol SPE fractions based on an LC-MS analysis. The entire SPE fractionation procedure was repeated five times. To obtain pure pachycephalamides A-F (**1**-**6**), the materials from the 60% and 80% methanol SPE fractions were purified by semipreparative reversed-phase HPLC (Phenomenex Luna C18(2) 250 x 10 mm column, particle size 5 µm, pore diameter 100 Å, flow rate: 2 mL/min, detection: UV 210 nm, gradient solvent system: 10−40% aqueous acetonitrile over 30 min). Pachycephalamides A-F (**1**-**6**) eluted at 23.1, 24.2, 25.3, 26.3, 27.2, and 28.6 min, respectively.

Characterization of pachycephalamide A-F (1-6). Pachycephalamide A (**1**) was isolated as a white powder with a molecular formula of $C_{33}H_{62}O_{10}N_8$ determined by HR-ESI-MS (calcd. $[M + H]^+$ *m/z* 731.4662, obsd. $[M + H]^+$ *m/z* 731.4653) requiring seven degrees of unsaturation. The ^1H and ^{13}C NMR spectra of **1** measured in CD_3OH-d_3 showed well dispersed proton and carbon resonances indicating a pattern of chemical shifts for a typical peptidic compound as bearing 5 amide/acid carbonyl signals (δ_C 175.4, 173.9, 173.8, 173.7, and 173.3), 5 α-carbon signals (δ_C 61.2, 61.1, 53.2, 51.7, and 51.3), and 5 α-proton signals (δ_H 4.26, 4.16, 4.12, 3.96, and 3.84) (Supplementary Table 6, Supplementary Table 7). In addition to these typical peptidic resonances, pachycephalamide A showed PKS carbon and proton signal patterns including three hydroxyl carbon signals [δ_C 71.8, 69.8, and 65.9]. Further analysis of 1D (^1H and ^{13}C) and 2D (COSY, HSQC, and HMBC) NMR data in CD_3OH-d_3 enabled the identification of five amino acid residues (a leucine, an alanine, an arginine, and two valines) and one trihydroxyoctanoic acid (Supplementary Figure 26, Supplementary Figure 27, Supplementary Figure 28). First, the leucine residue at C-terminal end [δ_C 178.1] was assigned based on COSY and HMBC correlations. The leucine (Leu) residue showed the COSY correlations between the amide NH [δ_H 7.77] of Leu and α-proton [δ_H 3.84], α-proton and β-protons [δ_H 1.65, 1.31], β-protons and γ-proton [δ_H 1.66], and γ-proton and six methyl protons [δ_H 0.93, 3H and 0.89, 3H]. This connectivity's were supported by HMBC correlations (Supplementary Figure 28). The amide NH [δ_H 7.77] of Leu correlated with the carbonyl carbon [δ_C 173.3] of Valine-1 (Val-1) in the HMBC spectrum. The Val-1 residue was deduced by ^1H-^1H correlations between NH [δ_H 8.00] of Val-1 and α-proton [δ_H 4.12], α-proton and β-proton [δ_H 2.10], and β-proton and 2 methyl groups [δ_H 0.99, 6H]. The amide proton [δ_H 8.00] showed the HMBC correlation to α-carbon [δ_C 61.2] of Val-1 and the β-proton established the connection to α-carbon and the methyl group carbon [δ_C 18.9]. The ^1H-^{13}C long-range couplings from the amide NH [δ_H 8.00] of Val-1 and the α-proton [δ_H 4.16] of valine-2 (Val-2) to the carbonyl carbon [δ_C 173.8] of Val-2 allowed for the connectivity from Val-1 to Val-2. The valine-2 residue showed analogous COSY and HMBC correlation patterns as Valine-1 including α-, β- carbons and protons

[δ_C 61.1; δ_H 4.16, δ_C 31.4; δ_H 2.12], and two methyl groups [δ_C 19.1, 2C; δ_H 0.99, 6H]. The strong long-range heteronuclear couplings from the amide proton of Val-2 and α-proton [δ_H 3.96] of Arginine (Arg) to the carbonyl carbon of [δ_C 173.9] Arg established the sequence from Val-2 to Arg. The 3-carbon aliphatic straight chain of Arg was deduced based on COSY and HMBC correlations and the protons at the delta position [δ_H 3.15, 2H] showed the HMBC correlation to the carbon [δ_C 158.9] in the guanidine moiety. The extension of the chain between arginine and alanine (Ala) was established by the ^{1}H-^{13}C long-range couplings from NH [δ_H 7.65] of Arg and the α-proton [δ_H 4.26] of Alanine (Ala) to the amide carbon [δ_C 175.4] of Ala. The NH proton [δ_H 8.19] and the methyl group [δ_C 18.1; δ_H 1.38] connected to the α-carbon [δ_C 51.3] of the alanine residue was connected based on COSY and HMBC correlations. Lastly, the COSY and HMBC correlations from the methyl end [δ_C 23.7; δ_H 1.22] of the fatty chain to the methylene position at C-27 [δ_C 41.4; δ_H 2.38, 2H] in the trihydroxyoctanoic acid (THOA) was established as the last residue in **1**. The connectivity between Ala and THOA was deduced by HMBC correlations from H-24, 27, and 28 [δ_H 4.26, 2.38, and 4.06] to C-26 [δ_C 173.6] satisfying the 7 degrees of unsaturation for Pachycephalamide A (**1**).

Pachycephalamide B (**2**) was purified as an amorphous white powder with a molecular formula of $C_{34}H_{64}O_{10}N_8$ determined by HR-ESI-MS (calcd. $[M + H]^+$ *m/z* 745.4829, obsd. $[M + H]^+$ *m/z* 745.4802). The 1D and 2D NMR spectra showed analogous PKS-NRPS hybrid features compared to those of **1** and the difference was that the molecular formula of **2** possesses one more methylene group at the trihydroxyoctanoic acid in **1** (Supplementary Figure 29-Supplementary Figure 32).

Pachycephalamide C (**3**) was purified as an amorphous white powder along with pachycephalamides A and B. Its molecular formula of $C_{35}H_{66}O_{10}N_8$ was determined by HR-ESI-MS (calcd. $[M + H]^+$ *m/z* 759.4985, obsd. $[M + H]^+$ *m/z* 759.4968). The 1D and 2D NMR spectra of **3** showed similar features compared to those of **2** and the only difference was that the molecular formula of **3** bears one more methylene group at the trihydroxynonanoic acid in pachycephalamide B (**2**) (Supplementary Figure 33-Supplementary Figure 37).

Pachycephalamide D (**4**) was isolated as an amorphous white powder with a molecular formula of $C_{36}H_{68}O_{10}N_8$ was determined by HR-ESI-MS (calcd. $[M + H]^+$ *m/z* 773.5131, obsd. $[M + H]^+$ *m/z* 773.5112). The 1D and 2D NMR data of **4** showed similar features compared to those of **1**-**3** and the chemical structural difference was that the molecular formula of **4** possesses one more CH_2 group at the trihydroxydecanoic acid in pachycephalamide C (**3**) (Supplementary Figure 38-Supplementary Figure 42).

Pachycephalamide E (**5**) was purified as an amorphous white powder with a molecular formula of $C_{37}H_{70}O_{10}N_8$ was determined by HR-ESI-MS (calcd. $[M + H]^+$ *m/z* 787.5298, obsd. $[M + H]^+$ *m/z* 787.5278). Based on the 1D and 2D NMR data of **5**, pachycephalamide E revealed that it has analogous PKS-NRPS hybrid features compared to those of **1**-**4** and the structural difference was that the molecular formula of **5** possesses one more methylene group at the trihydroxyundecadoic acid in pachycephalamide D (**4**) (Supplementary Figure 43-Supplementary Figure 47).

Pachycephalamide F (**6**) was isolated as an amorphous white powder with a molecular formula of $C_{38}H_{72}O_{10}N_8$ was determined by HR-ESI-MS (calcd. $[M + H]^+$ *m/z* 801.5444, obsd. $[M + H]^+$ *m/z* 801.5424). An analysis of 1D and 2D NMR data for pachycephalamide F (**6**) showed that **6** have very similar PKS-NRPS hybrid features compared to

those of **1**-**5** and the only structural difference was that the molecular formula of **6** possesses one more methylene group at the acid chain bearing trihydroxytridecanoic acid instead of trihydroxydodecanoic acid in pachycephalamide E (**5**) (Supplementary Figure 48-Supplementary Figure 52).

Isolation of demiguisin (7). Using liquid chromatography-mass spectrometry (LC-MS), the desiccated crude extract was dissolved in methanol at a concentration of 250 g/mL. Using an Agilent 1290 ultra-high performance liquid-chromatography (UHPLC) system (Agilent Technologies, Santa Clara, USA) equipped with a 1290 Infinity binary pump and a YMC-triart C18 column (150 x 20 mm, 1.9 µm; YMC KOREA Co., Seongnam, Korea), high-performance liquid chromatography (HPLC) measurements were conducted. The mobile phases consisted of 0.1% formic acid in water (A) and 0.1% formic acid in acetonitrile (B) with the following gradients: 13% B (0–5 min), 13%–18% B (5–18 min), 18%–100% B (18–30 min), 100% B (30–35 min) and 13% B (35–40 min) with an injection volume of 100 µL in eighteen replicates. Compound **7** eluted at 17.1 min. Chemicals: Methanol (VWR, Germany); water for analytical and semi-preparative HPLC (Millipore, Germany), formic acid (Carl Roth, Germany); acetonitrile (VWR as LC-MS grade).

Supplementary Table 6. ^{13}C NMR data for pachycephalamides A-F (**1-6**) in CD_3OH-*d*$_3$ at 150 MHz.

			**1**	**2**	**3**	**4**	**5**	**6**
unit	position	type			δ_C			
Leucine	1	C	178.1	178.1	178.0	178.0	178.0	178.9
	2	CH	53.2	53.7	52.7	53.9	53.5	53.7
	3	CH_2	41.3	40.0	41.5	40.1	41.3	40.3
	4	CH	25.5	25.8	25.8	25.6	25.3	25.5
	5	CH_3	23.9	23.9	23.7	22.7	22.7	22.9
	6	CH_3	21.7	21.9	21.9	21.9	21.9	22.0
Valine-1	7	C	173.3	173.3	173.6	173.2	173.6	173.2
	8	CH	61.2	61.1	60.0	61.2	61.2	61.1
	9	CH	31.2	31.1	30.0	30.1	31.2	31.1
	10	CH_3	18.9	18.8	18.8	18.8	19.0	18.9
	11	CH_3	18.9	18.8	18.8	18.8	19.0	18.9
Valine-2	12	C	173.8	173.8	173.9	173.0	173.8	173.8
	13	CH	61.1	61.0	61.0	61.1	61.3	61.1
	14	CH	31.4	31.1	31.0	30.0	31.1	31.0
	15	CH_3	19.1	19.0	18.7	18.6	18.9	18.7
	16	CH_3	19.1	19.0	18.7	18.6	18.9	18.7
Arginine	17	C	173.9	174.0	174.2	173.6	174.1	174.1
	18	CH	51.7	51.7	51.5	51.8	52.7	51.7
	19	CH_2	29.1	29.2	29.2	28.9	29.1	29.0
	20	CH_2	26.1	25.8	25.7	25.9	25.8	25.9
	21	CH_2	42.2	41.9	41.8	41.5	41.7	41.7
	22	C	158.9	158.9	158.4	158.6	158.6	158.7
Alanine	23	C	175.4	175.0	175.2	174.3	175.1	174.8
	24	CH	51.3	51.1	50.6	50.5	51.4	51.3
	25	CH_3	18.1	17.9	17.8	17.0	17.9	18.0
Acid	26	C	173.7	173.3	173.2	172.3	173.1	173.3
	27	CH_2	41.4	41.4	41.3	40.6	41.4	41.3
	28	CH	69.8	69.9	70.0	69.9	71.6	71.4
	29	CH_2	41.6	41.8	42.1	41.8	44.0	43.4
	30	CH	71.8	70.0	69.4	70.5	70.5	71.3
	31	CH_2	45.8	40.7	40.0	40.4	47.3	43.7
	32	CH	65.9	71.2	72.0	72.2	67.9	69.9
	33	CH_n	23.7, CH_3	28.3, CH_2	26.1, CH_2	38.4, CH_2	37.3, CH_2	41.3 CH_2
	34	CH_n		18.8, CH_3	19.5, CH_2	39.8, CH_2	25.2, CH_2	27.2, CH_2
	35	CH_n			14.5, CH_3	28.0, CH_2	35.8, CH_2	29.2, CH_2
	36	CH_n				14.7, CH_3	28.9, CH_2	32.8, CH_2
	37	CH_n					14.5, CH_3	22.1, CH_2
	38	CH_n						16.5, CH_3

Supplementary Table 7. ^{1}H NMR data for pachycephalamides A-F (**1-6**) in CD_3OH-*d*$_3$ at 600 MHz.

unit	position	type	**1**	**2**[a]	**3**	**4**	**5**[a]	**6**
			δ_H (*J* in Hz)					
Leucine	2	CH	3.84, m	3.84, m	3.86, m	3.85, m	3.82, m	3.86, m
	3	CH_2	1.65, m	1.64, m	1.64, m	1.64, m	1.64, m	1.65, m
			1.31, m	1.31, m	1.33, m	1.32, m	1.33, m	1.32, m
	4	CH	1.66, m	1.66, m	1.62, m	1.64, m	1.64, m	1.66, m
	5	CH_3	0.93, d (6.5)	0.92, d (6.5)	0.92, d (7.0)	0.92, d (7.0)	0.91, d (7.0)	0.91, d (7.0)
	6	CH_3	0.89, d (6.5)	0.88, d (6.5)	0.89, d (7.0)	0.89, d (7.0)	0.87, d (7.0)	0.87, d (7.0)
		2-NH	7.77, d (8.0)		7.75, d (9.0)	7.75, d (9.0)		7.75, d (9.0)
Valine-1	8	CH	4.12, m	4.12, m	4.14, dd (8.0, 8.0)	4.14, dd (8.0, 8.0)	4.15, dd (7.5, 7.5)	4.14, dd (8.0, 8.0)
	9	CH	2.10, m	2.10, m	2.12, m	2.12, m	2.12, m	2.12, m
	10	CH_3	0.99, d (6.0)	1.01, d (6.0)	1.01, m	1.01, m	1.01, m	1.01, m
	11	CH_3	0.99, d (6.0)	1.01, d (6.0)	1.01, m	1.01, m	1.01, m	1.01, m
		8-NH	8.00, d (8.0)		7.98, d (8.0)	7.99, d (8.0)		7.98, d (8.0)
Valine-2	13	CH	4.16, m	4.16, dd (8.0, 8.0)	4.18, dd (8.0, 8.0)	4.17, m	4.18, dd (8.0, 8.0)	4.18, m
	14	CH	2.12, m	2.11, m	2.12, m	2.11, m	2.11, m	2.11, m
	15	CH_3	0.99, d (6.0)	0.99, d (6.0)	0.99, m	1.00, m	0.99, m	0.99, m
	16	CH_3	0.99, d (6.0)	0.99, d (6.0)	0.99, m	1.00, m	0.99, m	0.99, m
		13-NH	8.13, d (7.0)		8.09, d (8.0)	8.09, d (8.0)		8.09, d (8.0)
Arginine	18	CH	3.96, m	3.97, m	3.98, m	3.98, m	3.98, m	3.98, m
	19	CH_2	1.67, m	1.66, m	1.66, m	1.66, m	1.66, m	1.66, m
	20	CH_2	1.61, m	1.61, m	1.64, m	1.64, m	1.64, m	1.64, m
	21	CH_2	3.15, m	3.15, m	3.16, m	3.15, m	3.15, m	3.15, m
		18-NH	7.65, d (9.0)		7.65, d (9.0)	7.65, d (9.0)		7.65, d (9.0)
		21-NH	7.62, br. s		7.70, br. s	7.70, br. s		7.70, br. s
Alanine	24	CH	4.26, qd (7.0, 7.0)	4.25, qd (7.0, 7.0)	4.26, qd (6.5, 6.5)	4.26, qd (6.5, 6.5)	4.26, qd (6.5, 6.5)	4.26, qd (6.5, 6.5)
	25	CH_3	1.38, d (7.0)	1.38, d (7.0)	1.39, d (6.5)	1.38, d (6.5)	1.38, d (6.5)	1.38, d (6.5)
		24-NH	8.19, d (7.0)		8.19, d (6.5)	8.19, d (6.5)		8.19, d (6.5)
Acid	27	CH_2	2.38, m	2.36, m	2.35, m	2.35, m	2.35, m	2.35, m
	28	CH	4.06, dd (7.5, 7.0)	4.10, m	4.08, dd (7.5, 7.5)	4.08, dd (7.5, 7.5)	4.08, dd (7.5, 7.5)	4.08, dd (7.5, 7.5)
	29	CH_2	2.34, m	2.36, m	2.38, m	2.38, m	2.38, m	2.38, m
	30	CH	4.00, m	4.01, m	4.00, m	4.00, m	4.00, m	4.00, m
	31	CH_2	2.44, m	2.46, m	2.48, m	2.48, m	2.48, m	2.48, m
	32	CH	4.17, m	4.18, m	3.97, m	4.01, m	3.99, m	3.98, m
	33	CH_n	1.22, d (6.0), CH_3	1.61, m, CH_2	1.60, m, CH_2	1.74, m, CH_2	1.78, m, CH_2	1.73, m, CH_2
	34	CH_n		0.98, d (6.0), CH_3	1.68, m, CH_2	1.61, m, CH_2	1.58, m, CH_2	1.78, m, CH_2
	35	CH_n			0.96, m, CH_3	1.43, m, CH_2	1.72, m, CH_2	1.72, m, CH_2
						1.21, m, CH_2	1.46, m, CH_2	1.66, m, CH_2
	36	CH_n				0.92, m, CH_3	1.41, m, CH_2	1.43, m, CH_2
							1.23, m, CH_2	1.24, m, CH_2
	37	CH_n					0.90, d (6.0), CH_3	1.28, m, CH_2
								1.21, m, CH_2
	38	CH_3						0.90, d (6.0), CH_3

[a] measured in CD_3OD

Supplementary Table 8. ^{1}H and ^{13}C NMR data for demiguisin (7) in DMSO-d_6 at 700 (150) MHz.

	No.	type	δ_C[a]	δ_H[b]	Mult (*J* in Hz)
**Gly**	1	C	171.7		
	2	CH_2	42.2	3.59	dd (16.9, 5.8)
				3.46	m
		2-NH		7.82	s
**β,γ-OH-Piz**	3	C	168.4		
	4	CH	56.6	4.76	s
	5	CH	68.4	4.17	s
	6	CH	65.4	3.39	m
	7	CH_2	48.6	3.15	br. t (12.9, 12.9)
				2.59	m
		7-NH		3.17	br. t (12.9, 12.9)
**Leu**	8	C	174.6		
	9	CH	47.6	5.31	m
	10	CH_2	40.8	1.59	m
				1.36	m
	11	CH	24.8	1.58	m
	12	CH_3	23.8	0.88	m
	13	CH_3	22.7	0.87	m
		9-NH		7.93	d (8.83)
**Piz**	14	C	171.1		
	15	CH	50.4	4.95	dd (5.9, 1.9)
	16	CH_2	26.3	2.08	m
				1.78	m
	17	CH_2	21.4	1.53	m
				1.46	m
	18	CH_2	47.2	2.98	d (11.4)
				2.71	m
		18-NH		5.12	m
**DHPA**	19	C	170.5		
	20	CH	51.9	5.75	m
	21	CH	122.6	5.88	dt (10.2, 2.8, 2.8)
	22	CH	126.9	5.93	m
	23	CH_2	24.8	2.20	m
				2.05	m
	24	CH_2	40.3	3.96	m
				3.38	m
**γ-c-Pro**	25	C	172.1		
	26	CH	59.1	3.98	m
	27	CH_2	34.5	2.33	dt (12.4, 7.6, 7.6)
				1.67	dt (12.5, 9.1, 9.1)
	28	CH	44.3	2.84	m
	29	CH_2	50.6	3.04	m
				2.90	dd (10.5, 8.2)
	30	C	175.7		
		30-NH_2		7.34	br. s
				6.82	br. s

Measured in DMSO-d_6, [a]150 MHz, [b]700 MHz.

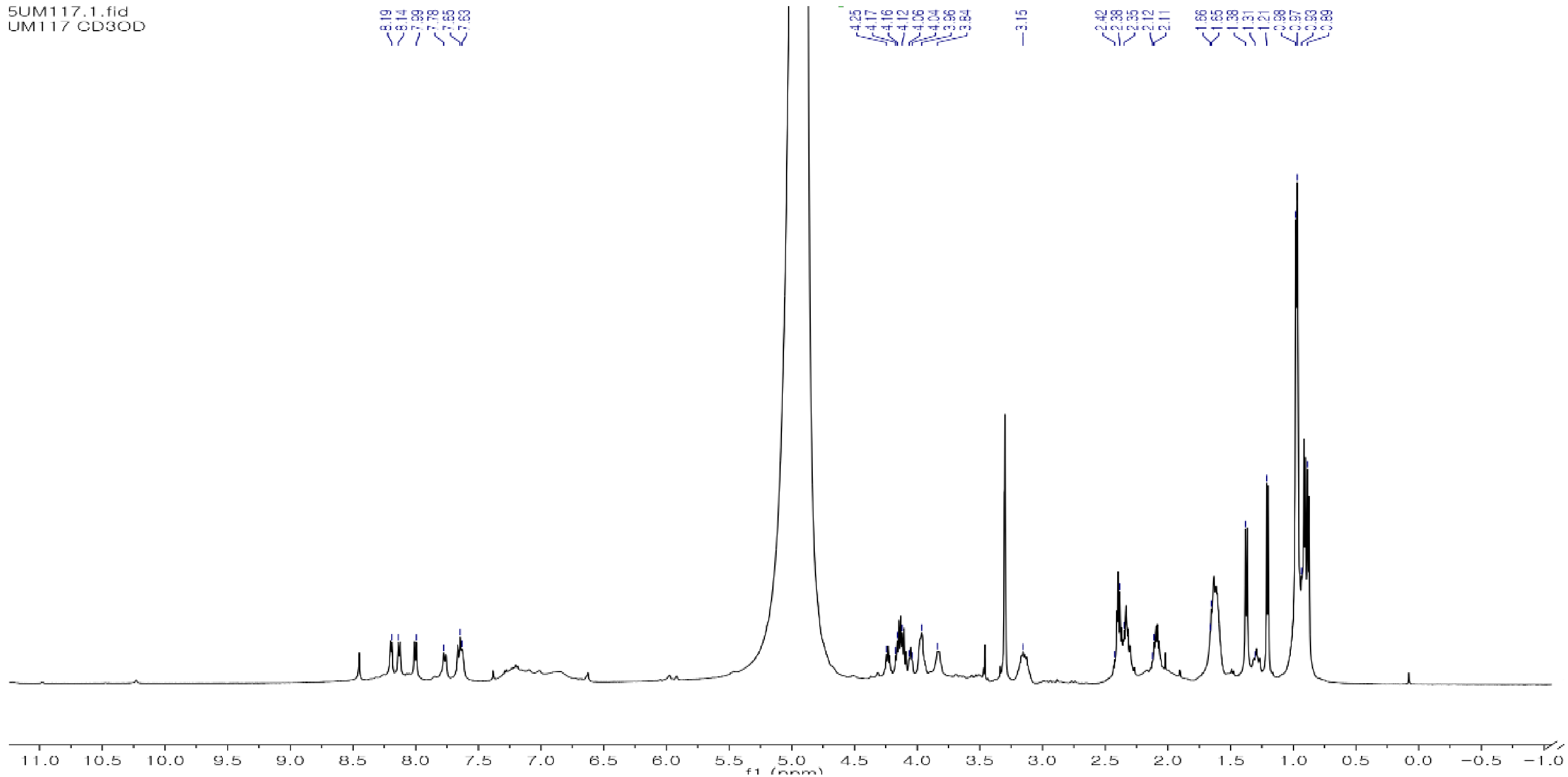

Supplementary Figure 24. ^{1}H NMR spectrum of pachycephalamide A (**1**) in CD_3OH-*d*$_3$.

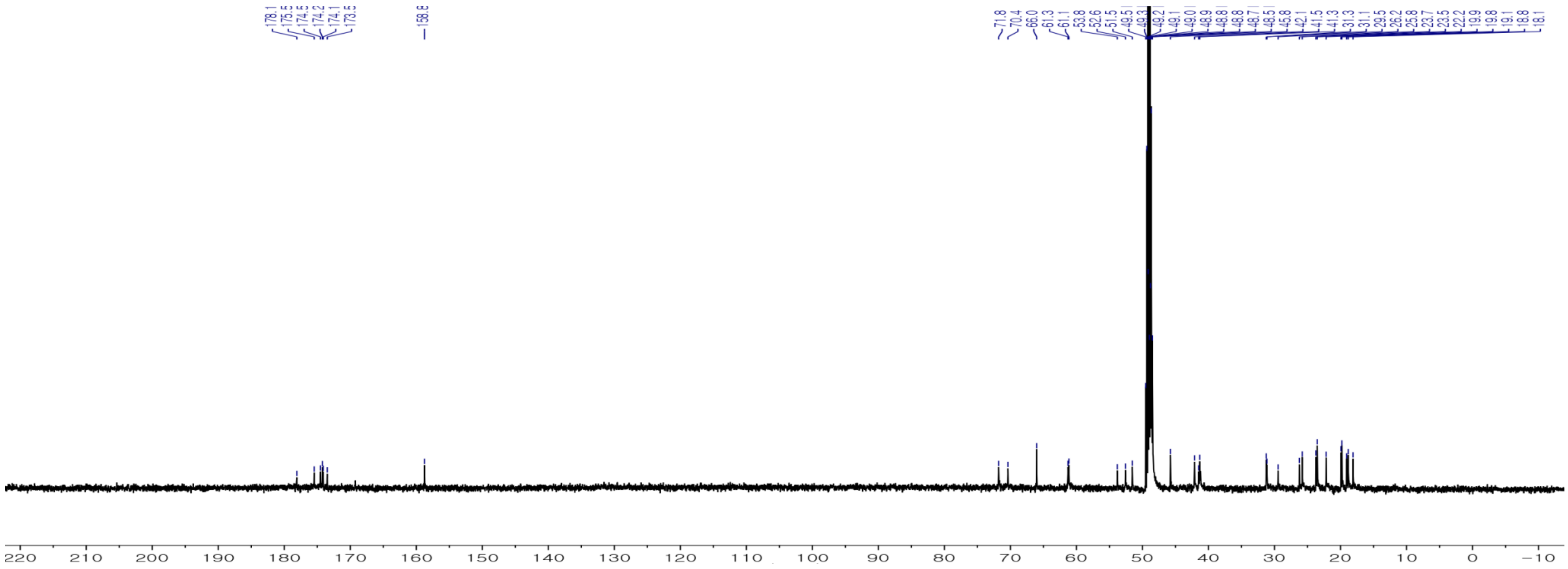

Supplementary Figure 25. ^{13}C NMR spectrum of pachycephalamide A (**1**) in CD_3OH-d_3.

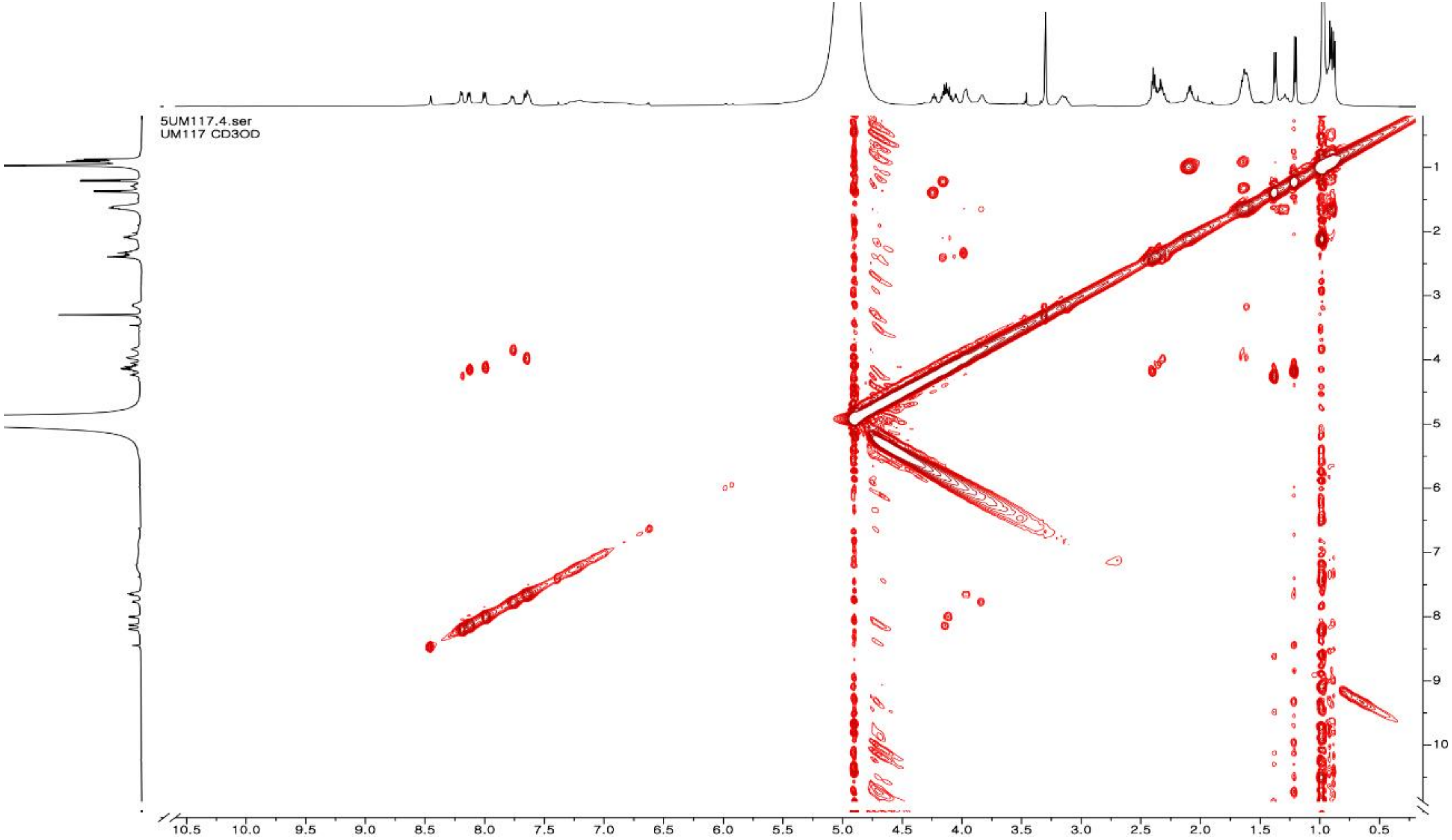

Supplementary Figure 26. COSY NMR spectrum of pachycephalamide A (**1**) in CD_3OH-d_3.

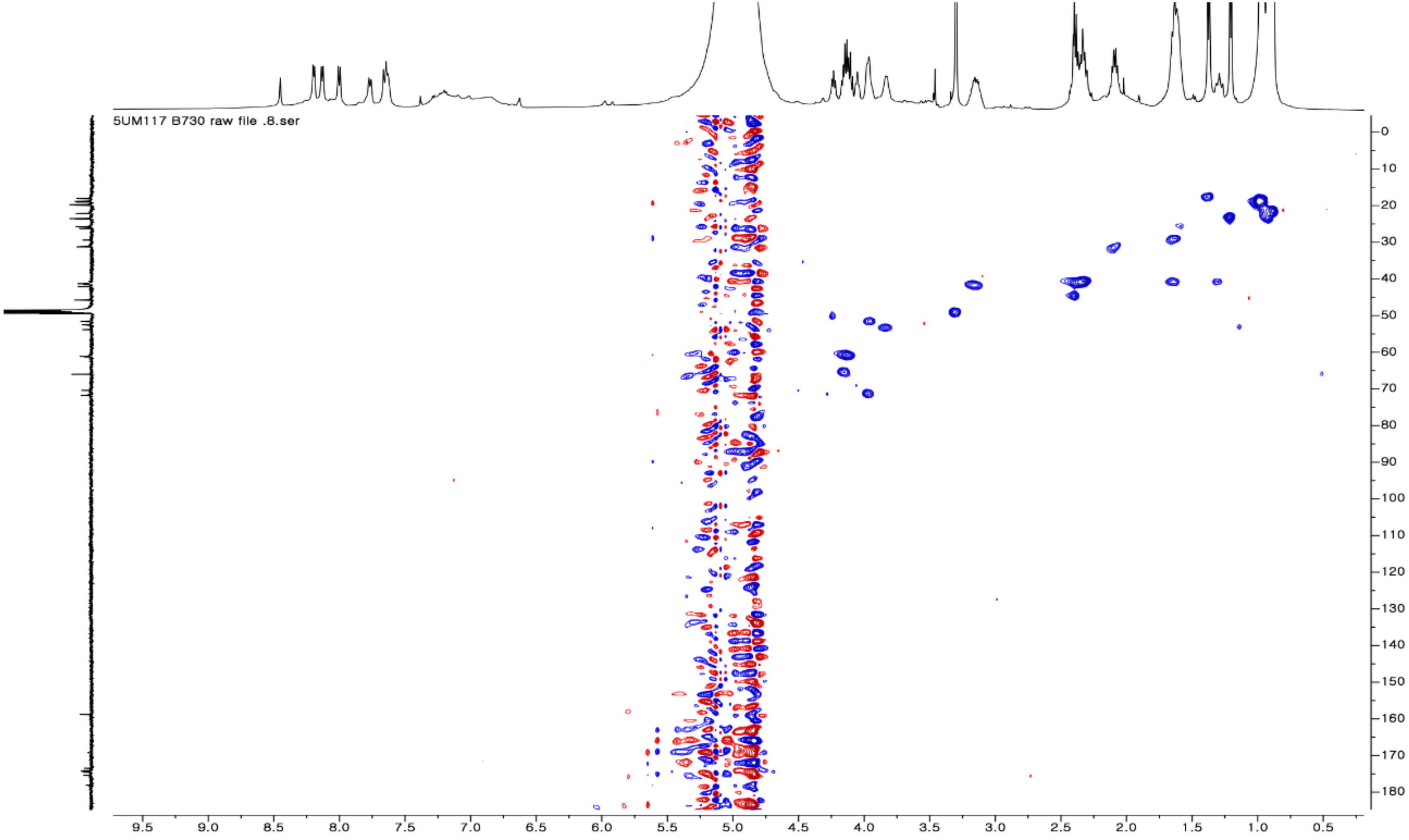

Supplementary Figure 27. HSQC NMR spectrum of pachycephalamide A (**1**) in CD_3OH-d_3.

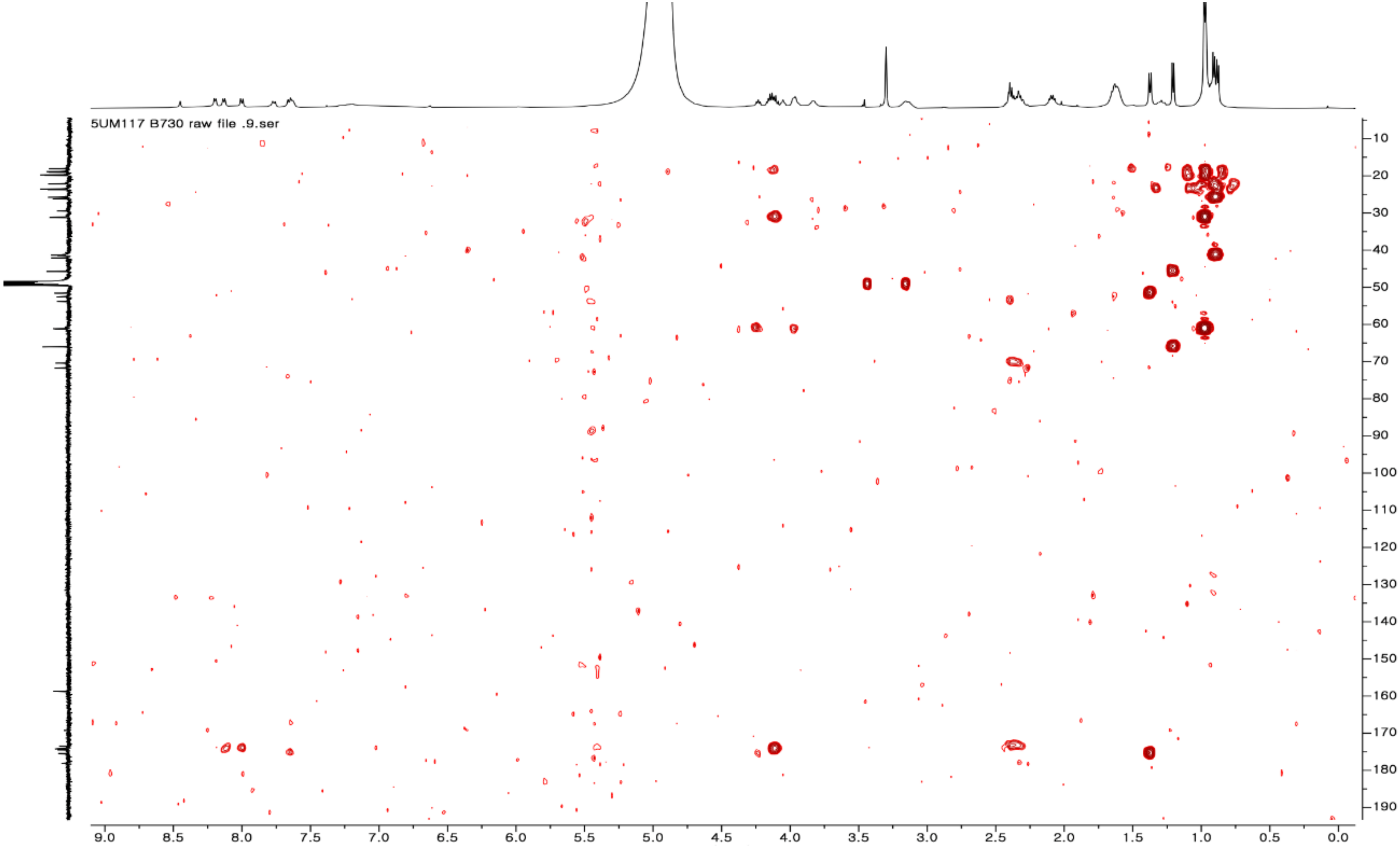

Supplementary Figure 28. HMBC NMR spectrum of pachycephalamide A (**1**) in CD_3OH-d_3.

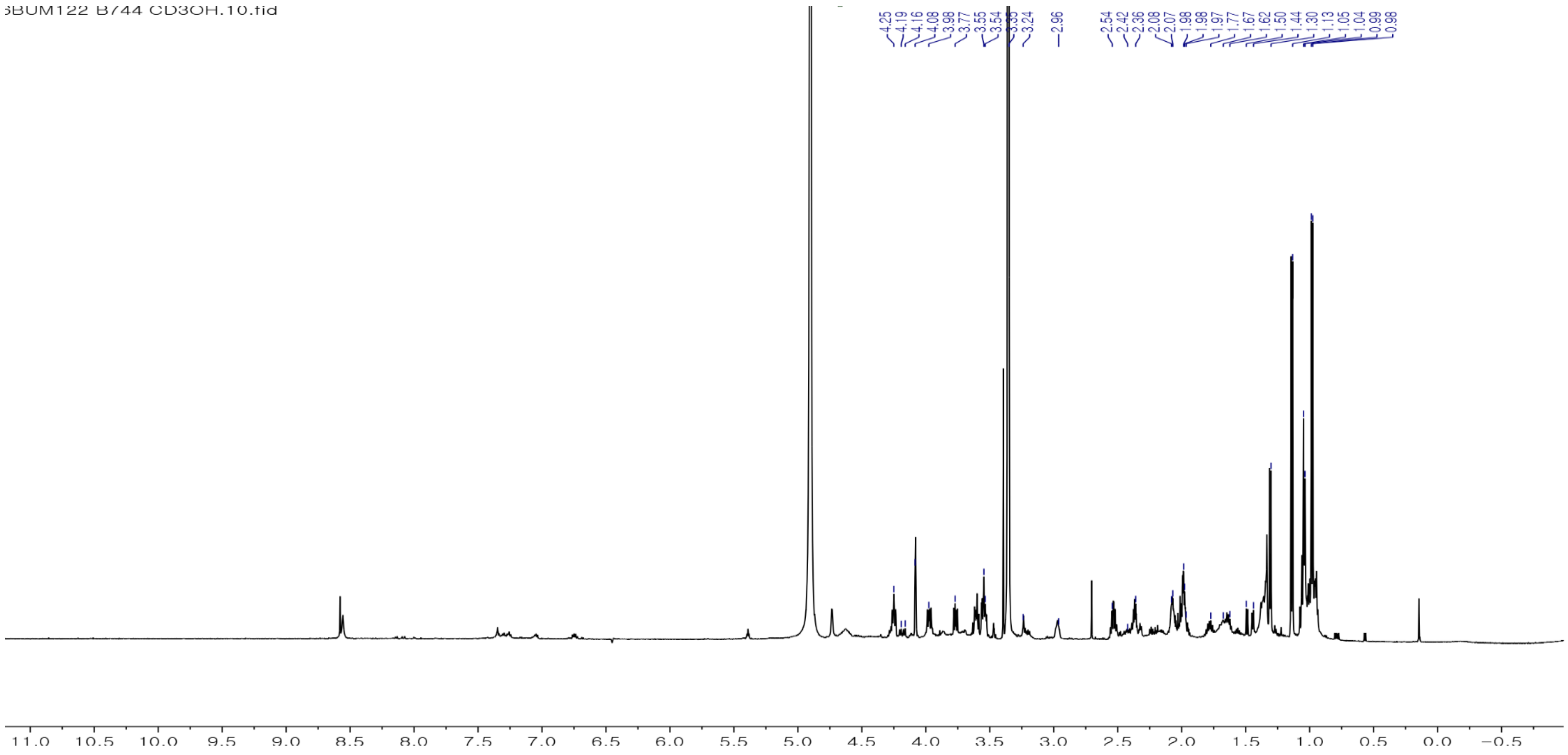

Supplementary Figure 29. ^{1}H NMR spectrum of pachycephalamide B (**2**) in CD_3OD-*d*$_4$.

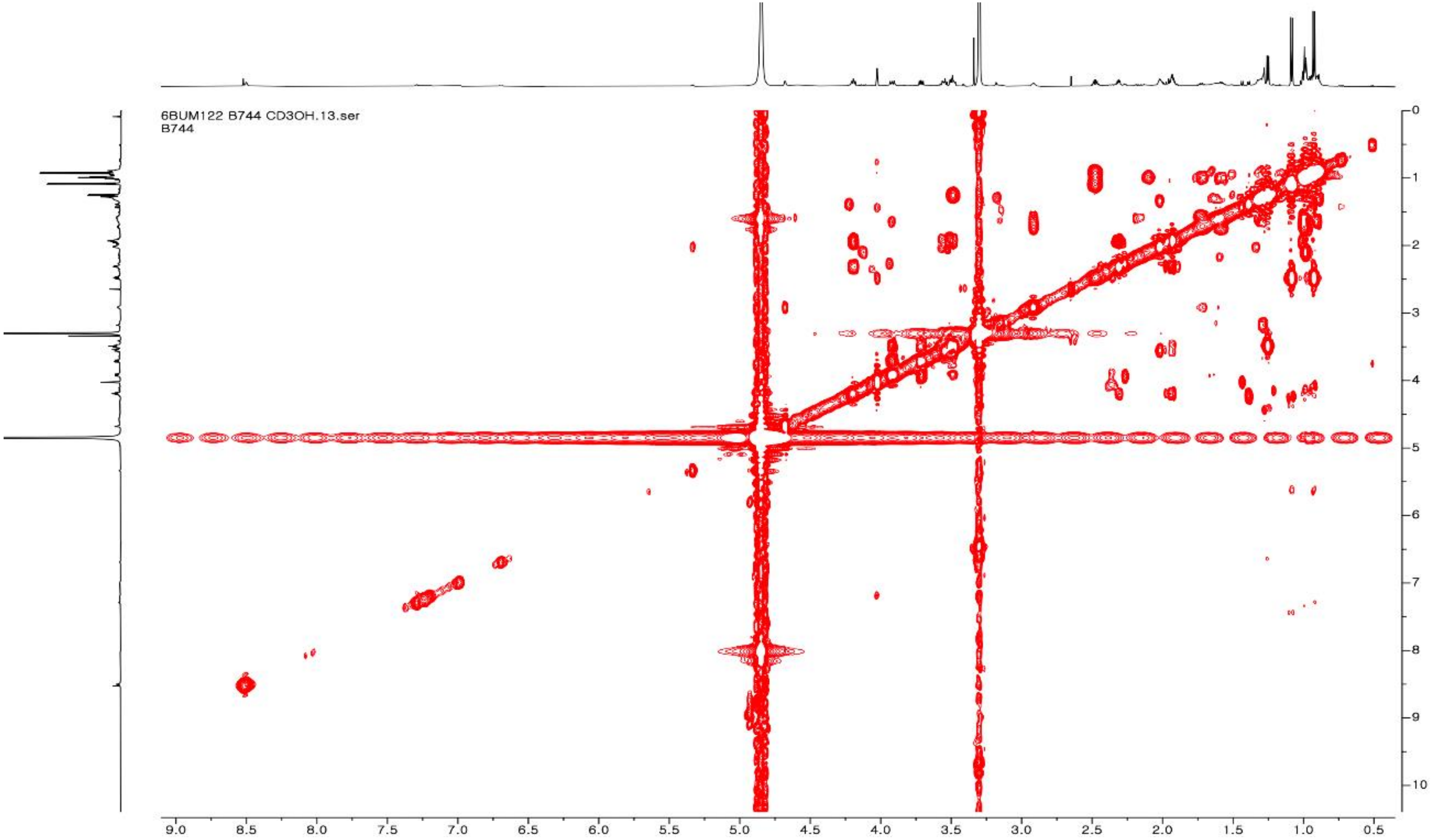

Supplementary Figure 30. COSY NMR spectrum of pachycephalamide B (**2**) in CD_3OD-d_4.

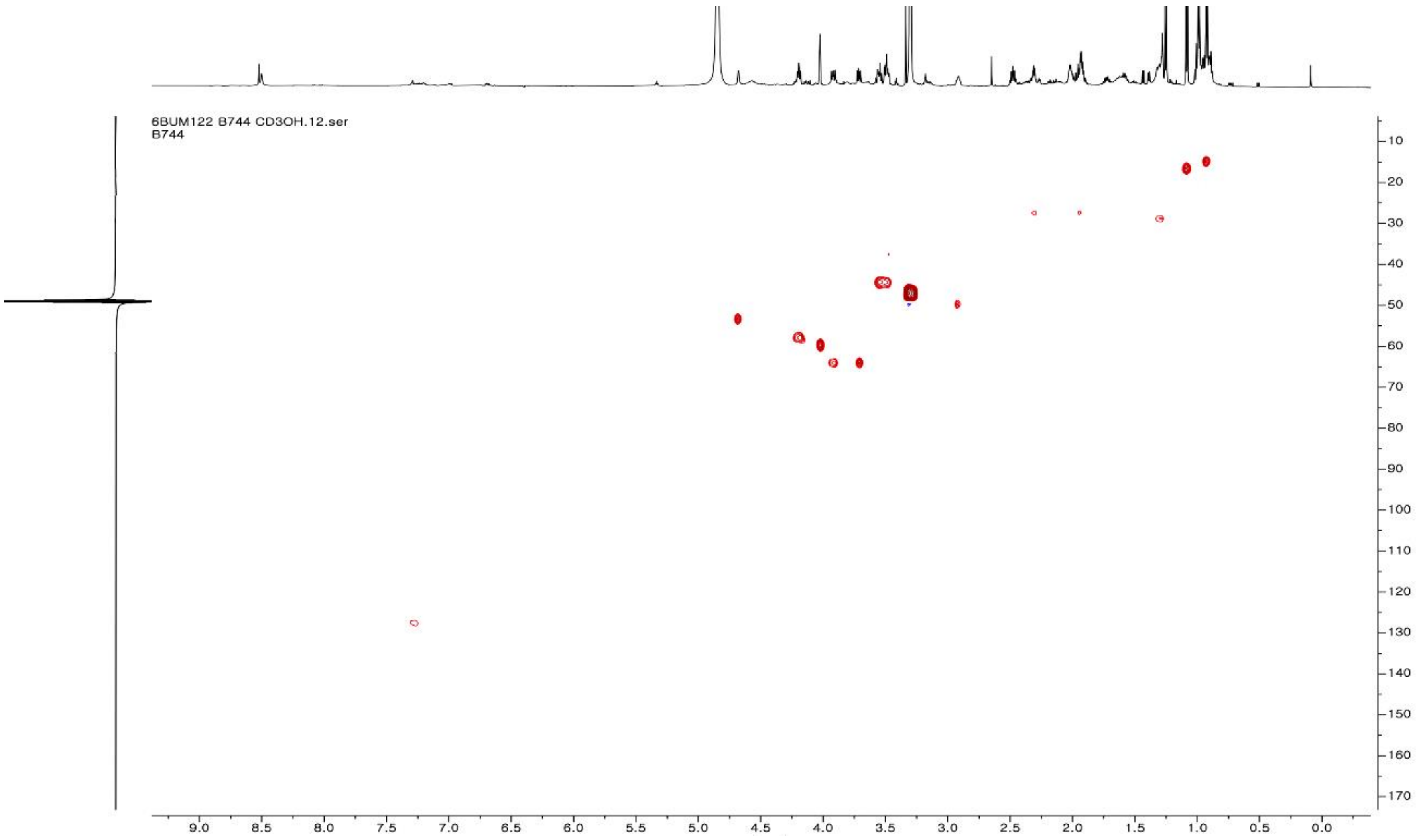

Supplementary Figure 31. HSQC NMR spectrum of pachycephalamide B (**2**) in CD_3OD-*d_4*.

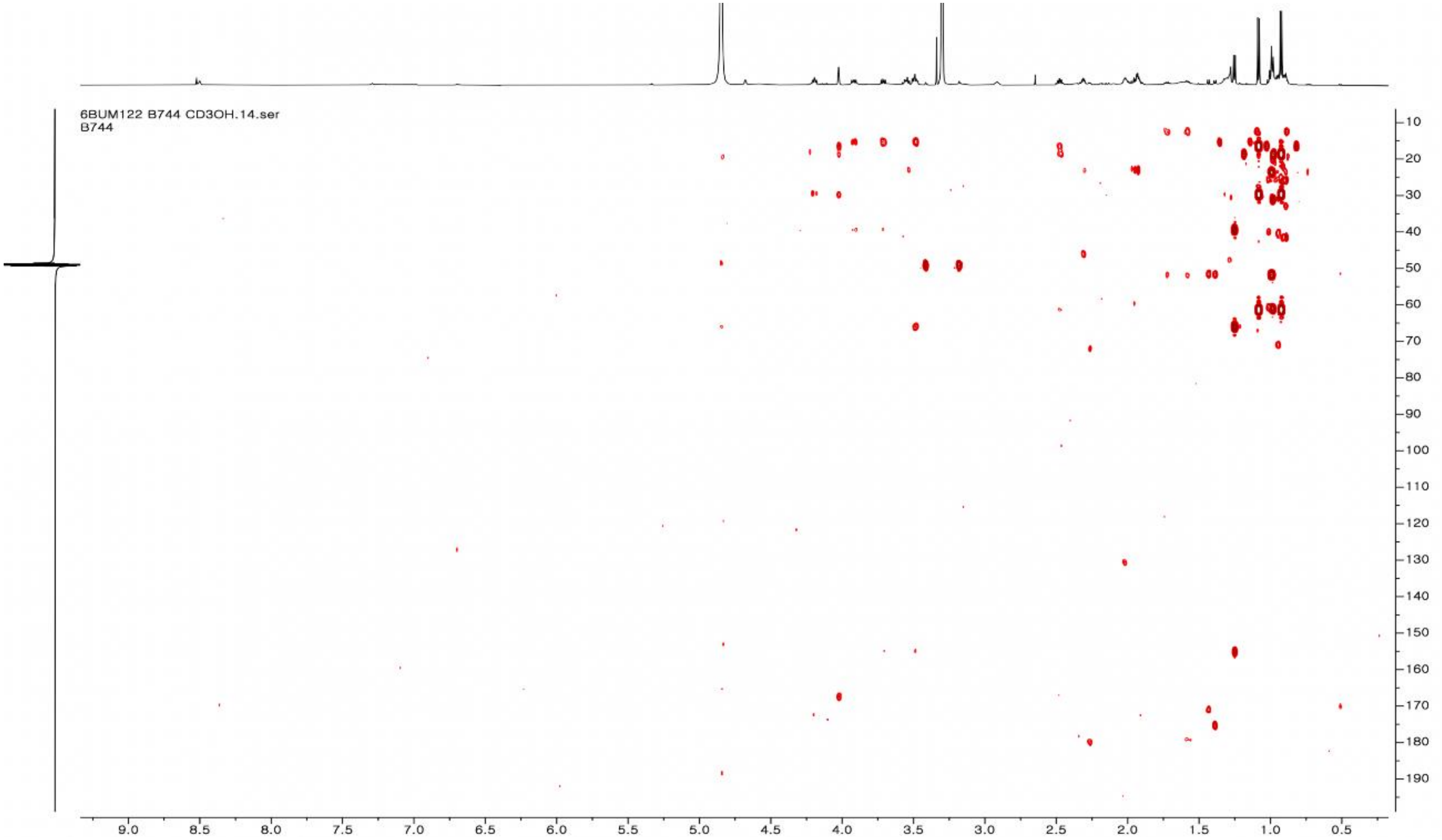

Supplementary Figure 32. HMBC NMR spectrum of pachycephalamide B (**2**) in CD_3OD-*d_4*.

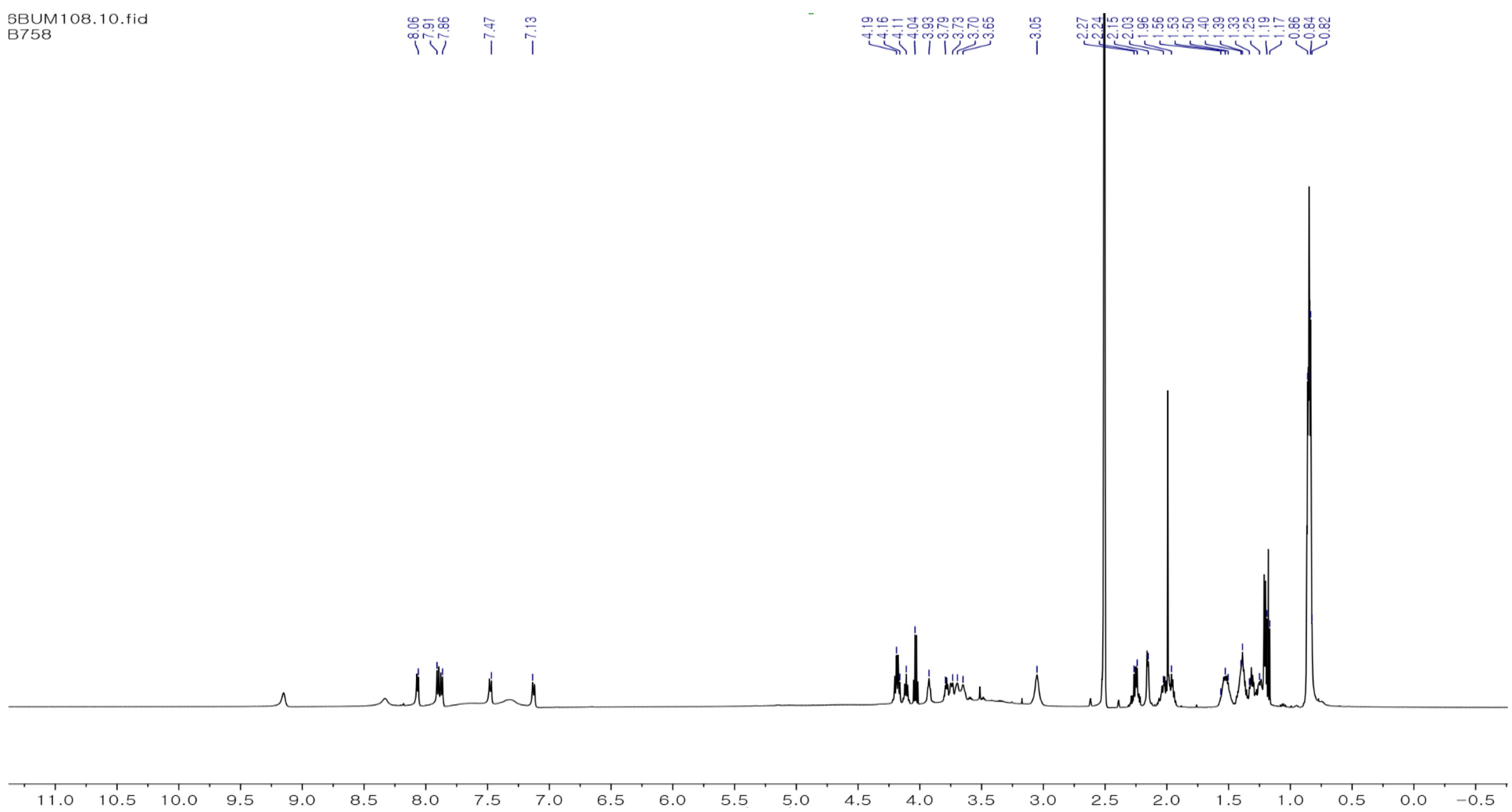

Supplementary Figure 33. ^{1}H NMR spectrum of pachycephalamide C (**3**) in CD_3OH-d_3.

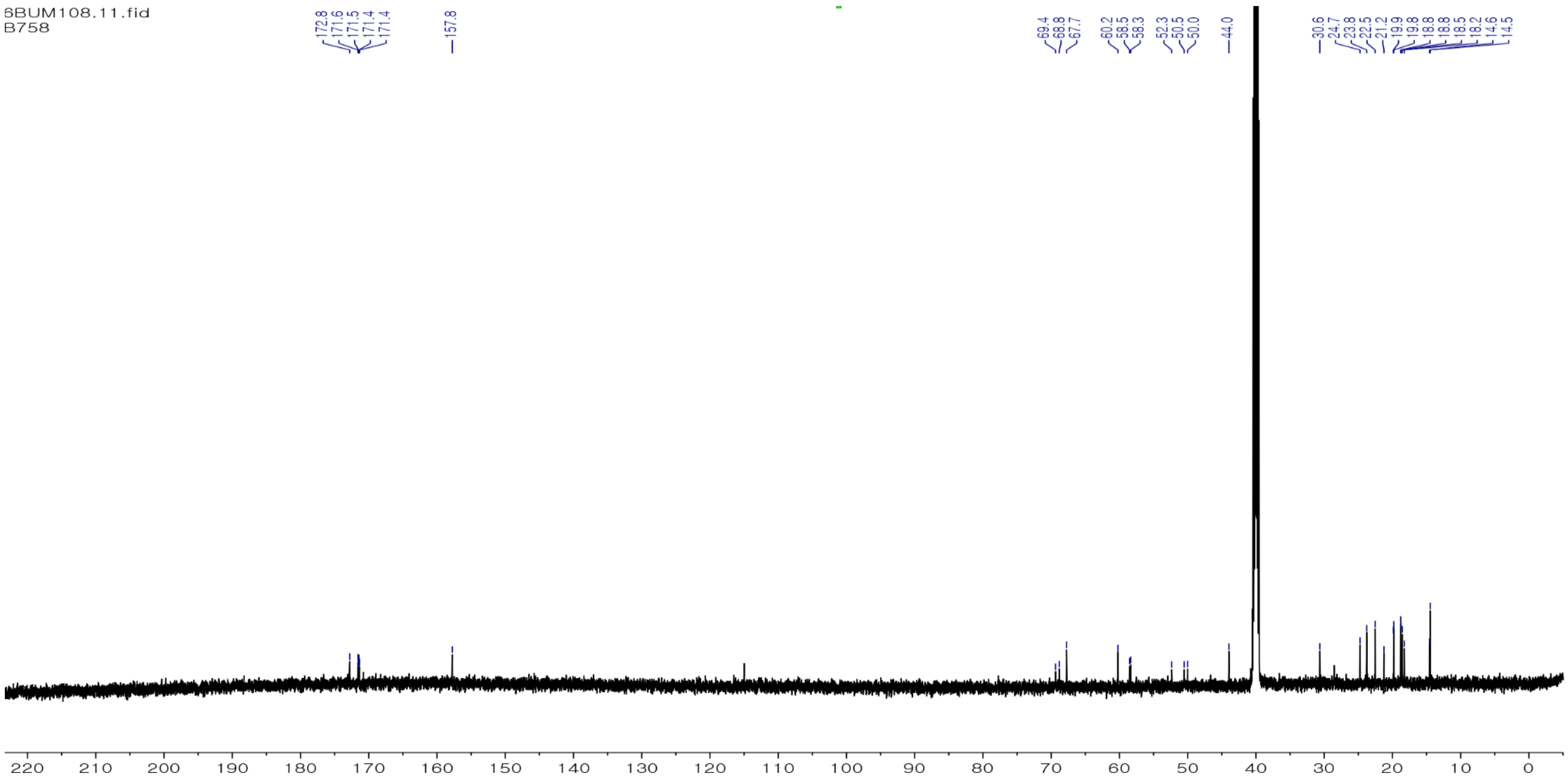

Supplementary Figure 34. ^{13}C NMR spectrum of pachycephalamide C (**3**) in CD_3OH-d_3.

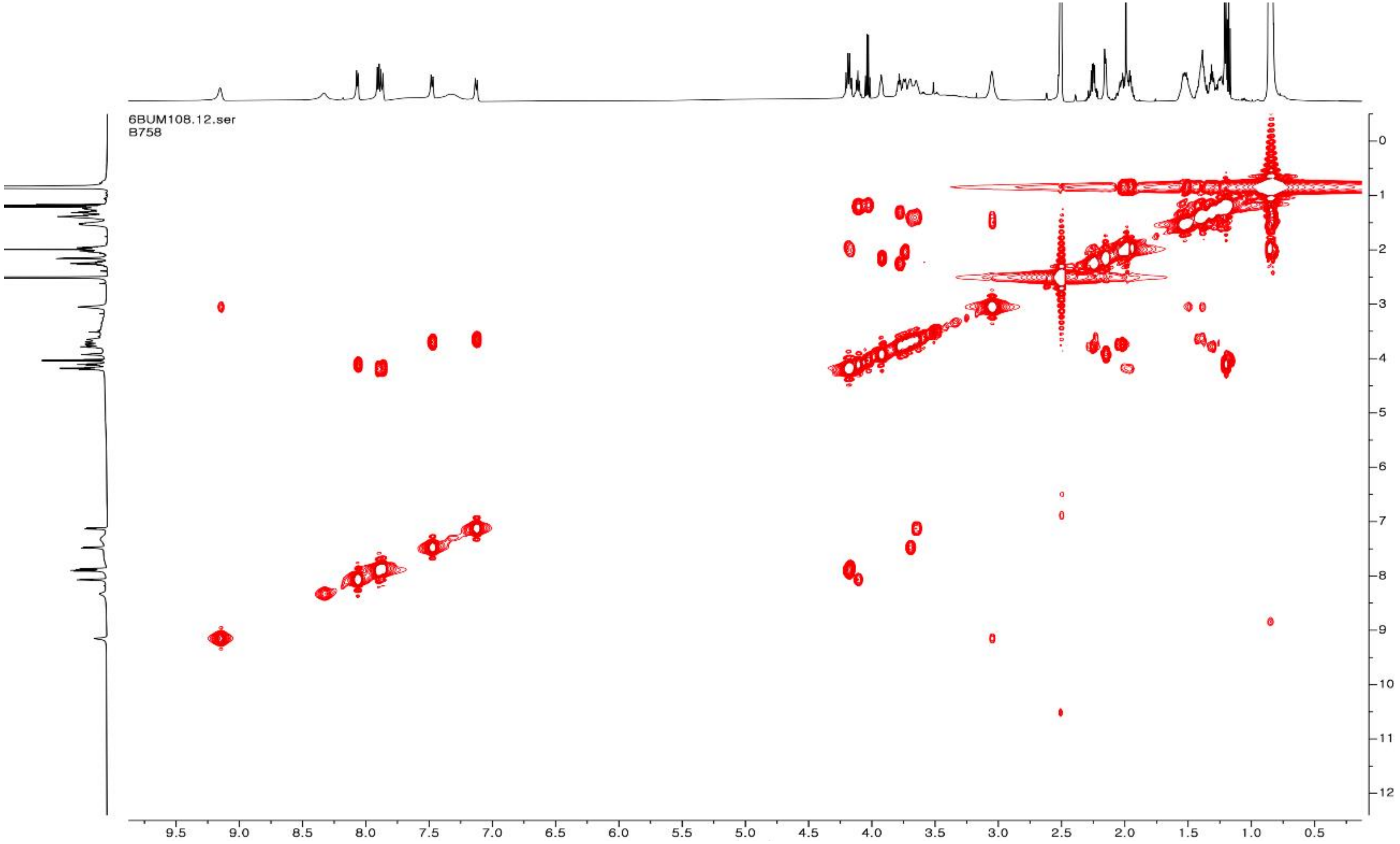

Supplementary Figure 35. COSY NMR spectrum of pachycephalamide C (**3**) in CD_3OH-d_3.

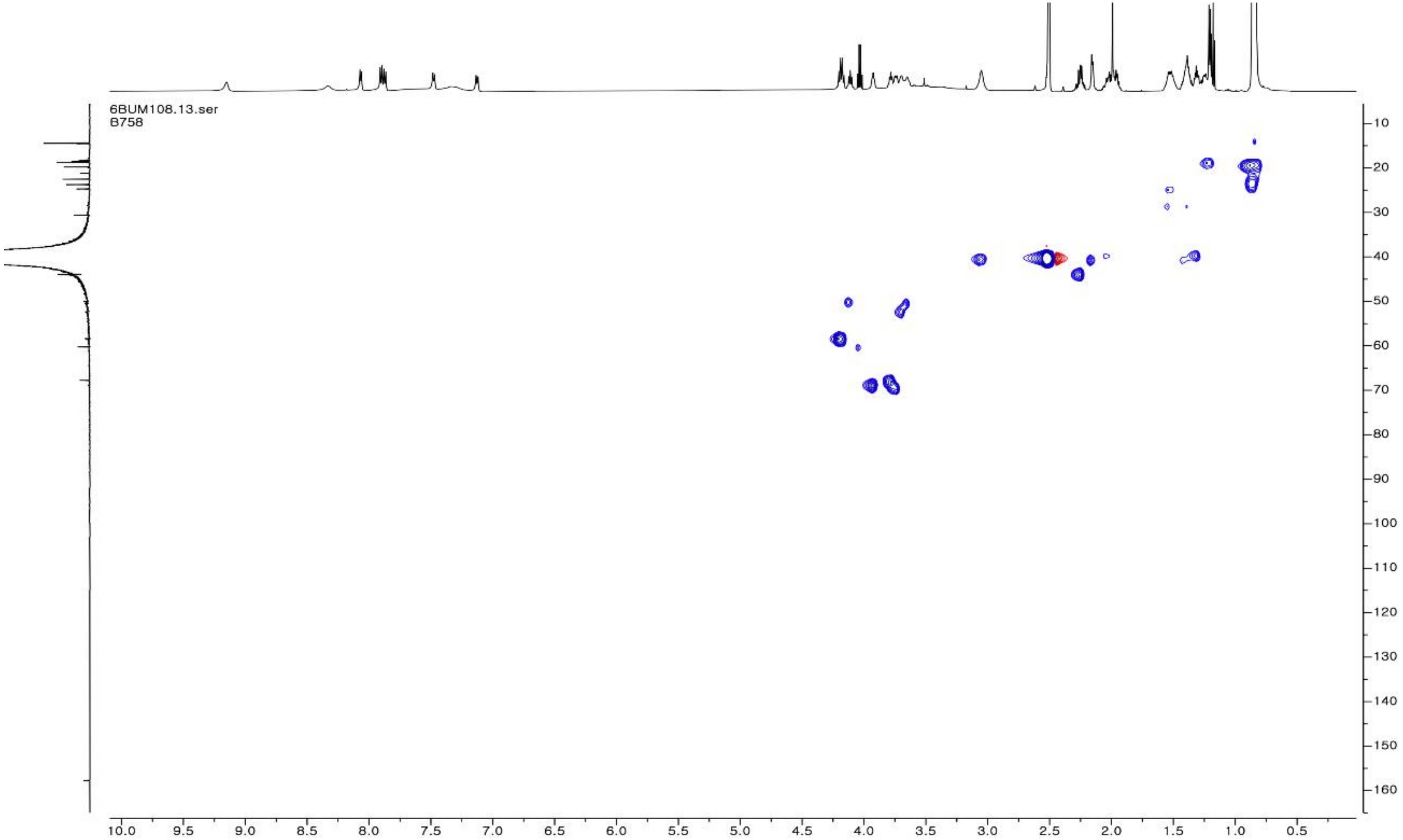

Supplementary Figure 36. HSQC NMR spectrum of pachycephalamide C (**3**) in CD_3OH-d_3.

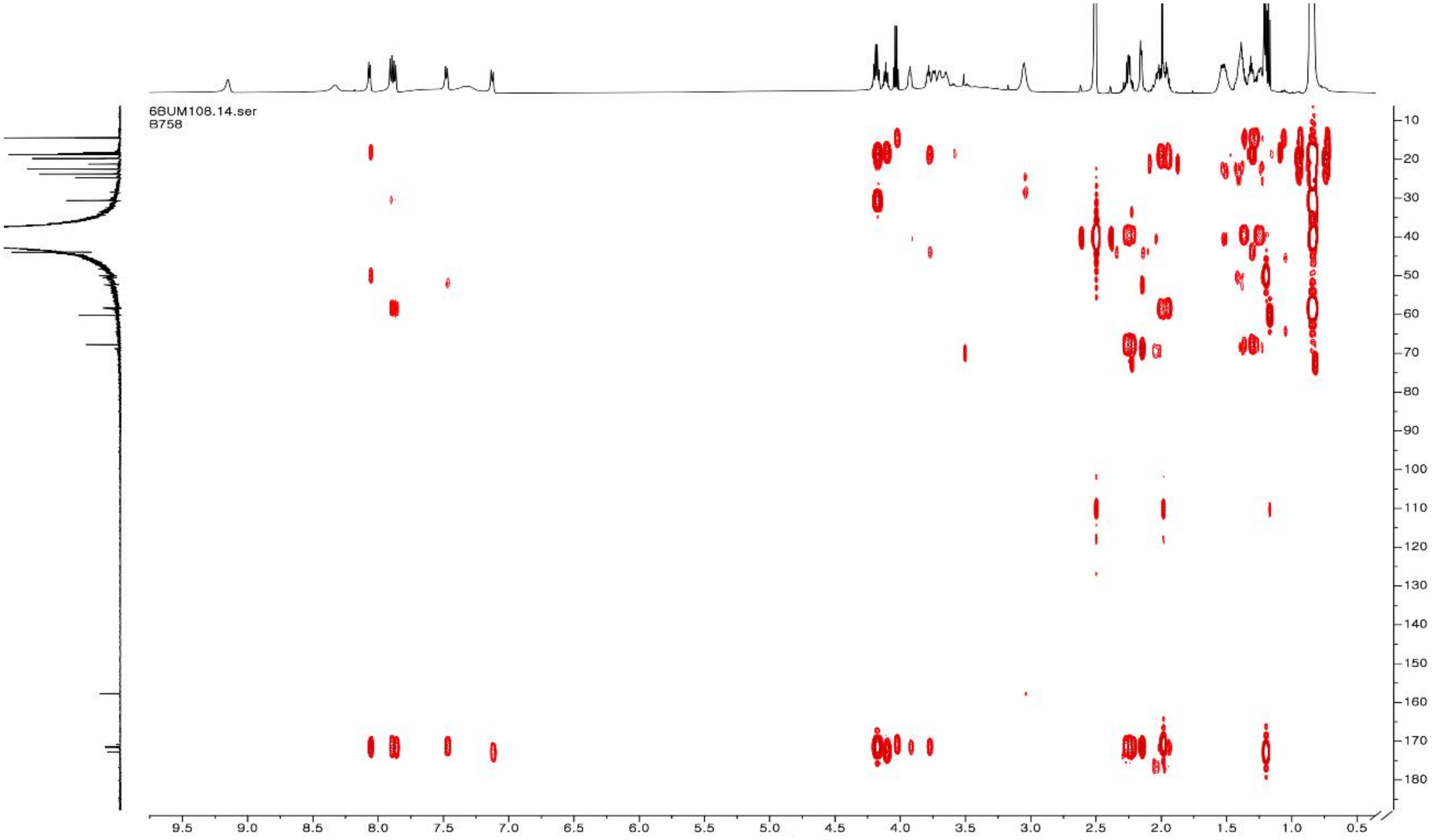

Supplementary Figure 37. HMBC NMR spectrum of pachycephalamide C (**3**) in CD_3OH-d_3.

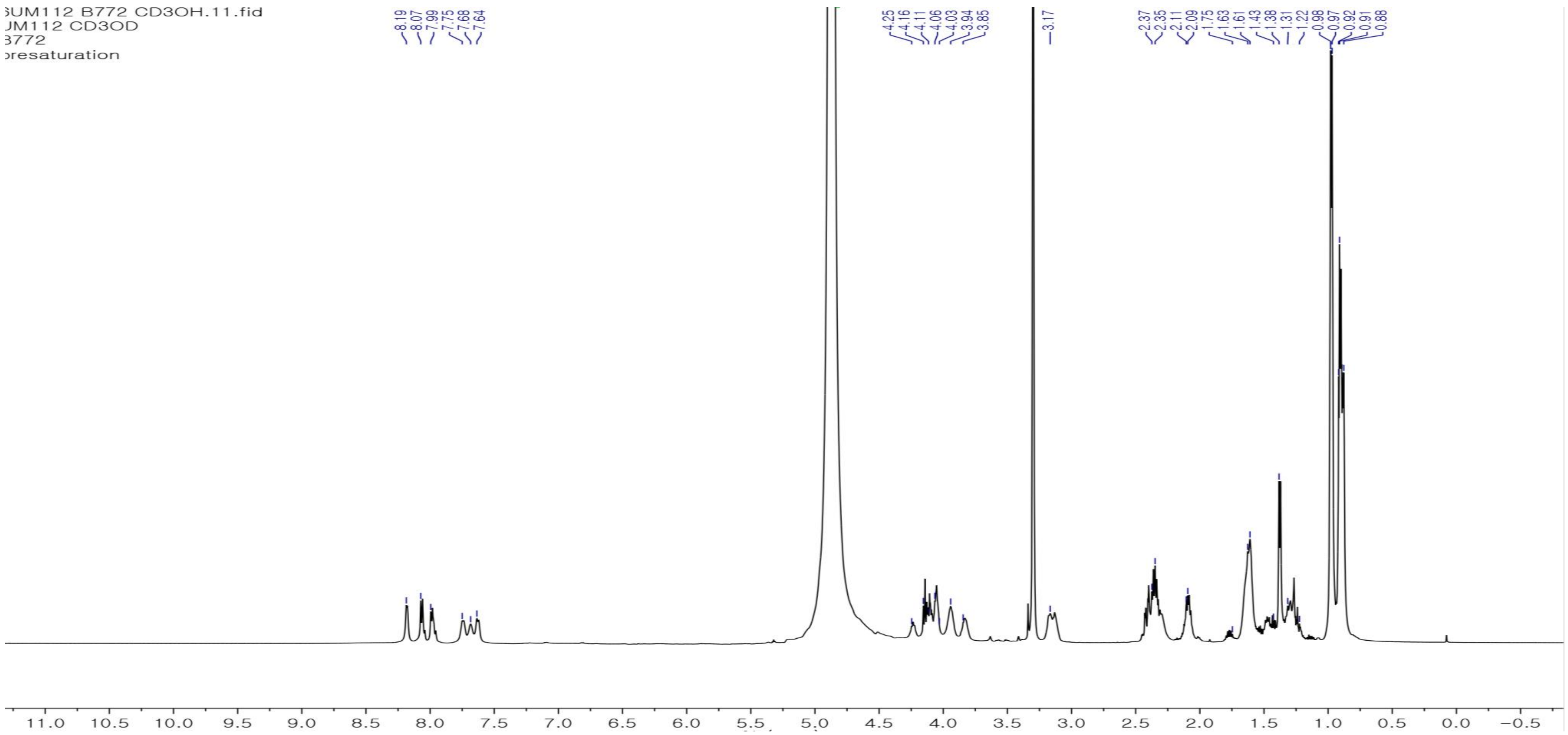

Supplementary Figure 38. ^{1}H NMR spectrum of pachycephalamide D (**4**) in CD_3OH-d_3.

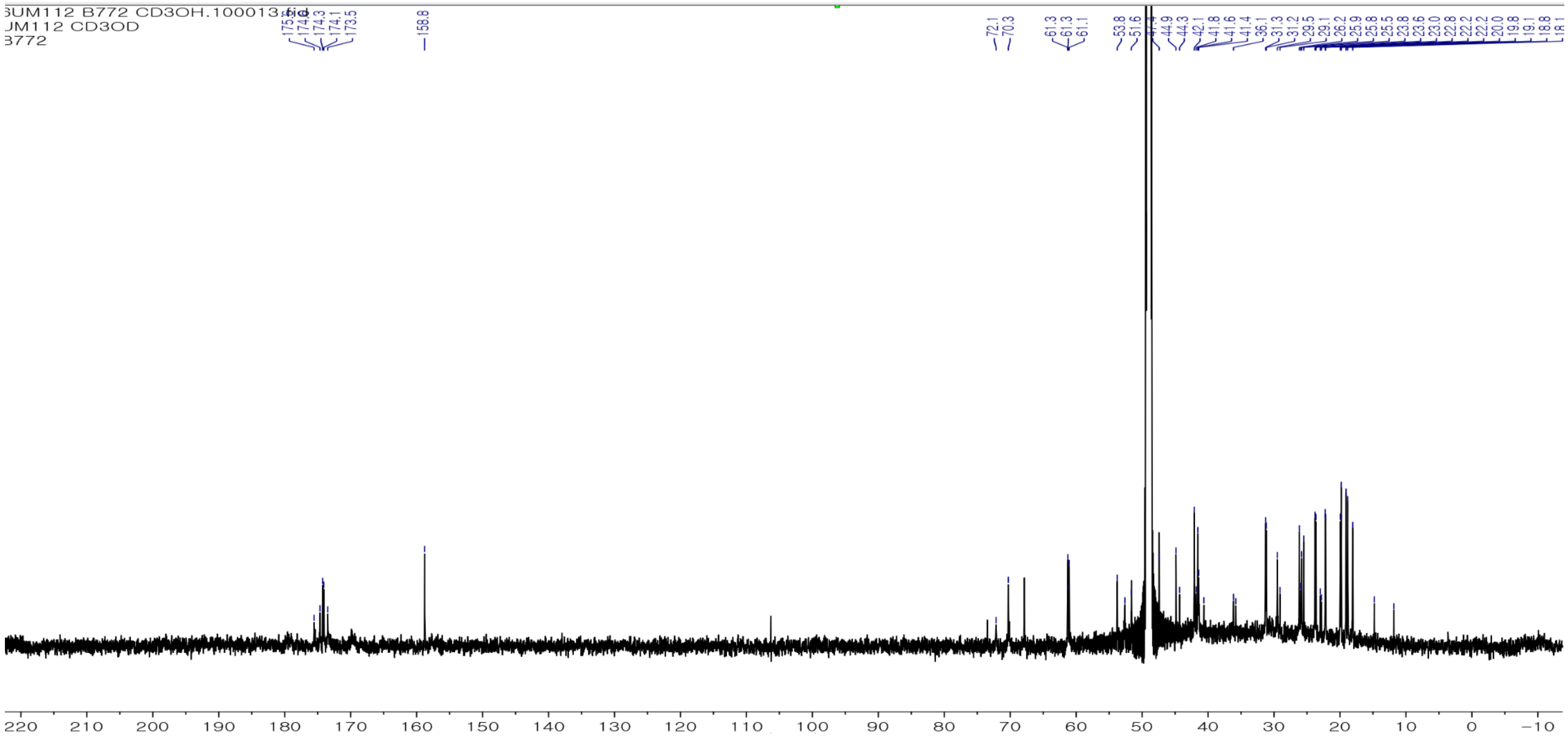

Supplementary Figure 39. ^{13}C NMR spectrum of pachycephalamide D (**4**) in CD_3OH-d_3.

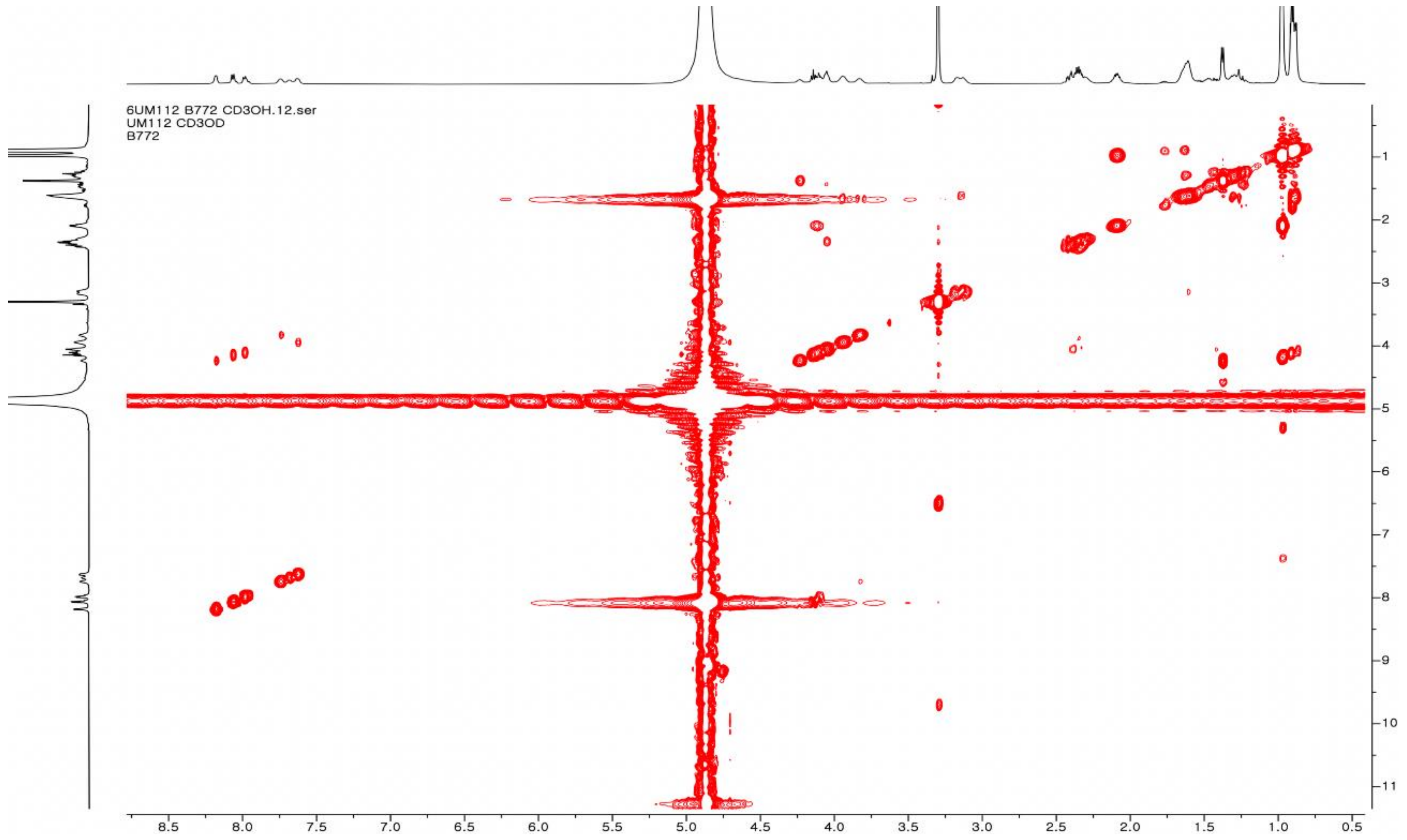

Supplementary Figure 40. COSY NMR spectrum of pachycephalamide D (**4**) in CD_3OH-d_3.

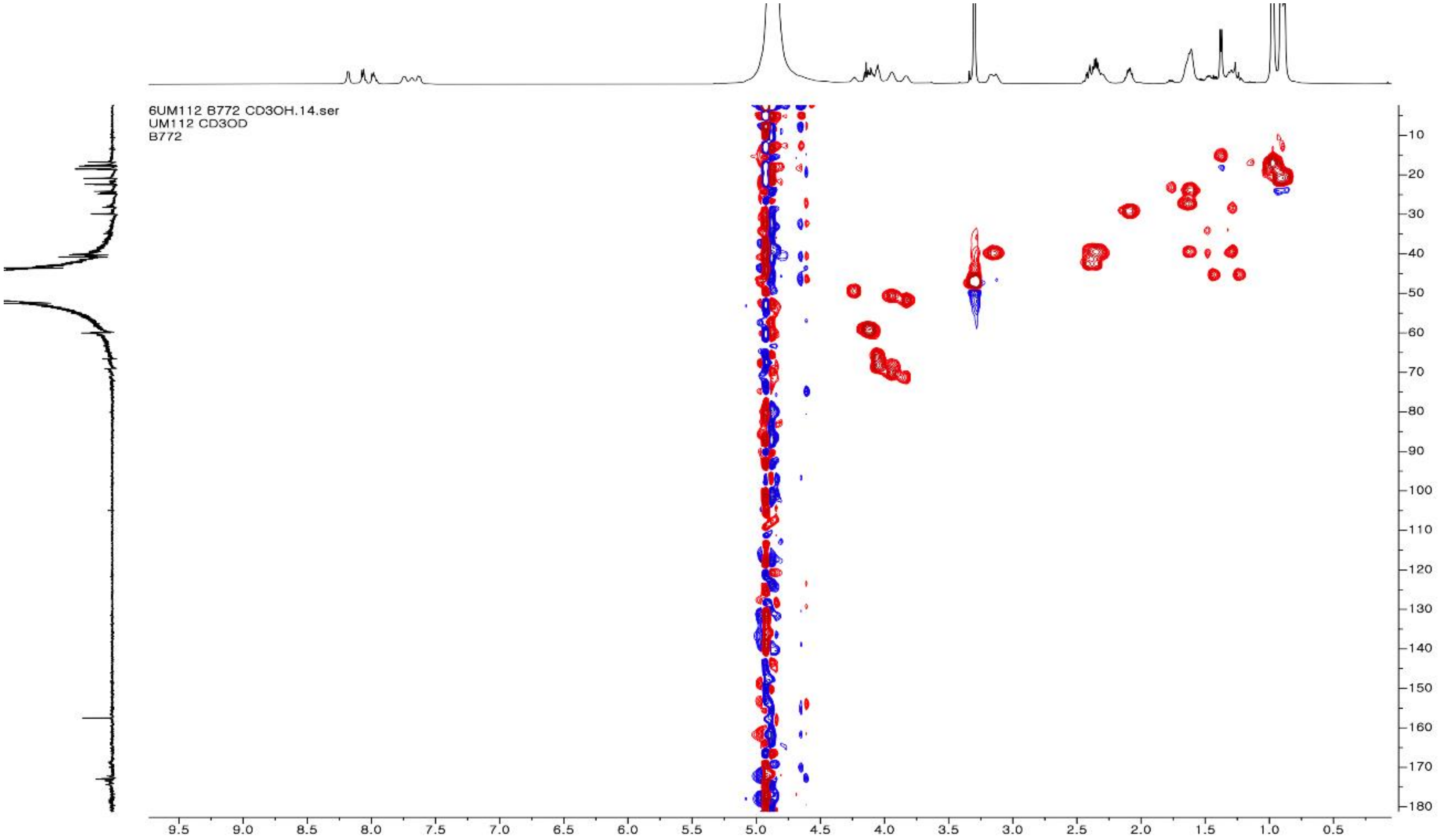

Supplementary Figure 41. HSQC NMR spectrum of pachycephalamide D (**4**) in CD_3OH-d_3.

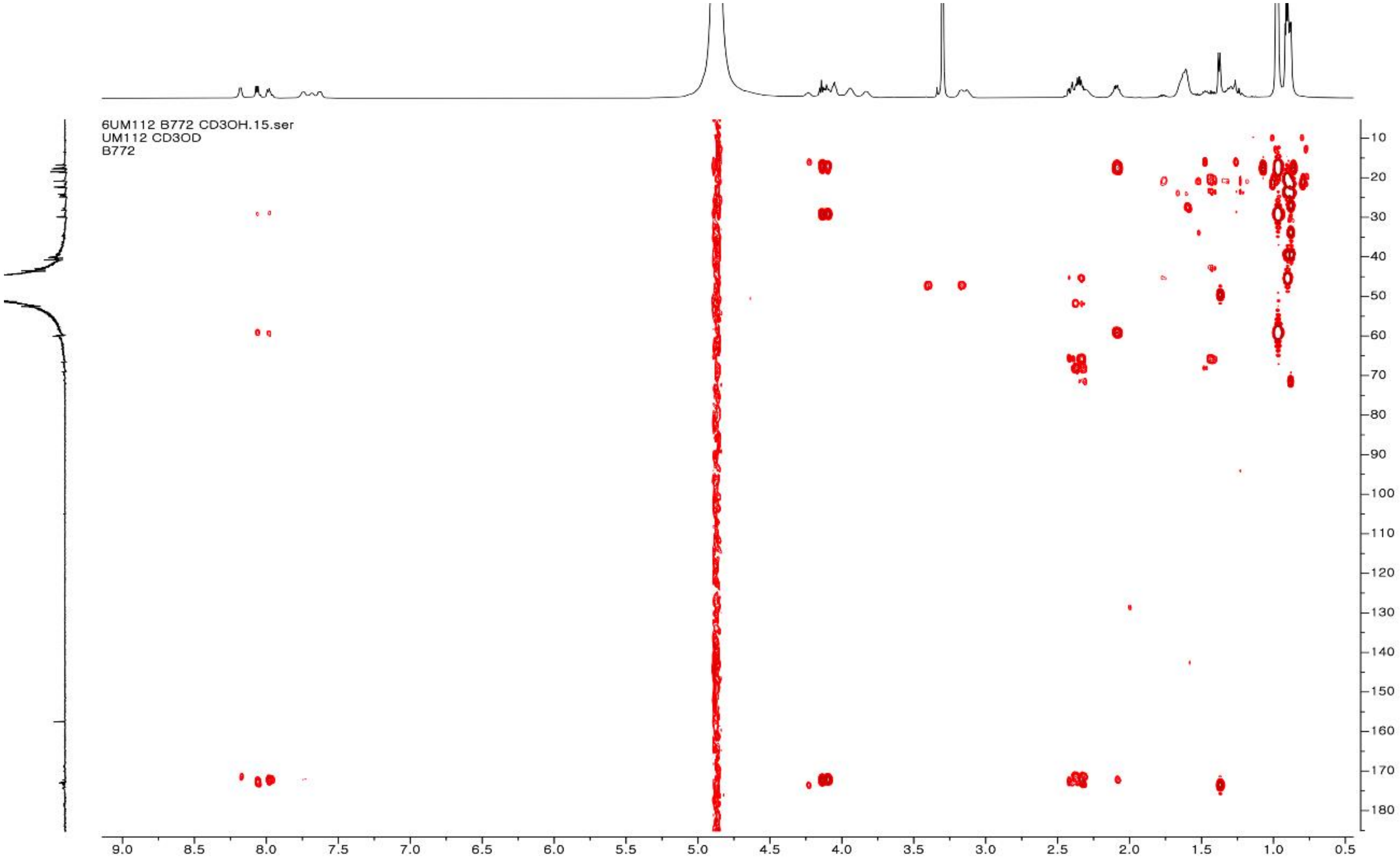

Supplementary Figure 42. HMBC NMR spectrum of pachycephalamide D (**4**) in CD_3OH-d_3.

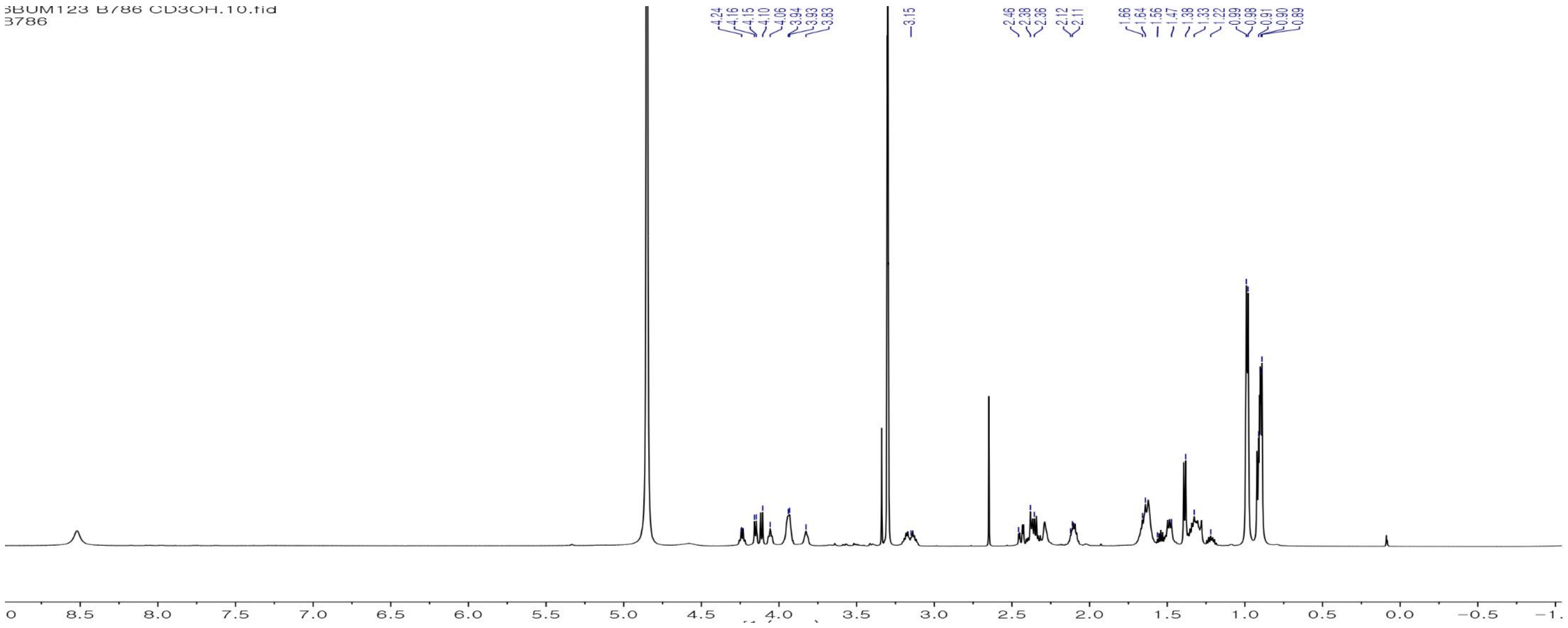

Supplementary Figure 43. ^{1}H NMR spectrum of pachycephalamide E (**5**) in CD_3OD-d_4.

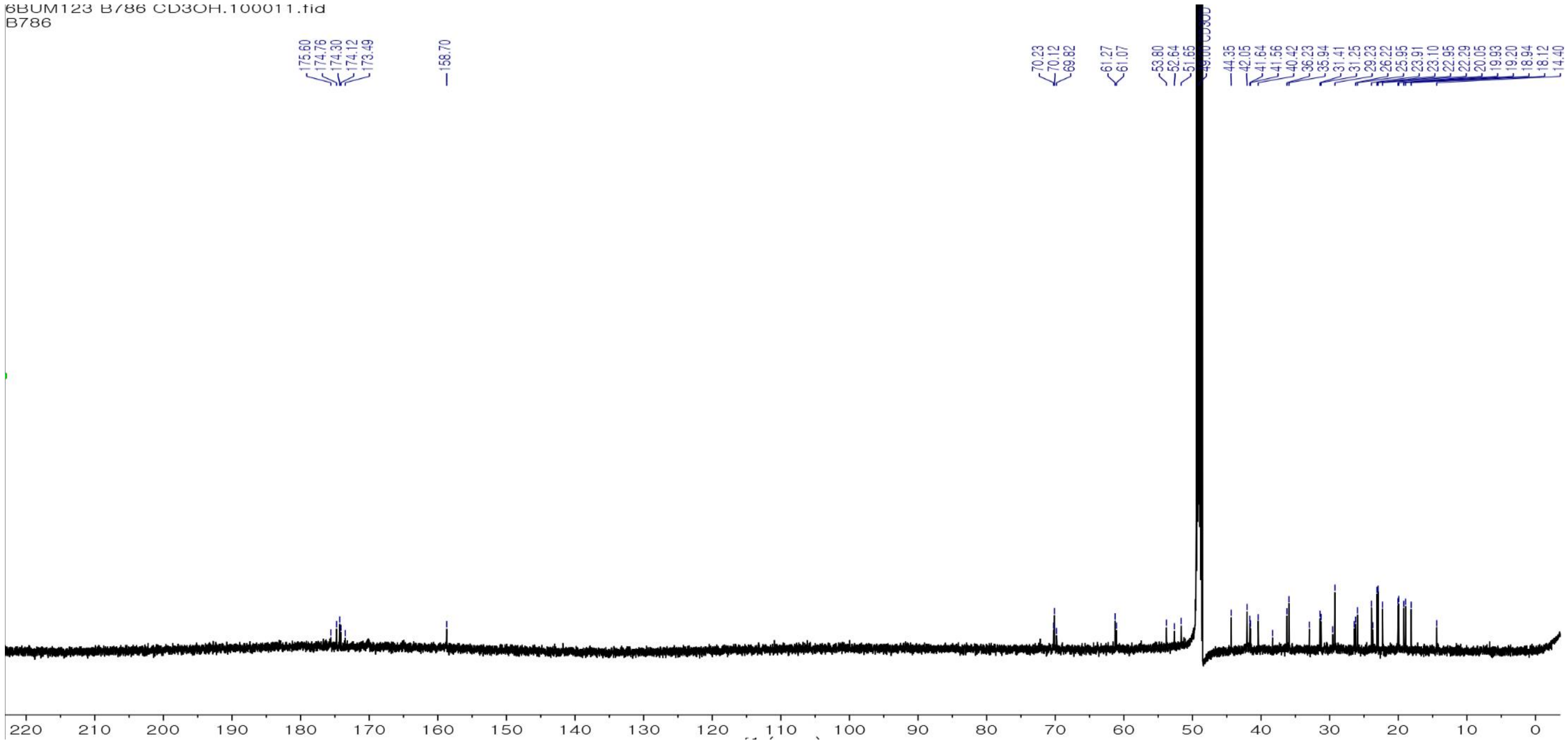

Supplementary Figure 44. ^{13}C NMR spectrum of pachycephalamide E (**5**) in CD_3OD-d_4.

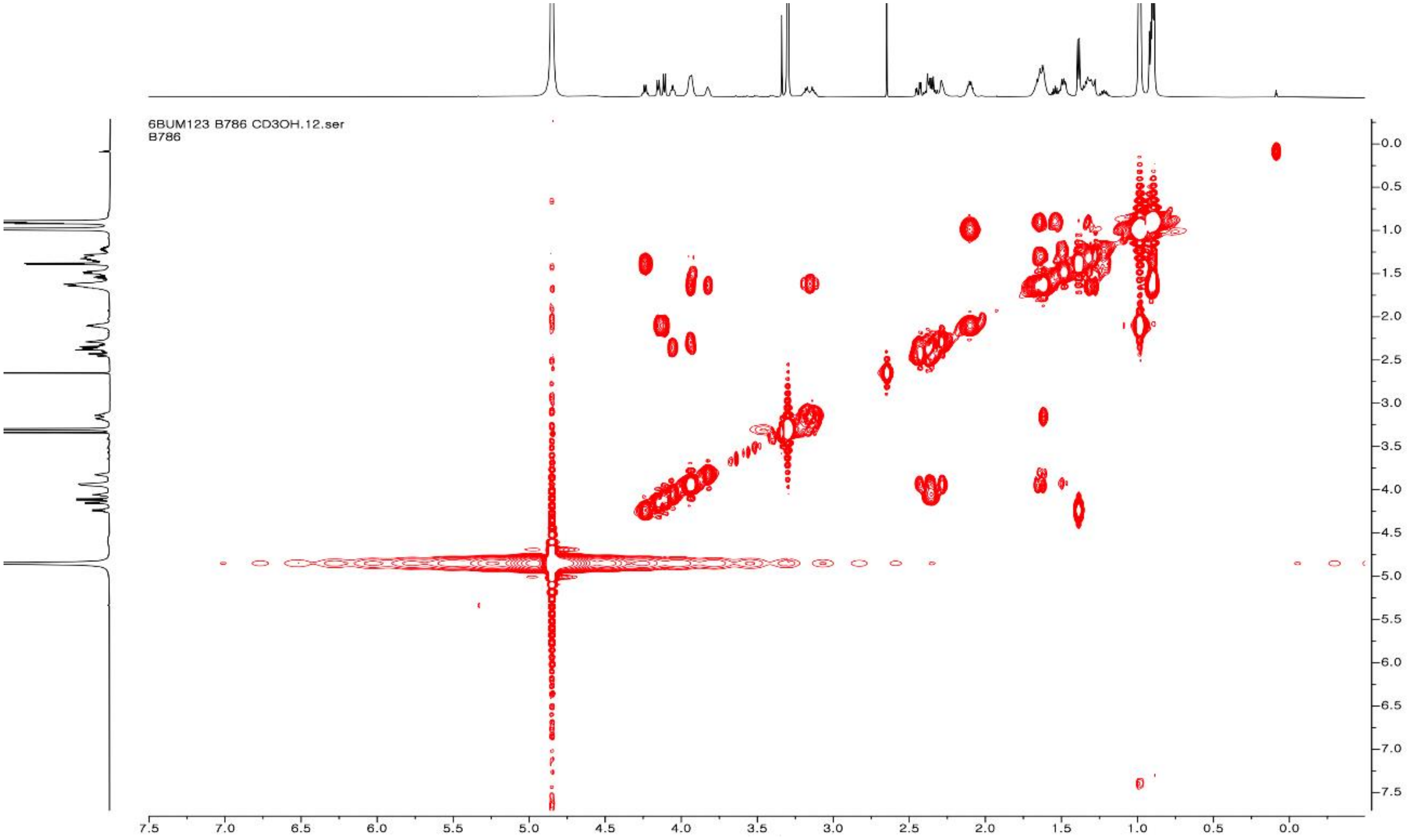

Supplementary Figure 45. COSY NMR spectrum of pachycephalamide E (**5**) in CD_3OD-*d*$_4$.

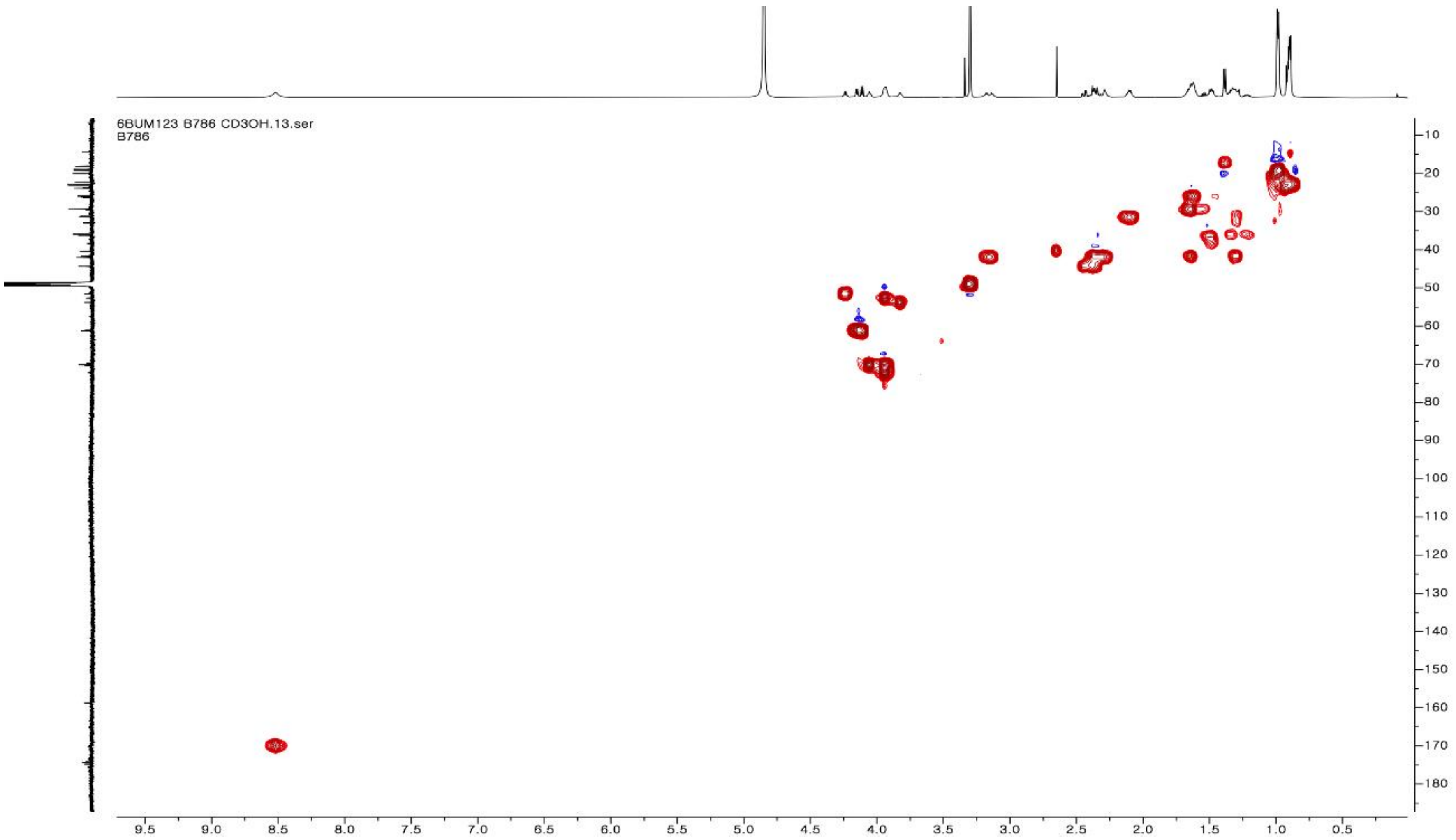

Supplementary Figure 46. HSQC NMR spectrum of pachycephalamide E (**5**) in CD_3OD-d_4.

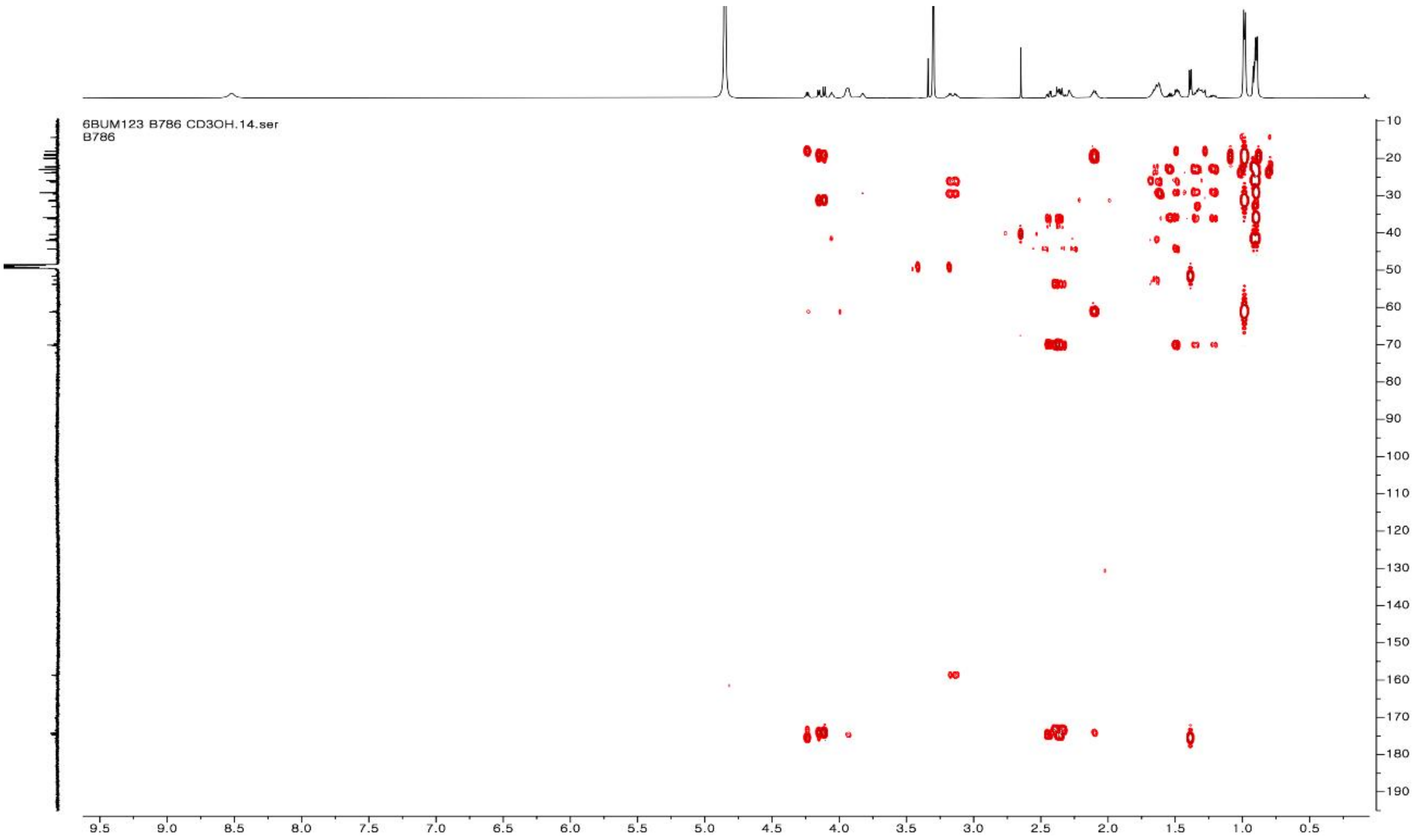

Supplementary Figure 47. HMBC NMR spectrum of pachycephalamide E (**5**) in CD_3OD-d_4.

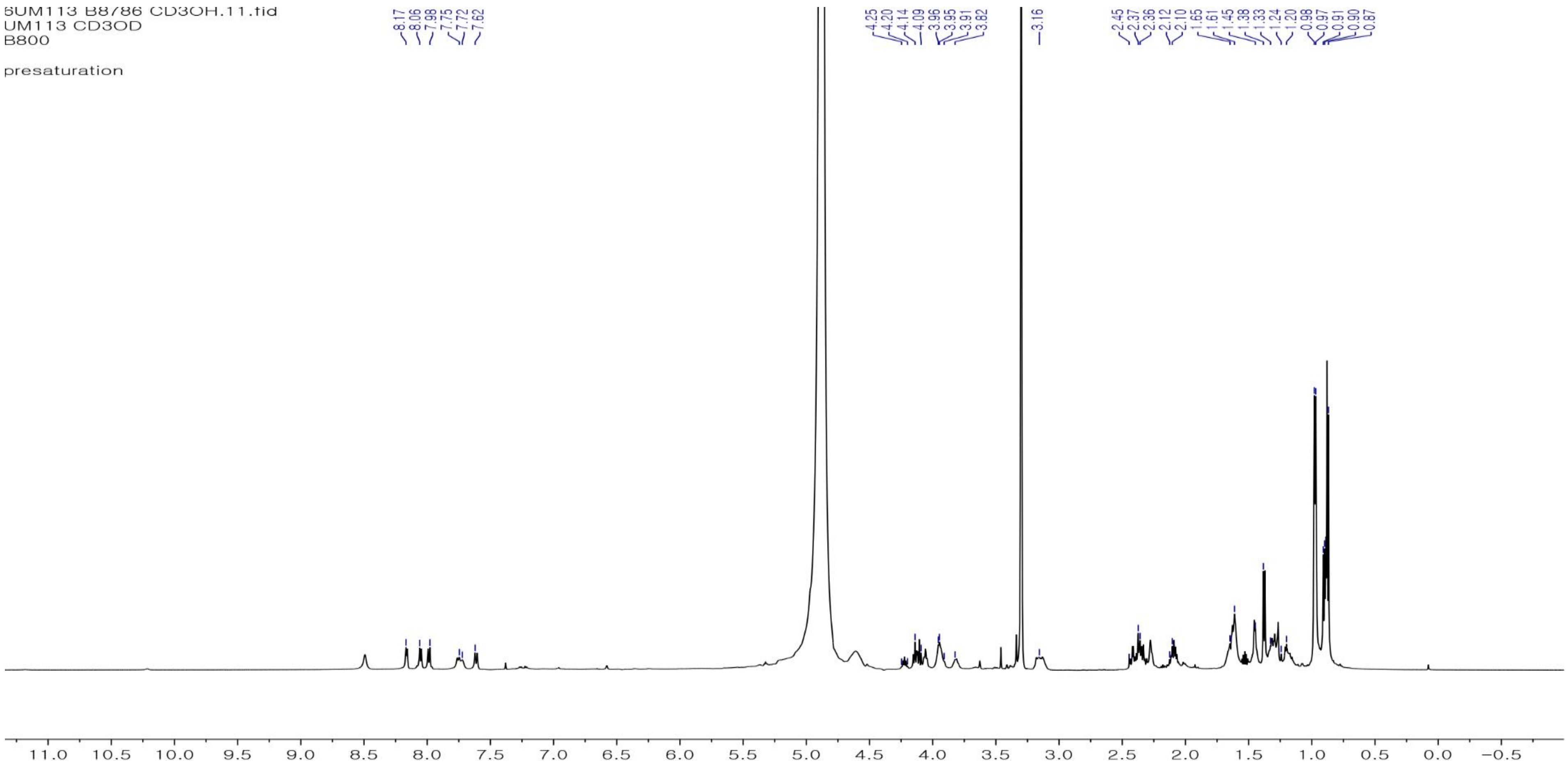

Supplementary Figure 48. ^{1}H NMR spectrum of pachycephalamide F (**6**) in CD_3OH-d_3.

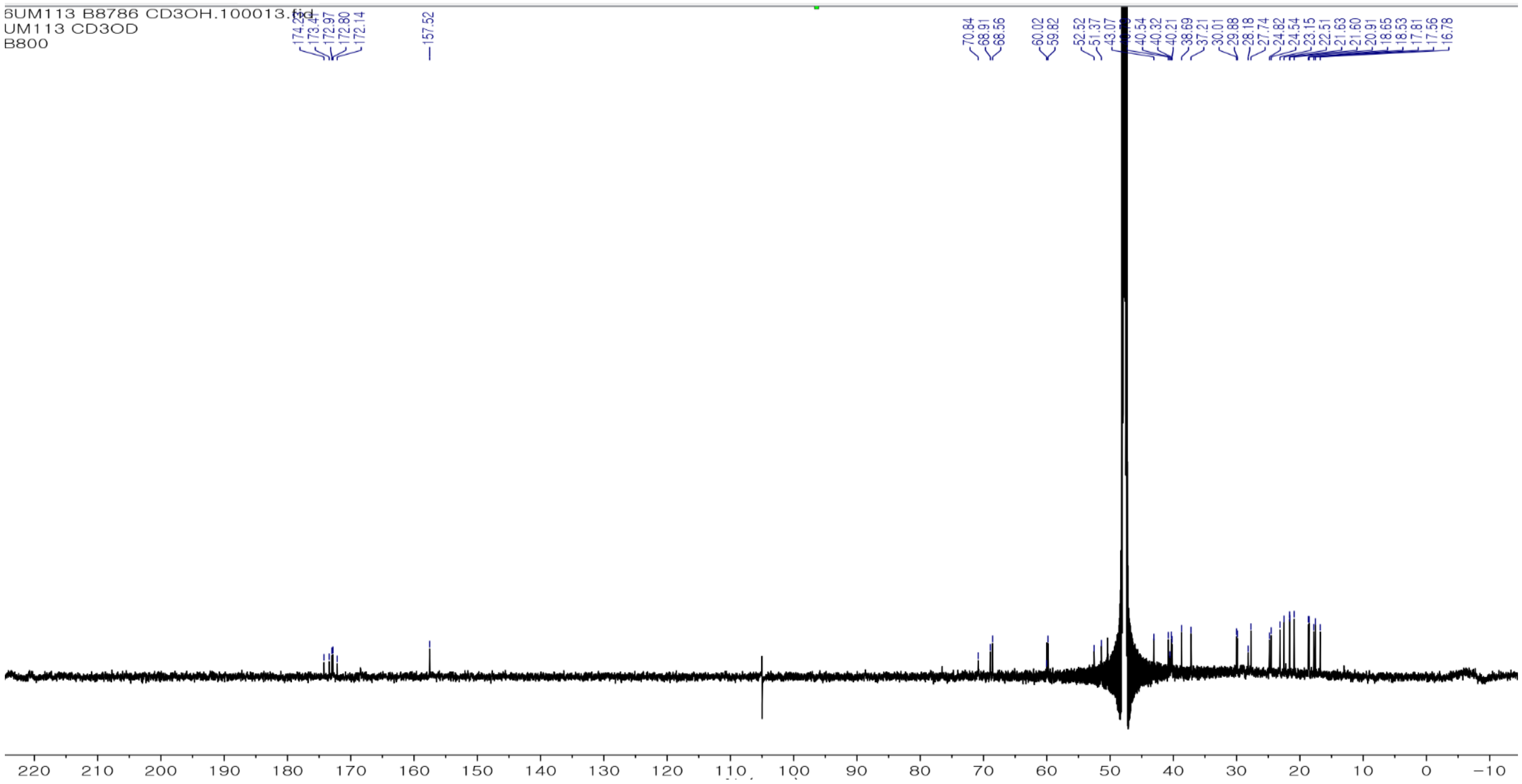

Supplementary Figure 49. ^{13}C NMR spectrum of pachycephalamide F (**6**) in CD_3OH-d_3.

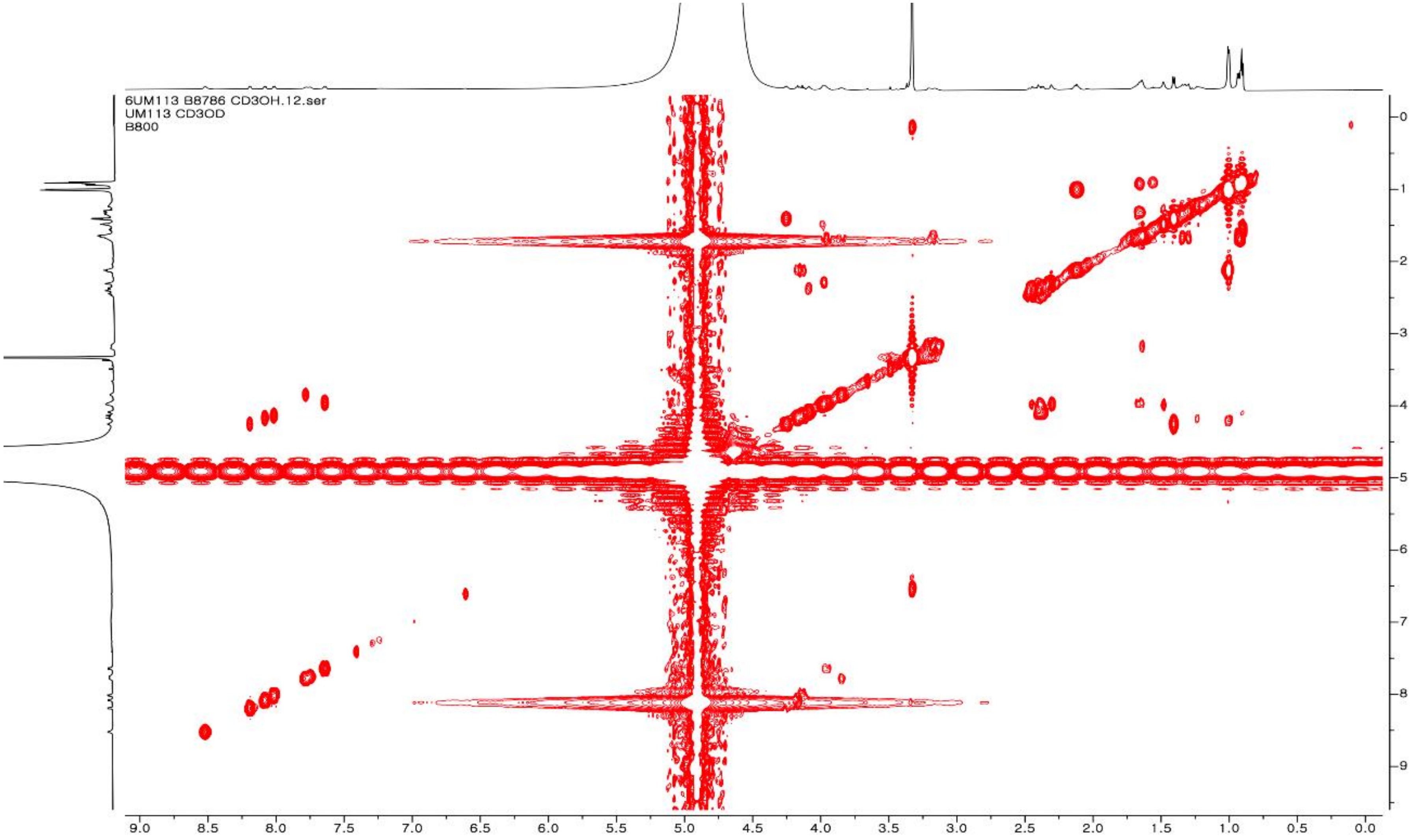

Supplementary Figure 50. COSY NMR spectrum of pachycephalamide F (**6**) in CD_3OH-d_3.

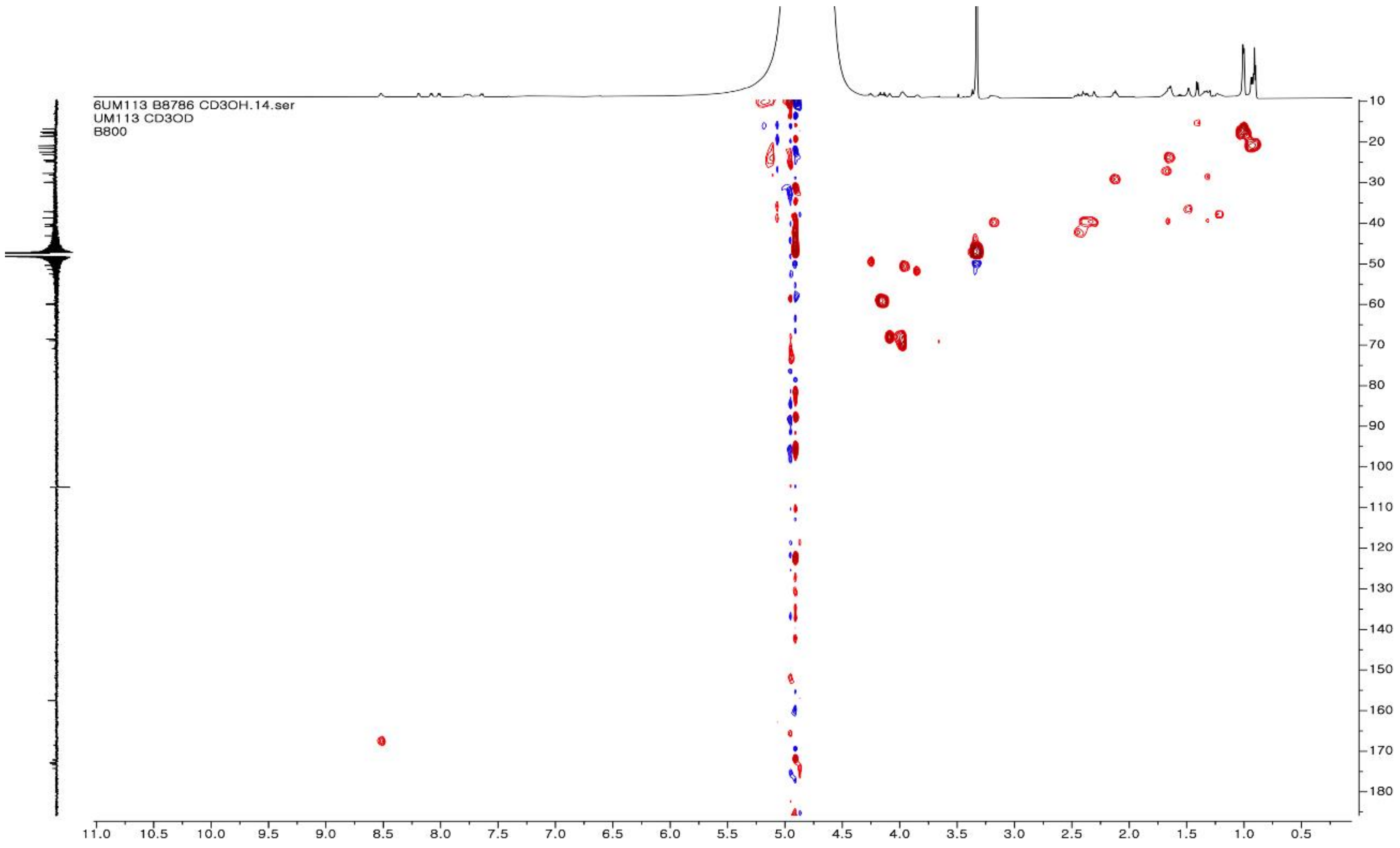

Supplementary Figure 51. HSQC NMR spectrum of pachycephalamide F (**6**) in CD_3OH-d_3.

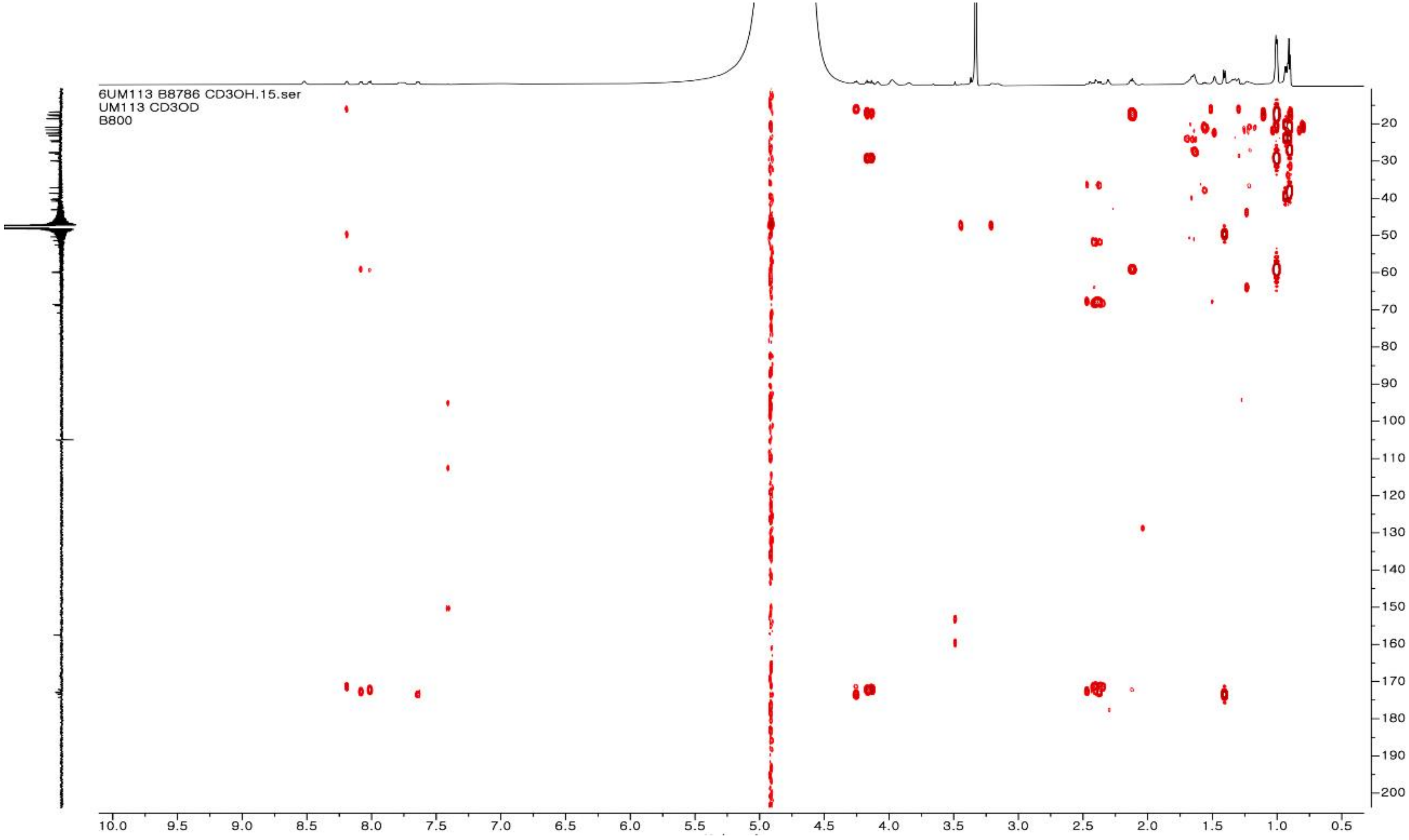

Supplementary Figure 52. HMBC NMR spectrum of pachycephalamide F (**6**) in CD_3OH-d_3.

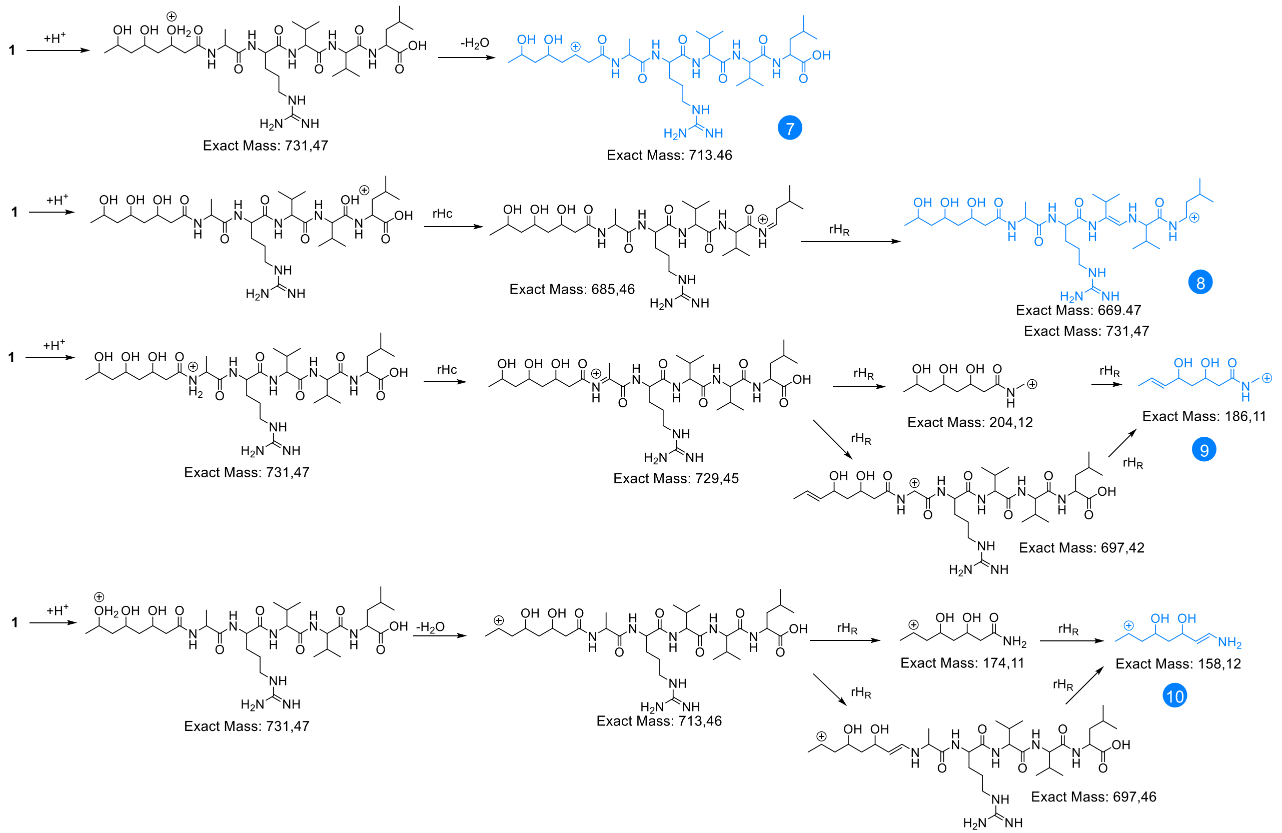

Supplementary Figure 53. Mechanisms of MS/MS fragment formation for pachycephalamides A (1). Mechanism for fragments matching calculated *m/z* values were predicted using MassFrontier 8.0 (Thermo Fisher Scientific). Fragments which matched the prediction of MassFrontier 8.0 are marked with red, blue or black circles. Fragments 1-6 represent fragments of the common peptide scaffold after cleavage of the varying fatty acid moiety and are identical among the spectra of pachycephalamide A-F. Fragments 7-10 appear in each spectrum of pachycephalamide A-F, and are proposed to be part of the varying fatty acid moiety; therefore *m/z* values differ by 14 Da (CH_2) between two consecutive spectra. However, the *m/z* differences of these fragments to the corresponding parent ion (molecule ion, $[M+H]^+$) are identical for all spectra (visualized by blue arrows).

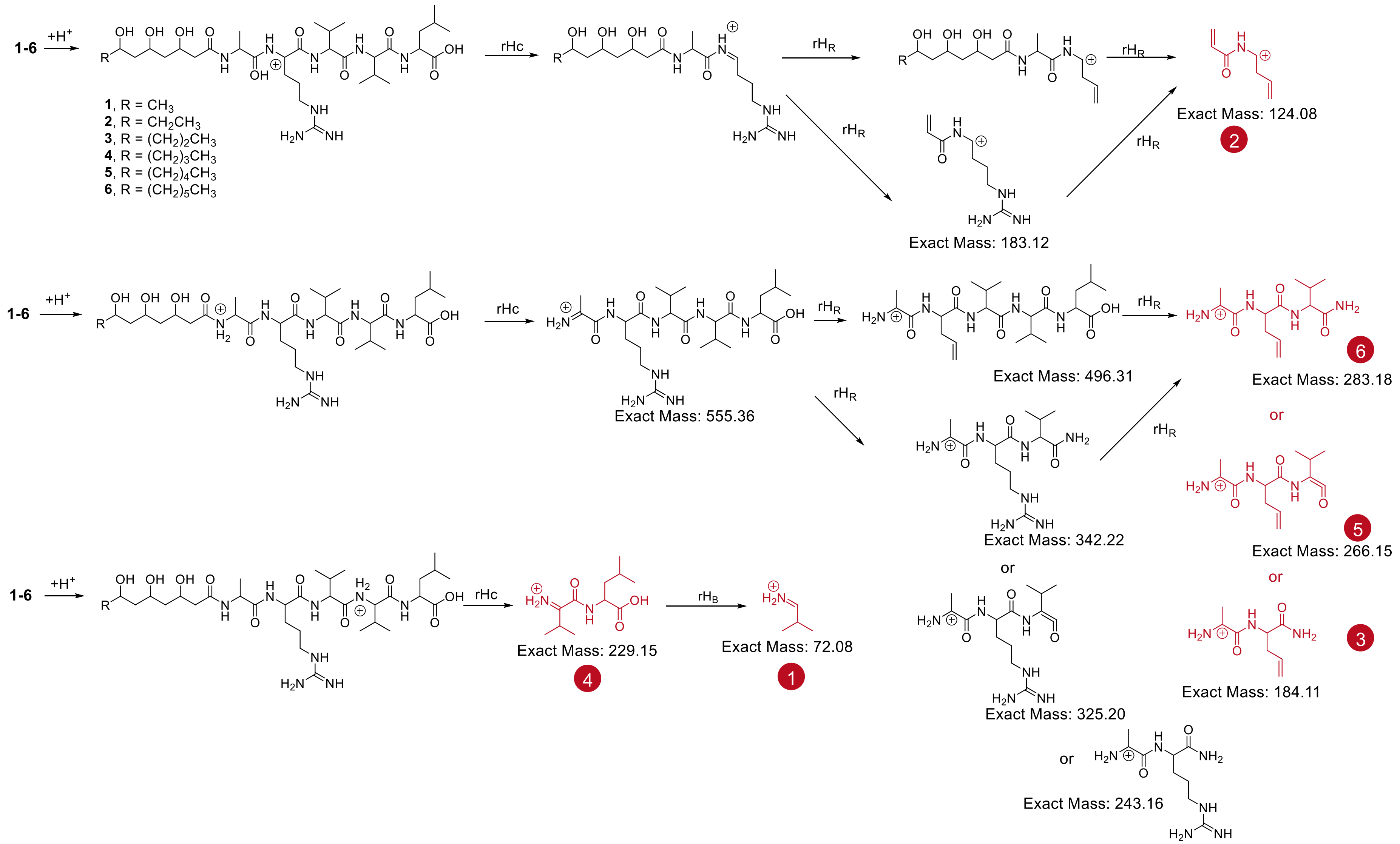

Supplementary Figure 54. Mechanisms of MS/MS fragment formation for pachycephalamides A-F (1-6). Mechanism for fragments matching calculated m/z values were predicted using MassFrontier 8.0. Fragments 1-6 are identical among the spectra of pachycephalamide A-F and represent fragments of the common peptide scaffold after cleavage of the varying fatty acid moiety.

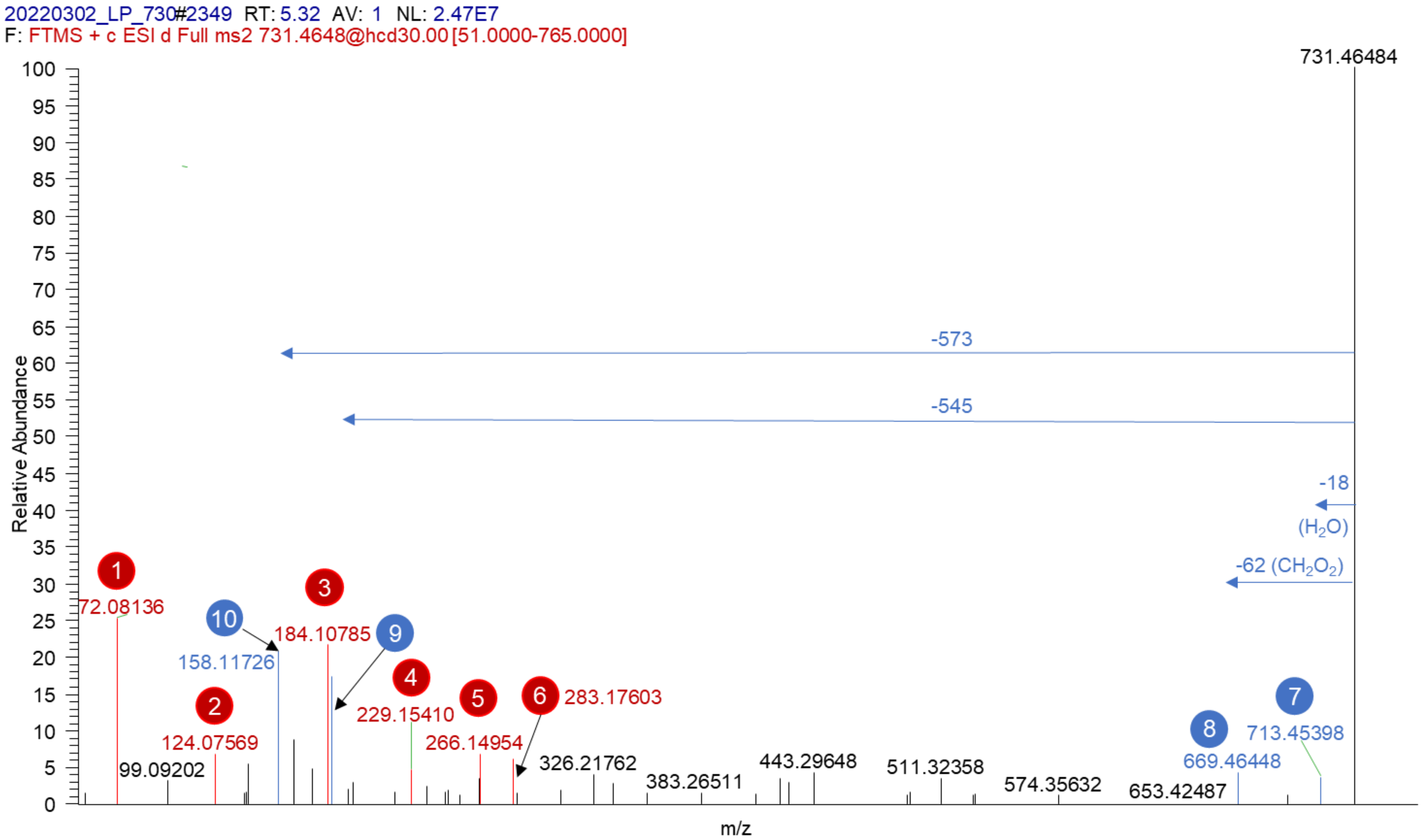

Supplementary Figure 55. MS/MS spectrum of pachycephalamide A (1). Fragments 1-10 matched predicted fragments by MassFrontier. The mechanisms for the formation of these fragments are visualized in Supplementary Figure 54 and Supplementary Figure 53.

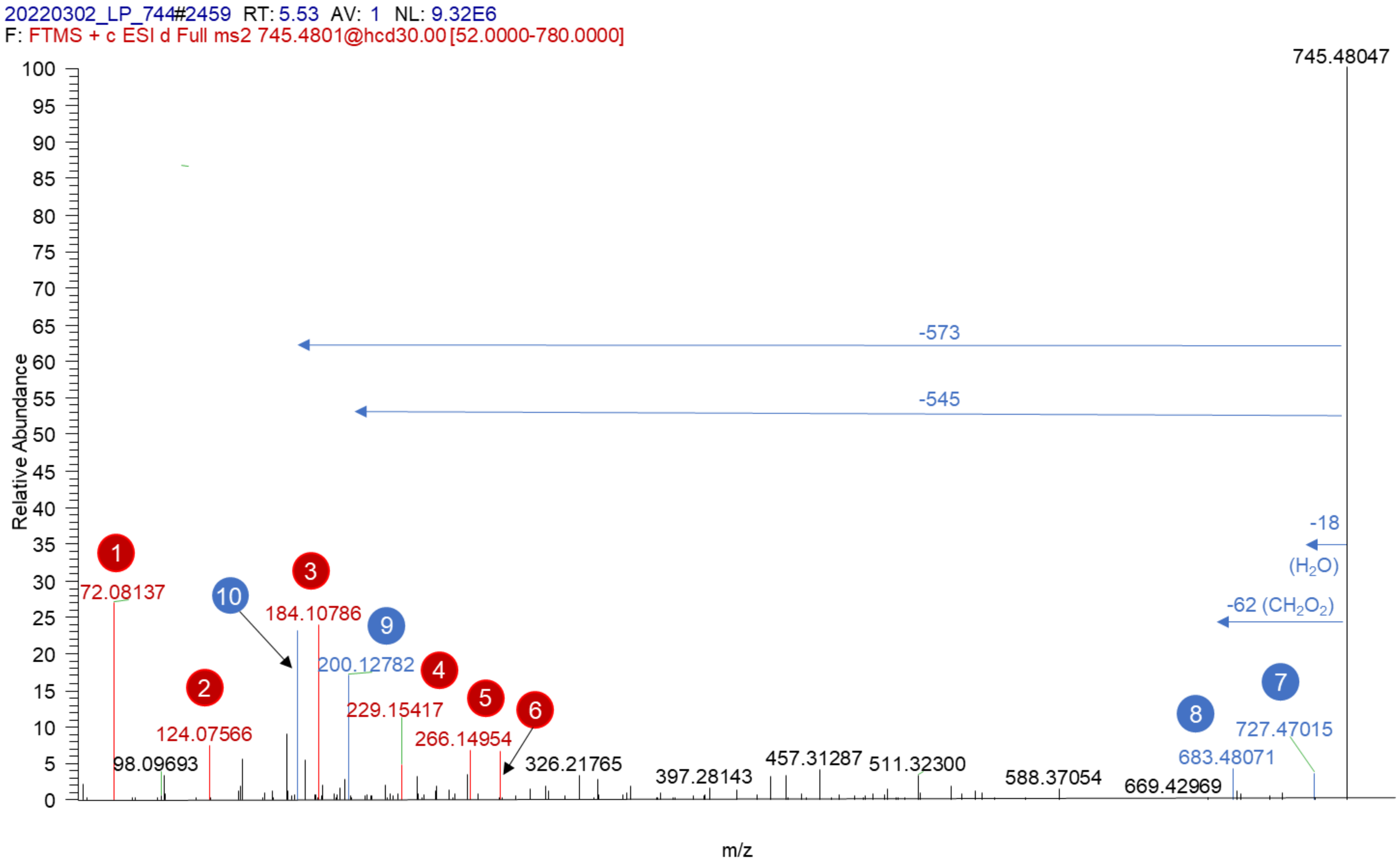

Supplementary Figure 56. MS/MS spectrum of pachycephalamide B (2). Fragments 1-10 matched predicted fragments by MassFrontier. The mechanisms for the formation of these fragments are visualized in Supplementary Figure 54 and Supplementary Figure 53.

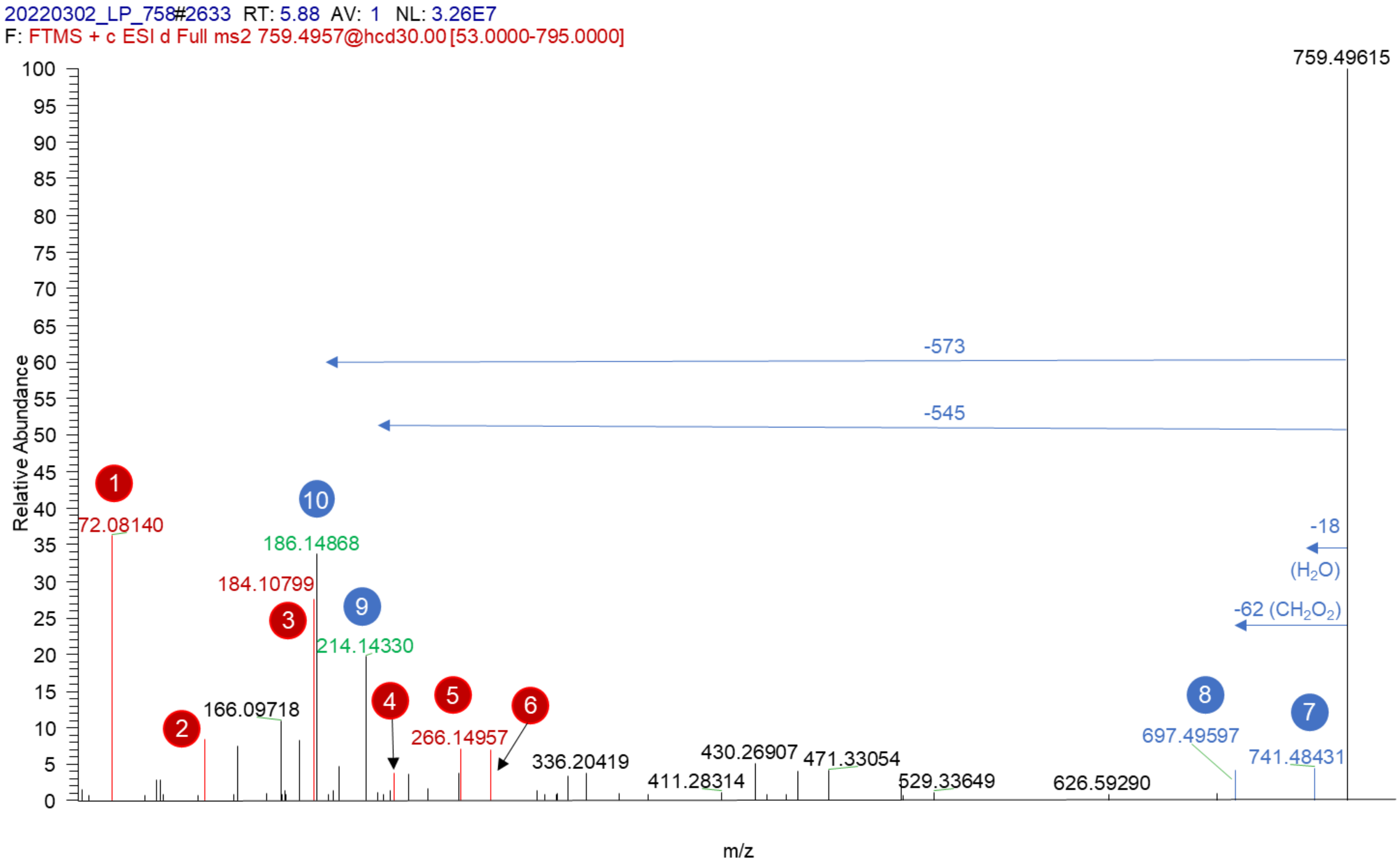

Supplementary Figure 57. MS/MS spectrum of pachycephalamide C (3). Fragments 1-10 matched predicted fragments by MassFrontier. The mechanisms for the formation of these fragments are visualized in Supplementary Figure 54 and Supplementary Figure 53.

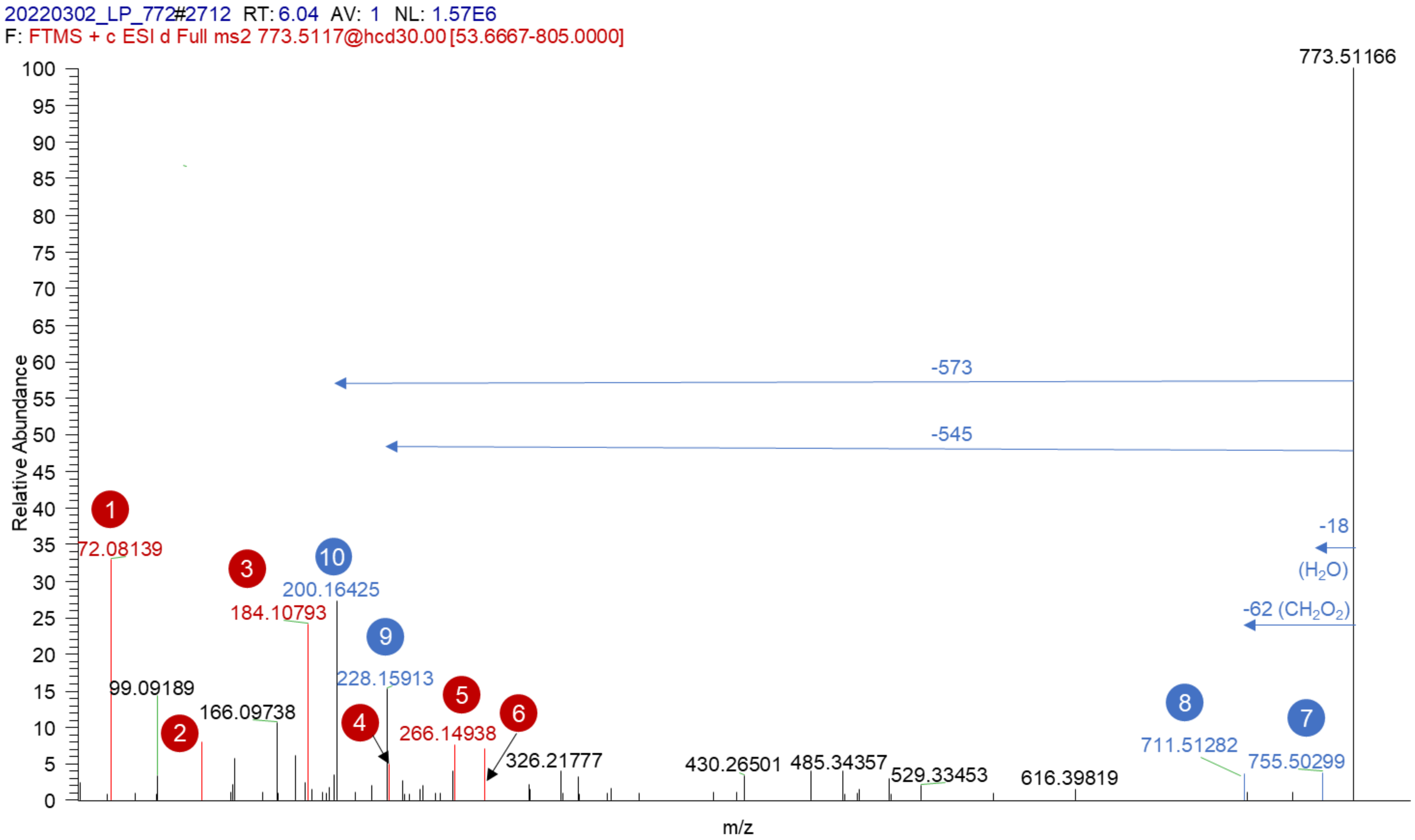

Supplementary Figure 58. MS/MS spectrum of pachycephalamide D (4). Fragments 1-10 matched predicted fragments by MassFrontier. The mechanisms for the formation of these fragments are visualized in Supplementary Figure 54 and Supplementary Figure 53.

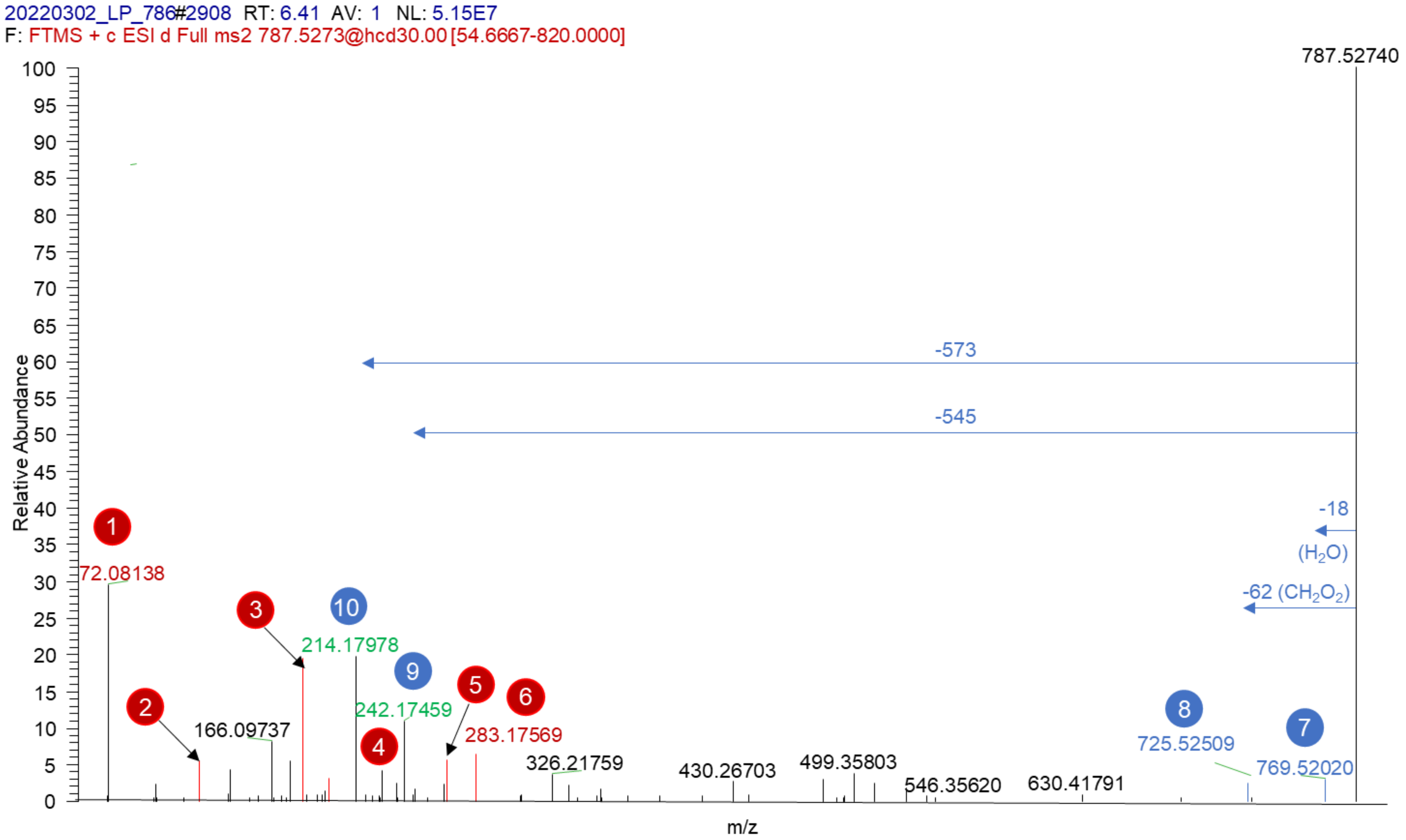

Supplementary Figure 59. MS/MS spectrum of pachycephalamide E (5). Fragments 1-10 matched predicted fragments by MassFrontier. The mechanisms for the formation of these fragments are visualized in Supplementary Figure 54 and Supplementary Figure 53.

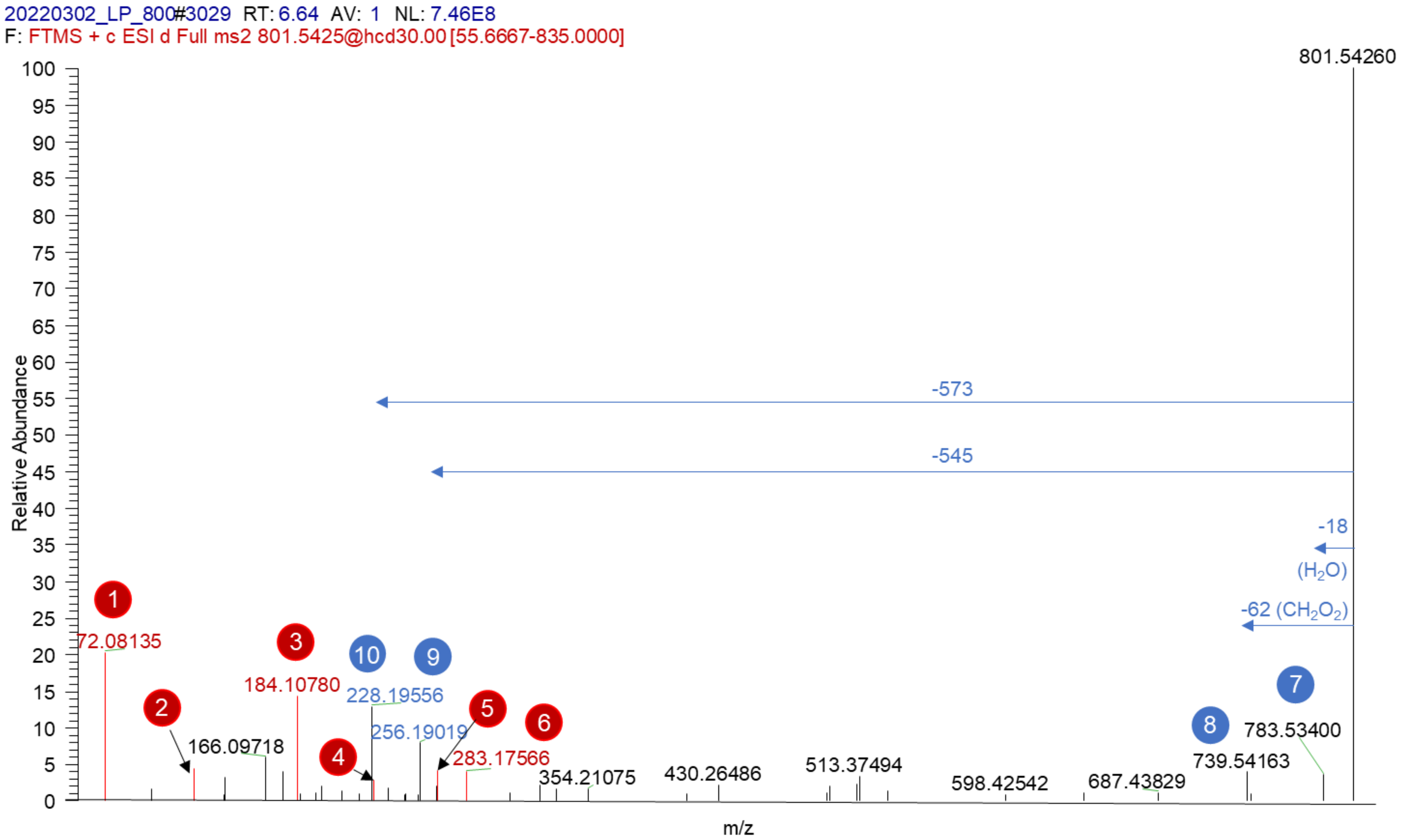

Supplementary Figure 60. MS/MS spectrum of pachycephalamide F (6). Fragments 1-10 matched predicted fragments by MassFrontier. The mechanisms for the formation of these fragments are visualized in Supplementary Figure 54 and Supplementary Figure 53.

Determination of the absolute configuration of the amino acids in pachycephalamide A and demiguisin

Pachycephalamide A (**1**, 1 mg) was hydrolyzed in 2 mL of 6N HCl at 120°C for an hour and the reaction vial was cooled in a bucket of iced water precipitously. Then the reaction solvent was evaporated *in vacuo* to dryness with a rotary evaporator. After lyophilizing the hydrolysate for 6 hours, the free amino acids including an alanine, a valine, a leucine, and arginine in the hydrolysate of **1** were dissolved in 1 N $NaHCO_3$ (100 μL). To the vial containing the free amino acids, 50 μL of either 10 mg/mL L-FDAA (1-fluoro-2,4-dinitrophenyl-5-l-alanine amide) in acetone was added. The reaction mixture was incubated at 80 °C for 10 mins. A 50 μL aliquot of 2 N HCl was added to neutralize the reaction. A 5 μL aliquot of the reaction mixture was analyzed by LC-MS with a gradient solvent system (10% to 40% CH_3CN containing 0.1% formic acid over 40 min, C18 reversed-phase column 100 × 4.6 mm, UV at 340 nm). The same reaction was performed for standard L- and D-Ala, Val, Leu, and Arg. The L-FDAA derivatives of the hydrolysate eluted at the same time as standard l-Ala, Val, Leu, and Arg-FDAA derivatives establishing the absolute configurations of the amino acid residues in **1** as L (elution order: L→ D for Ala, Val, Leu, and Arg) (Supplementary Table 9).

In the same way as above, we proceeded with the Marfey's reaction for demiguisin (**7**). When piperazic acid is in the *S*-configuration, the D-FDAA derivatives elute before the L-FDAA derivatives (Supplementary Table 10, Supplementary Figure 61). The D-FDAA adducts of the piperazic acid derivative units eluted faster than the L-FDAA derivative in the LC/MS chromatogram confirming that the piperazic acid derivatives in demiguisin were in the *S*-configuration and all amino acids were determined to be L-form (Elution order: L→D for Leu, D→L for β,γ-OH-Pip, Pip, DHPA, and γ-c-Pro).[24]

Supplementary Table 9. Elution times of amino acid-FDAA adducts after Marfey reaction for pachycephalamide A (**1**).

Amino acid (AA)	AA+L-FDAA (min)	AA+D-FDAA (min)	Elution order
Leu	19.2	21.7	L->D
Ala	12.7	14.6	L->D
Arg	10.2	10.3	L->D
Val-1	16.5	19.2	L->D
Val-2	16.5	19.2	L->D

Supplementary Table 10. Elution times of amino acid-FDAA adducts after Marfey reaction for demiguisin (**7**).

Amino acid (AA)	AA+L-FDAA (min)	AA+D-FDAA (min)	Elution order
1,2,5,6-tetrahydropyridine-2-carboxylic acid	24.0	23.2	D->L
piperazic acid	21.2	18.3	D->L
leucine	29.0	31.1	L->D
pyrrolidine-2,4-dicarboxylic acid	17.4	16.1	D->L
4,5-dihydroxyhexahydropyridazine-3-carboxylic acid	19.3	18.4	D->L

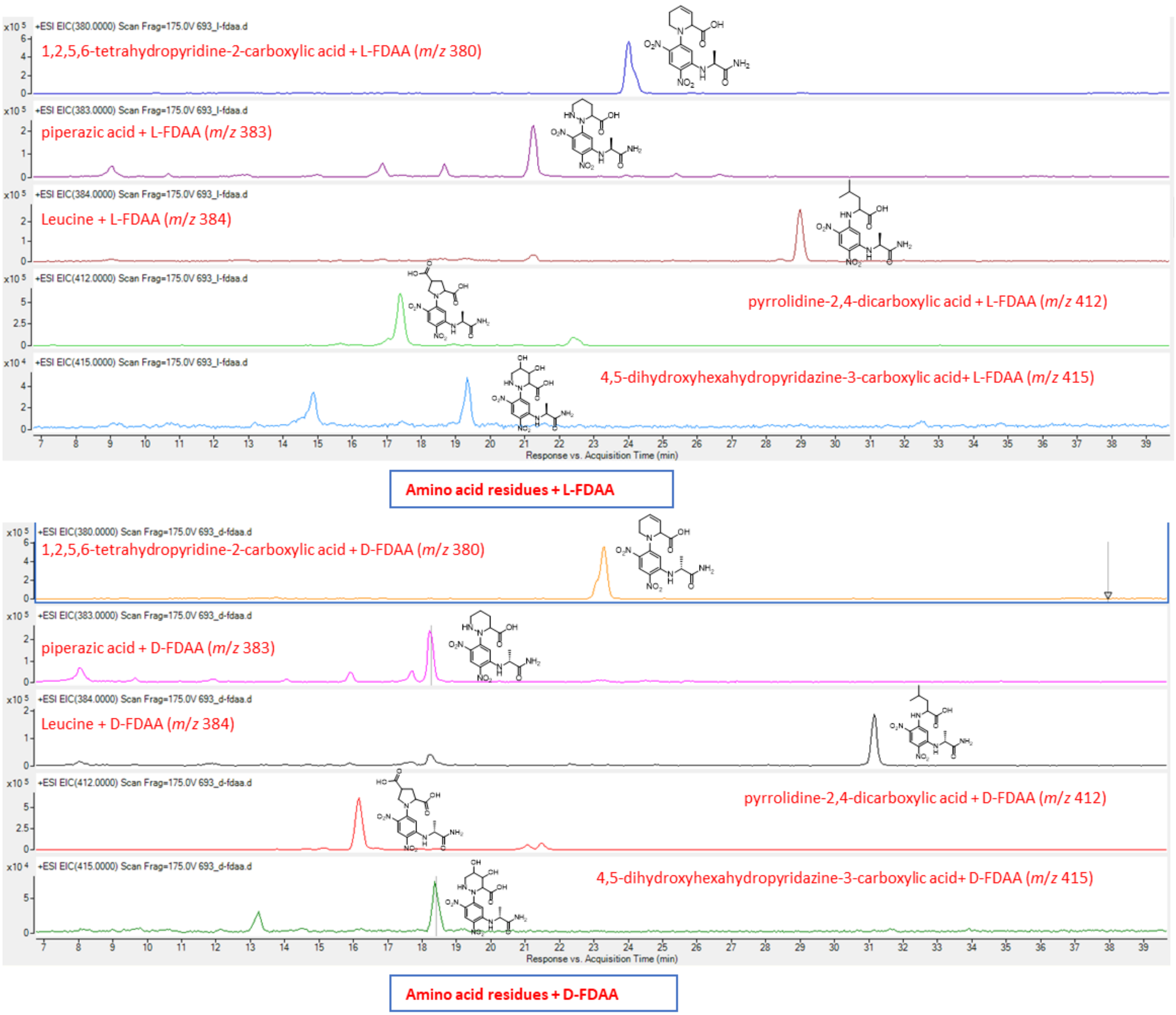

Supplementary Figure 61. Determination of the absolute configuration of the amino acids in demiguisin (**7**) using Marfeys reaction.

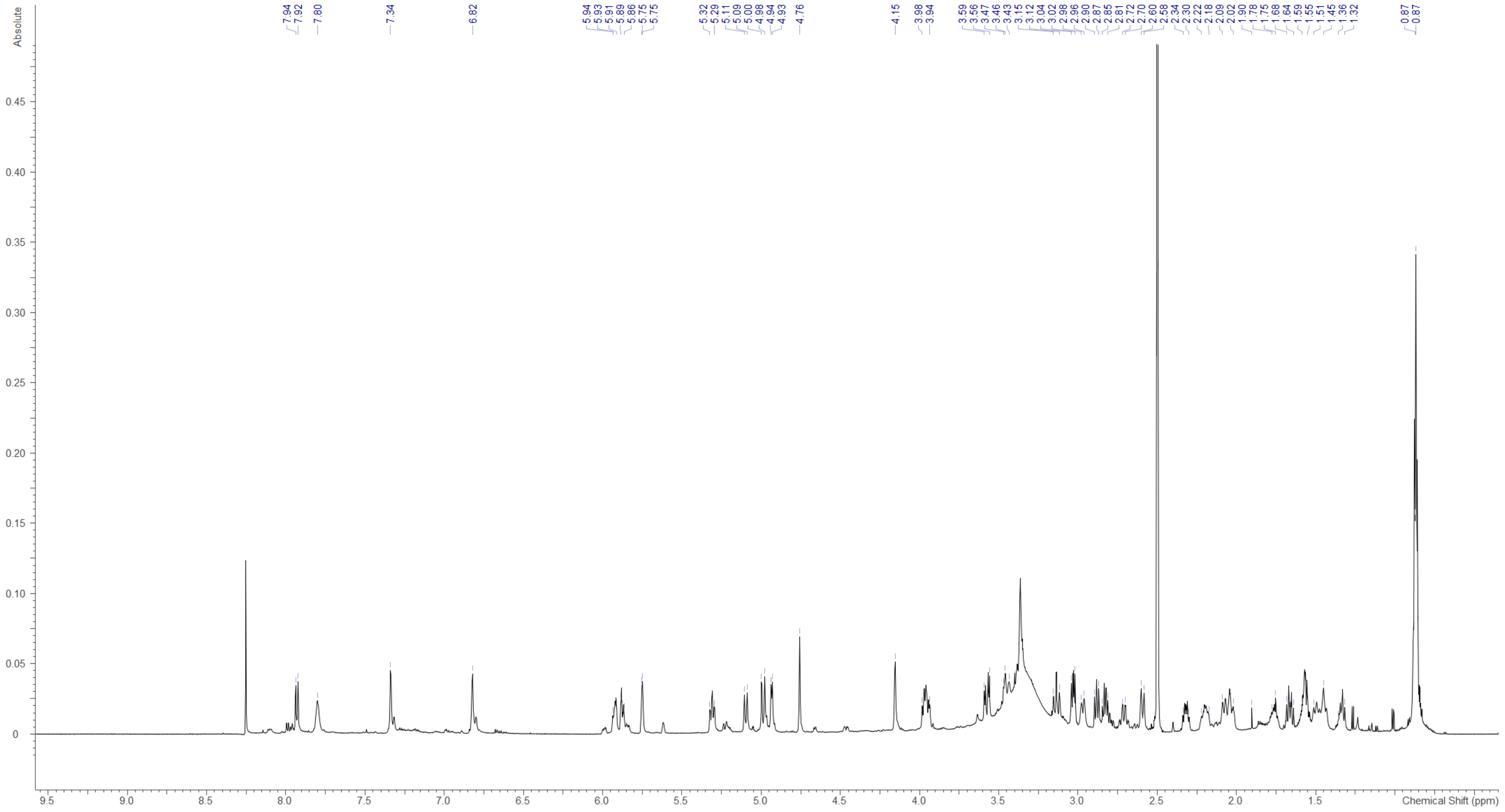

Supplementary Figure 62. ^{1}H NMR spectrum of demiguisin (**7**) in DMSO-d_6.

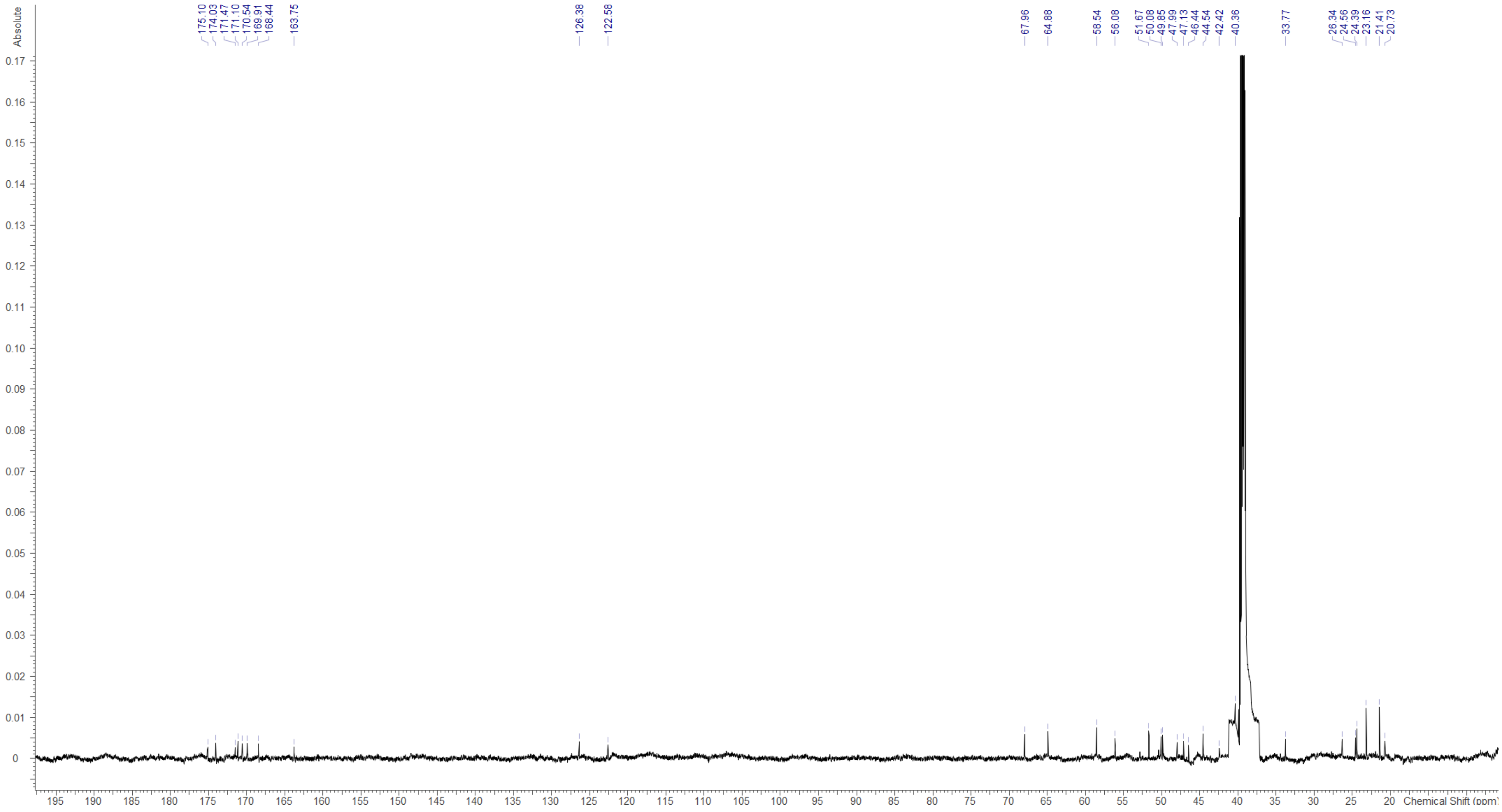

Supplementary Figure 63. ^{13}C NMR spectrum of demiguisin (7) in DMSO-d_6.

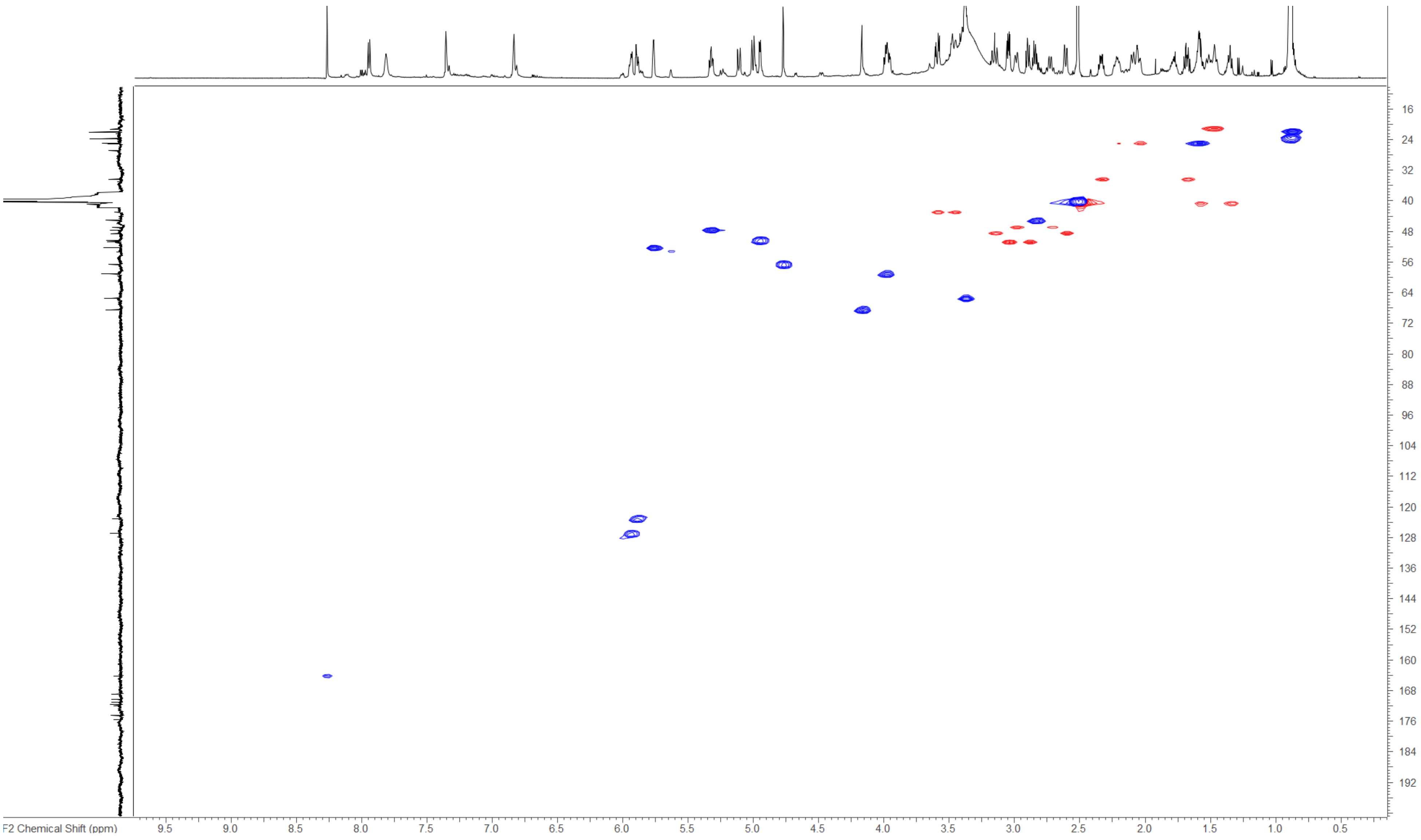

Supplementary Figure 64. HSQC NMR spectrum of demiguisin (**7**) in DMSO-d_6.

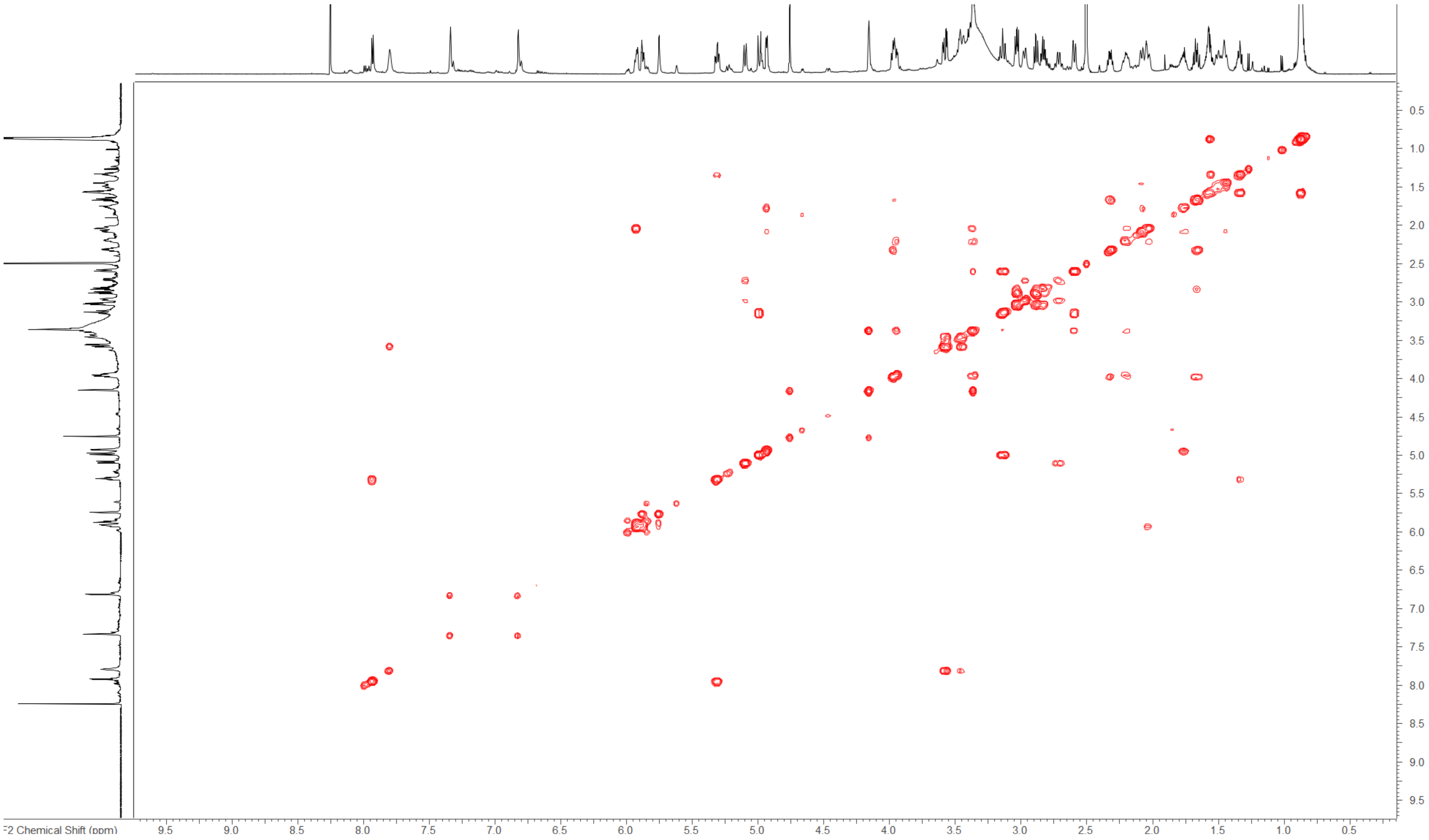

Supplementary Figure 65. COSY spectrum of demiguisin (**7**) in DMSO-d_6.

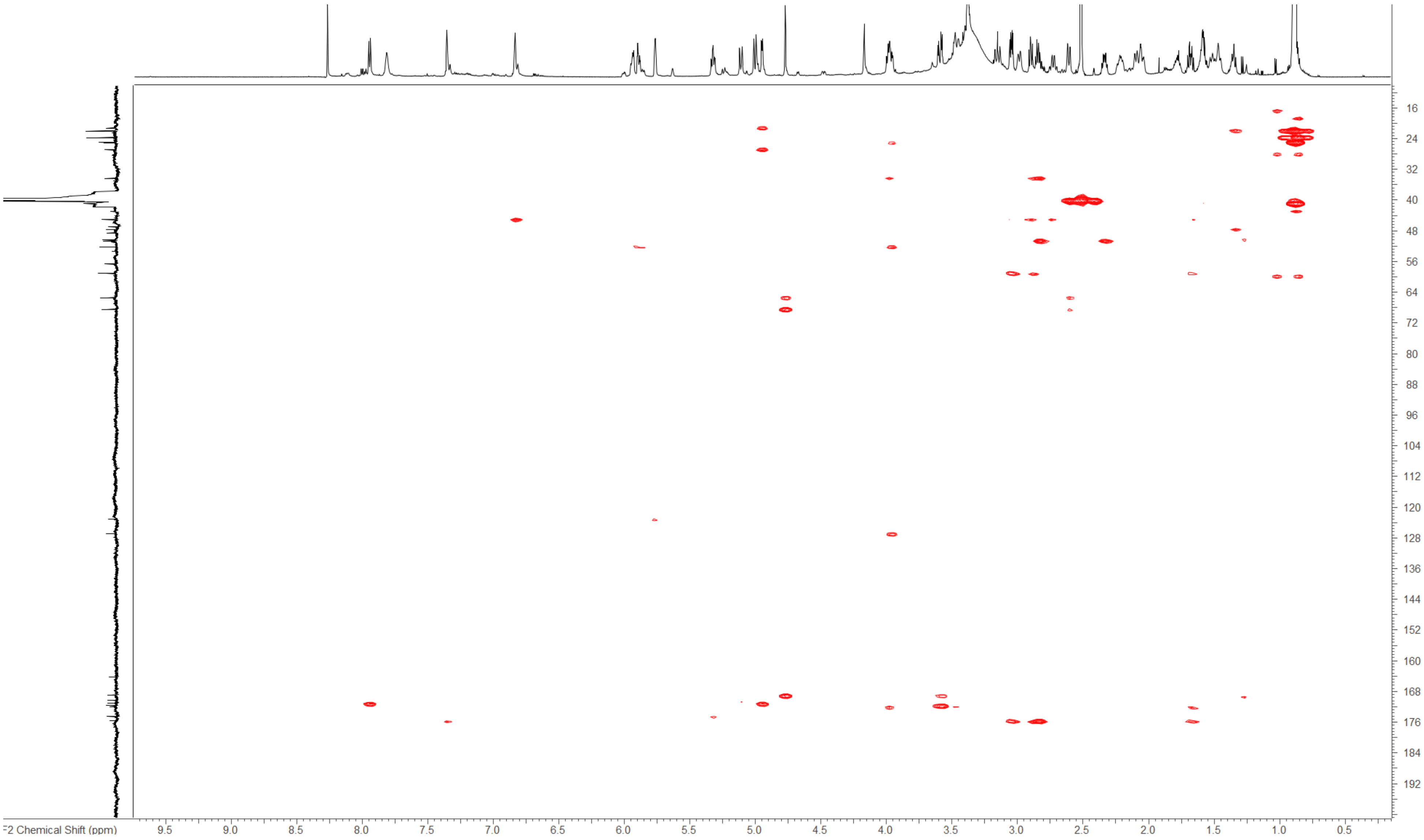

Supplementary Figure 66. HMBC NMR spectrum of demiguisin (7) in DMSO-d_6.

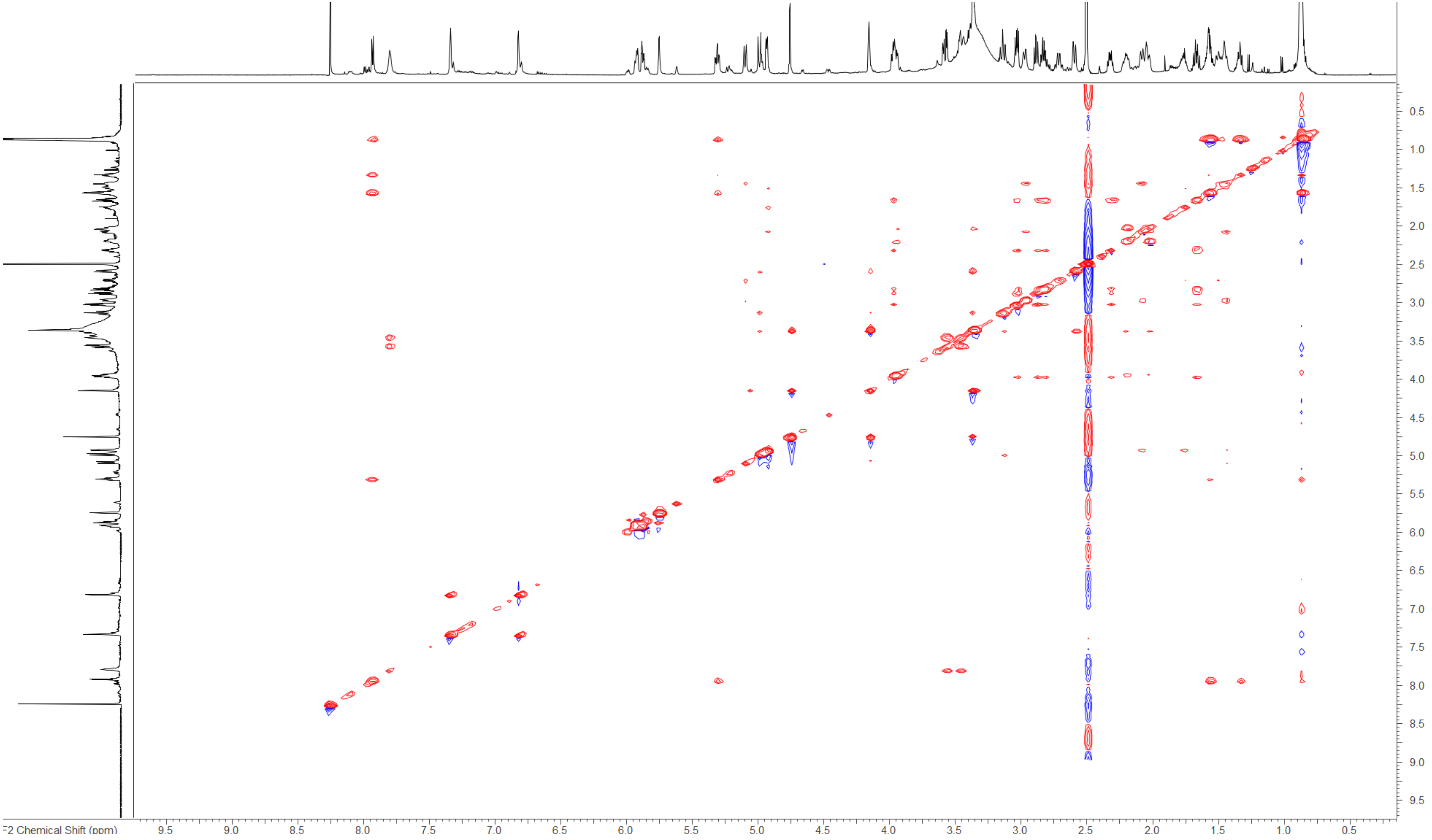

Supplementary Figure 67. TOCSY NMR spectrum of demiguisin (**7**) in DMSO-d_6.

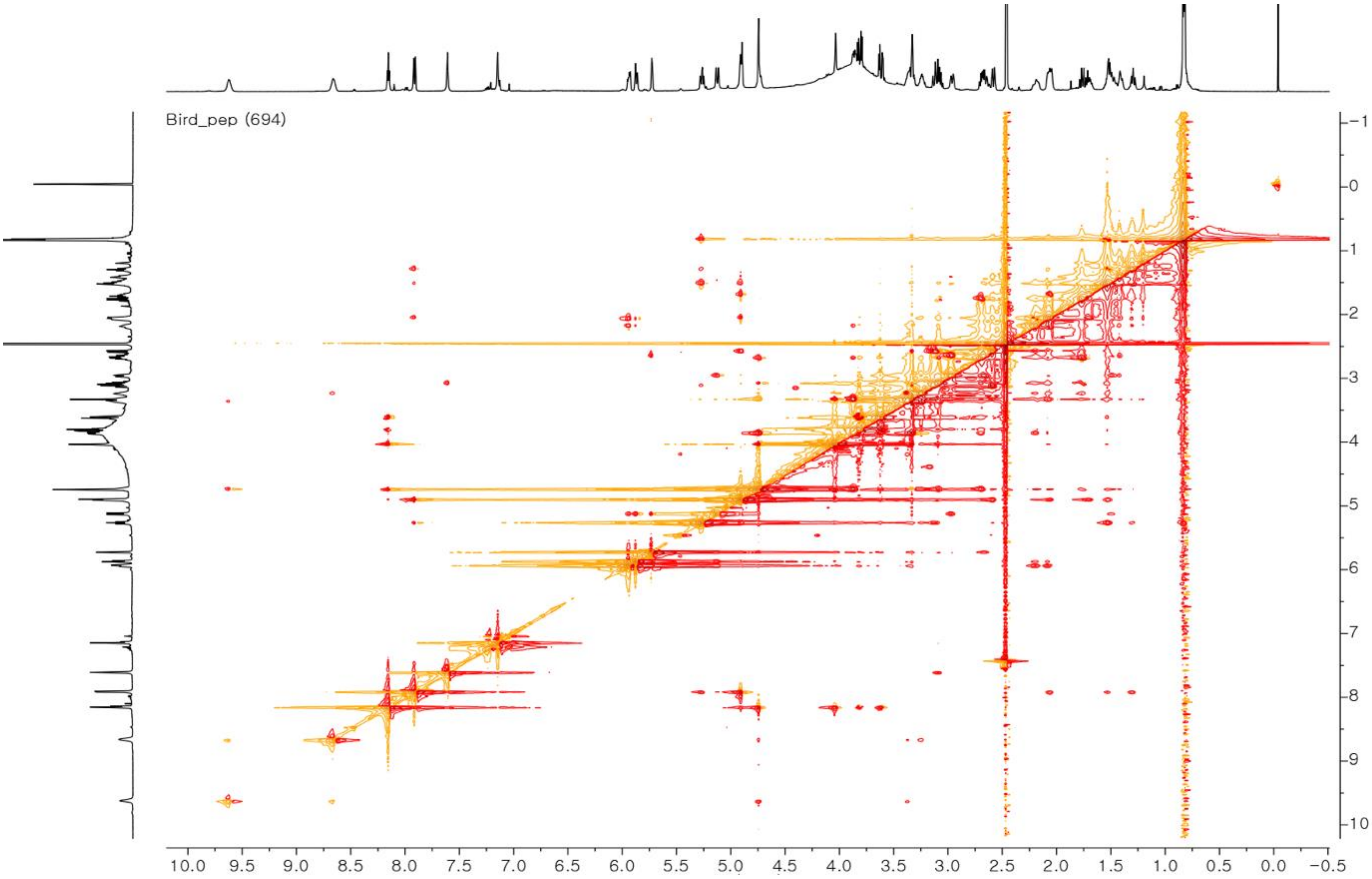

Supplementary Figure 68. ROESY NMR spectrum of demiguisin (**7**) in DMSO-d_6 (600 Mz, without FA).

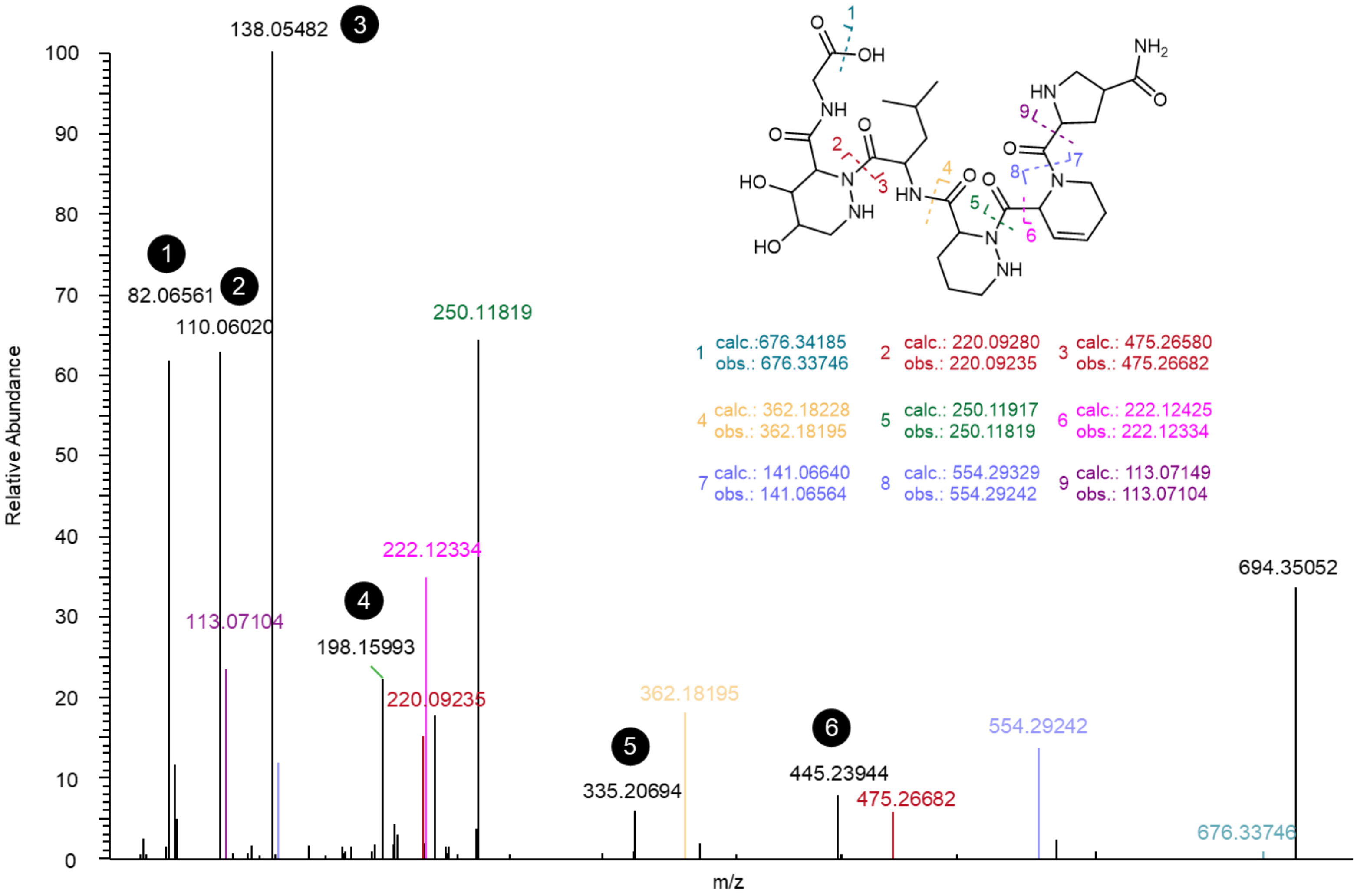

Supplementary Figure 69. MS/MS spectrum of demiguisin (7). Fragments resulting from peptide cleavage and matching calculated m/z values are highlighted. Fragments which matched the prediction of MassFrontier 8.0 are marked with black circles (**1**-**6**). The predicted mechanisms for the formation of these fragments are visualized in Supplementary Figure 70.

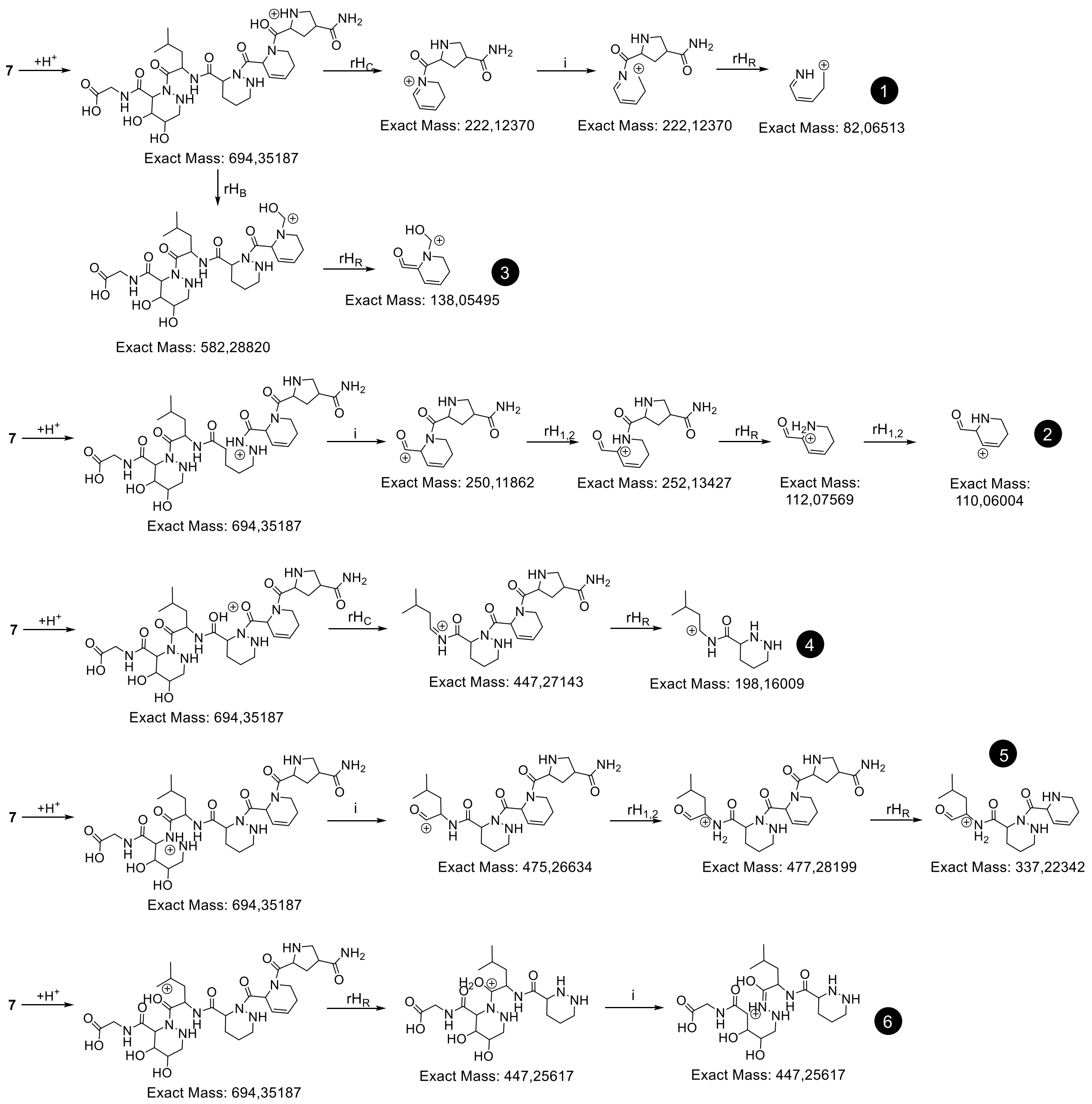

Supplementary Figure 70. Mechanisms of MS/MS fragment formation for demiguisin (7). Mechanism for fragments matching calculated m/z values were predicted using MassFrontier 8.0. Fragments which matched the prediction of MassFrontier 8.0 are marked with black circles (1-6).

Detection of peptides in bird feathers.

The weight of feathers was determined in clean (unused) glass vials, in which the feathers were extracted with 1 mL MeOH overnight. The samples were filtered, and the raw extracts were dried under reduced pressure. Samples were resuspended in 400 µl MeOH using ultrasonication and remaining particles were removed by centrifugation. Samples were then subjected to liquid chromatography tandem high-resolution mass spectrometry (LC-HRMS/MS) analysis (using blank samples and isolated compounds as standard).

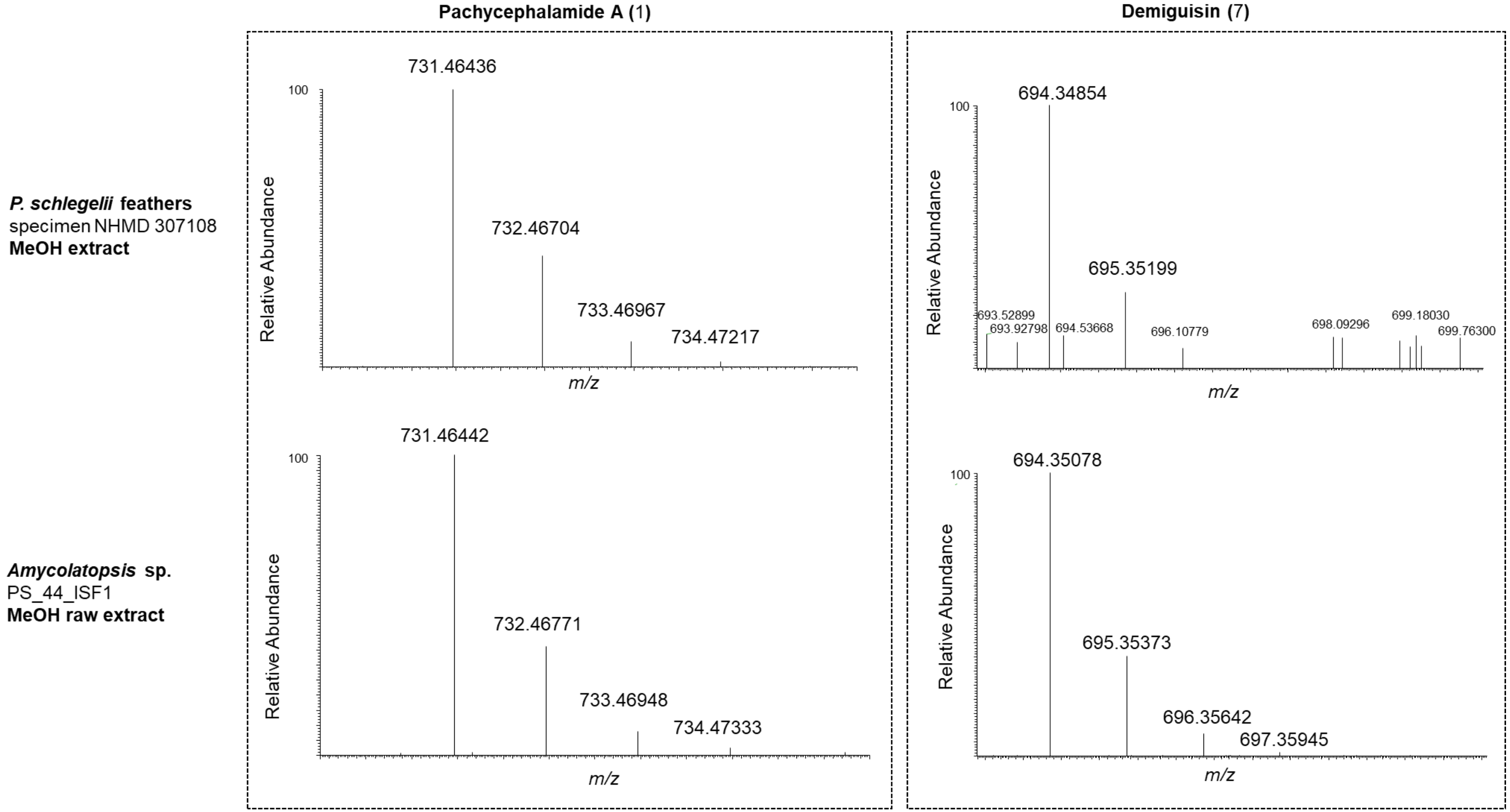

Supplementary Figure 71. Comparison of MS/MS spectra of pachycephalamide A and demiguisin detected in extracts of *P. schlegelii* feather extracts and a PS_44_ISF1 culture ((n=3).

Supplementary Table 11. Overview of biosynthetic gene cluster predicted by antiSMASH Version 7.0 beta. Regions included in the transcriptomic analysis are highlighted in grey. For detailed analysis, see Supplementary Data 1.

Scaf-fold	Region	Type (number of PKS/ NRPS modules/ domains)	A domain specificities	From	To	Most similar known cluster		similarity
**1**	R1.1	T1PKS (10 PKS modules)	-	517,711	607,815	rifamorpholines A-E	Polyketide	57%
	R1.2	NRPS, other, oligosaccharide, T2PKS (3 A domains, 3 C domains, iterative aromatic KS)	**Leu**, asn, haorn	875,369	977,873	arixanthomycin A-C	Polyketide Type II	57%
	R1.3	lanthipeptide class III		1,001,272	1,023,815			
	R1.4	ectoine		1,979,244	1,989,633	ectoine	ectoine	100%
	R1.5	T1PKS (1 PKS module, 1 A domain, 1 C domain)	**Val**	2,781,460	2,824,347	toxoflavin/fervenulin	Other	7%
	R1.6	T1PKS, butyrolactone (10 PKS modules, 2 A domains)	**Ile, Val**	2,850,232	2,955,978	macrotermycins	Polyketide	96%
	R1.7	NRPS, nucleoside (2 A domains, 1 C domain)	**Gly**, Gln	3,059,532	3,104,460	toyocamycin	other	30%
**2**	R2.1	T1PKS (17 PKS modules)		86,698	208,478	notonesomycin A	other	15%
	R2.2	terpene		223,002	243,065	isorenieratene	terpene	71%
	R2.3	NRPS (3 A domains, 1 C domain)	Phe, **Leu**, Bht,	263,583	307,566	chloramphenicol	NRP	11%
	R2.4	NRPS (8 A domains, 7 C domains)	Ser, Phe, Gln, Orn, Ser, **Gly**, Ser	828,765	890,377	bosamycin A-F	NRP	22%
	R2.5	T1PKS, hglE-KS (2 PKS modules)		906,826	957,226	hexacosalactone A	other	9%
	R2.6*	NRPS, terpene, T1PK, lanthipeptide class II, thioamitides (NRPS1: 10 A domains, 9 C domains; PKS: 9 PKS modules, NRPS2: 3 A domains, 2 C domains)	NRPS1: Orn, Thr, **Arg, Val**, Tyr NRPS2: fohOrn, **Ala**, ohOrn	967,183	1,167,977	coelichelin	NRP	63%
	R2.7*	NRPS (4 A domains, 5 C domains, 1 hybrid-KS)	**Arg, Leu**, **Ala**, Thr	1,228,532	1,278,733	daptomycin	NRP	18%
	R2.8	indole		1,336,396	1,357,478	franciamycin	Polyketide	14%
	R2.9	terpene		1,793,851	1,814,241	2-methylisoborneol	terpene	100%
	R2.10	NAPAA (1 A domain)	Tyr	2,017,758	2,049,421	ε-Poly-L-lysine	NRP	100%
	R2.11	NAPAA, NRPS, ladderane (6 A domains, 4 C domains, 4 iterative type II polyene KS)	**Val**, Trp, Ser, **Arg**, Gln, Asn	2,059,913	2,141,646	kitacinnamycin A-F	NRP	9%
	R2.12	terpene		2,158,716	2,177,866	SF2575	Polyketide Type II	6%
	R2.13	NRPS-independent siderophore		2,289,056	2,300,849	nonactin	Polyketide	33%
	R2.14	terpene		2,579,927	2,600,865	isorenieratene	terpene	37%
**3**	R3.1	CDPS		72,653	92,019			
	R3.2*	T1PKS, NRPS, blactam (6 A domains, 5 C domains, 1 PKS module)	Trp, **Pip, Pro, Pro, Leu, Gly**)	254,499	346,062	sanglifehrin A	NRP+ Polyketide	18%
	R3.3	CDPS		387,017	407,700	A83543A	Polyketide	8%
	R3.4	NRPS (4 A domains, 2 C domains)	Phe, Asn, Ser, Phe	601,462	644,359			
**4**	R4.1	NAPAA (1 A domain)	Lys	237,809	271,717	ε-Poly-L-lysine	NRP	100%
	R4.2	T1PKS (1 PKS module)		366,931	411,160			
**5**	R5.1	terpene		82,905	105,151	geosmin	terpene	100%
	R5.2	NRPS (8 A domains, 4 C domains)	**Ala**, Gln, Asn, Phe, Trp, Tyr, Asn	192,171	257,817	rotihibin A	NRP	21%
**10**	R10.1	T1PKS (2 PKS modules)		1	16,751	sipanmycin	Polyketide	19%

Phylogenetic analysis of biosynthetic enzymes

For phylogenetic reconstruction of condensation and ketosynthase domains reference sequences were retrieved from natural product domain seeker database (NaPDoS2_v13b). PS_44_ISF1 condensation domains were retrieved by uploading the genome in the NaPDoS2 server (in total 46).[25] PS_44_ISF1 and reference sequences were aligned using the ClustalW multiple alignment tool with default parameters.[26] Afterwards, an approximately-maximum-likelihood phylogenetic tree was inferred using the FASTTREE tool using default parameters.[27] Both tools were used on the Galaxy platform.[28] For the classification of ketosynthase domains of specific BGCs, domain sequences were retrieved from antiSMASH outputs.[29] Then KS sequences were compared to reference sequences and phylogenetic trees were generated using the NaPDoS2 server.[30] Phylogenetic trees were visualized using FigTree v1.4.4 (http://tree.bio.ed.ac.uk/software/figtree/).

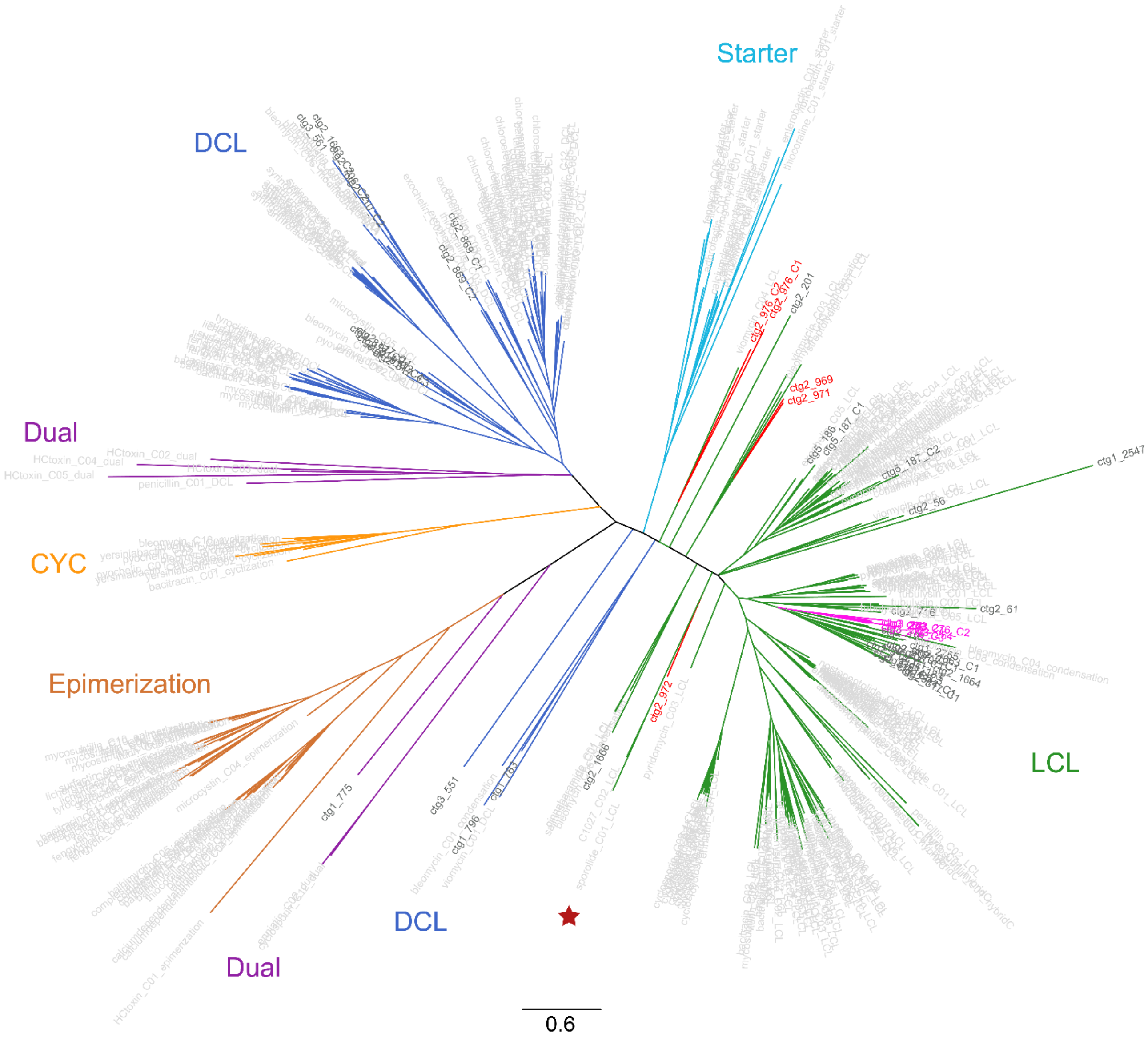

Supplementary Figure 72. Phylogenetic analysis of NRPS-type condensation (C) domains encoded in the genome of *Amycolatopsis* sp. PS_44_ISF1. Maximum Likelihood tree based on ClustalW multiple sequence alignment of condensation domains from *Amycolatopsis* sp. PS_44_ISF1 (dark grey) and reference C domains from the Napdos2 database (light grey, database version = NaPDoS2_v13b). Multiple sequence alignment and phylogenetic reconstruction was performed using the Galaxy server. Red and pink branches mark C domains encoded in the BGCs of lipopeptides and 694, respectively. The red star indicates the C domains related to C1 in sporolide biosynthesis, which catalyzes the condensation of two precursor molecules.

Supplementary Table 12. Substrate specificities of adenylation domains encoded in the *pch* BGC. For detailed pathway analysis, see Supplementary Data 1.

	Stachelhaus code[a]											
domain	1	2	3	4	5	6	7	8	9	10	Proposed substrate	Stachelhaus code match
PchP1-A1	D	V	E	N	V	G	A	I	T	K	Arg	70%
PchP3-A1	D	A	W	F	L	G	N	V	V	K	Leu	100%
PchP4-A1	D	V	W	H	L	S	L	I	E	K	Ser	90%
PchP7-A2	D	F	W	N	I	G	M	V	F	K	Thr	90%

[a] Stachelhaus codes were extracted according to Stachelhaus et al. (1999)[46] after pairwise alignment with GrsA (NCBI Accession number: WP_043070050.1).

Supplementary Table 13. Substrate specificities of adenylation domains encoded within the *dmg* pathway for demiguisin. For detailed pathway analysis, see Supplementary Data 1.

	Stachelhaus code[a]											
domain	1	2	3	4	5	6	7	8	9	10	Proposed substrate	Stachelhaus code match
DmgP1-A1	D	V	Y	L	V	T	Q	V	V	K	Trp	Piz
DmgP2-A1	D	M	Q	Y	V	A	Q	Q	A	K	Pro/Pip	Pro
DmgP5-A1	D	I	Q	F	Q	A	Q	V	I	K	Pro/Pip	Pip
DmgP5-A2	D	V	Y	L	I	T	Q	V	V	K	Pro/Pip	Piz
DmgP5-A3	D	A	W	F	L	G	H	V	V	K	Leu	90%
DmgP7-A1	D	I	A	Q	W	G	V	I	W	K	Gly	80%

[a] Stachelhaus codes were extracted according to Stachelhaus et al. (1999)[46] after pairwise alignment with GrsA (NCBI Accession number: WP_043070050.1). DmgP1-A1 and DmgP5-A2 have 90% match.

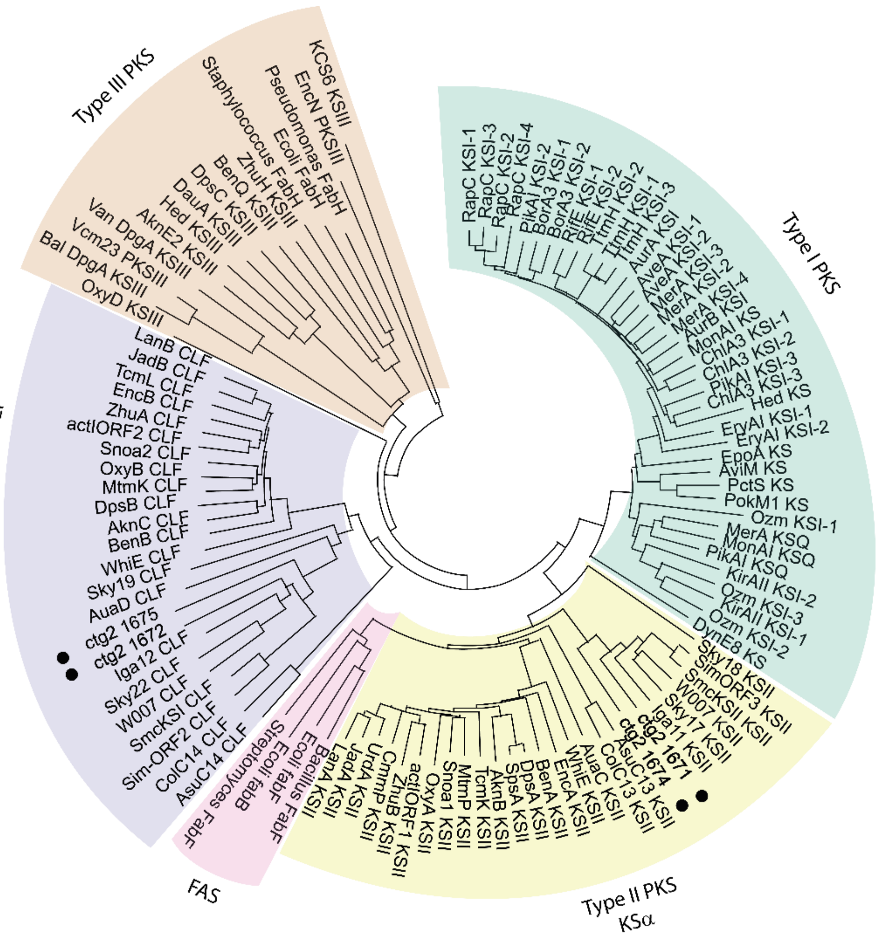

Supplementary Figure 73. Phylogenetic analysis of ketosynthase (KS) domains Ctg2_1671/2 and Ctg2_1674/5. Reference sequences retrieved from Du et al. (2016)[52]. KS domains were aligned using the ClustalW algorithm and the phylogenetic tree was constructed using the build-in neighbor-joining algorithm of Geneious Prime. Positions with black dots represent ketosynthases encoded in PS_44_ISF1.

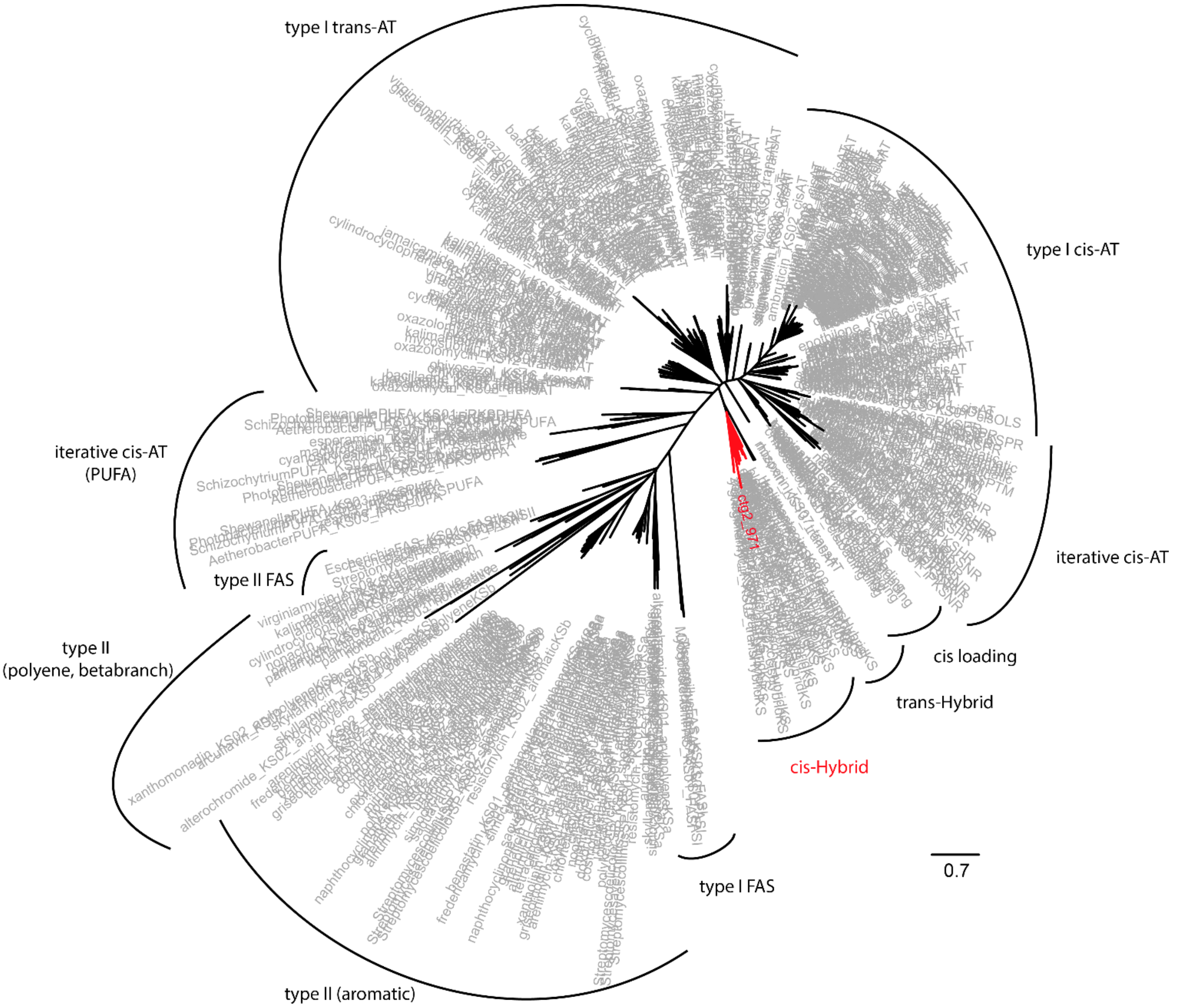

Supplementary Figure 74. Phylogenetic analysis of KS domain PchP3 (ctg2_971) encoded in the *pch* BGC. Reference sequences were retrieved from the Napdos2 database and tree visualized using FigTree v1.4.4. Red branches mark cis-hybrid KS domains.

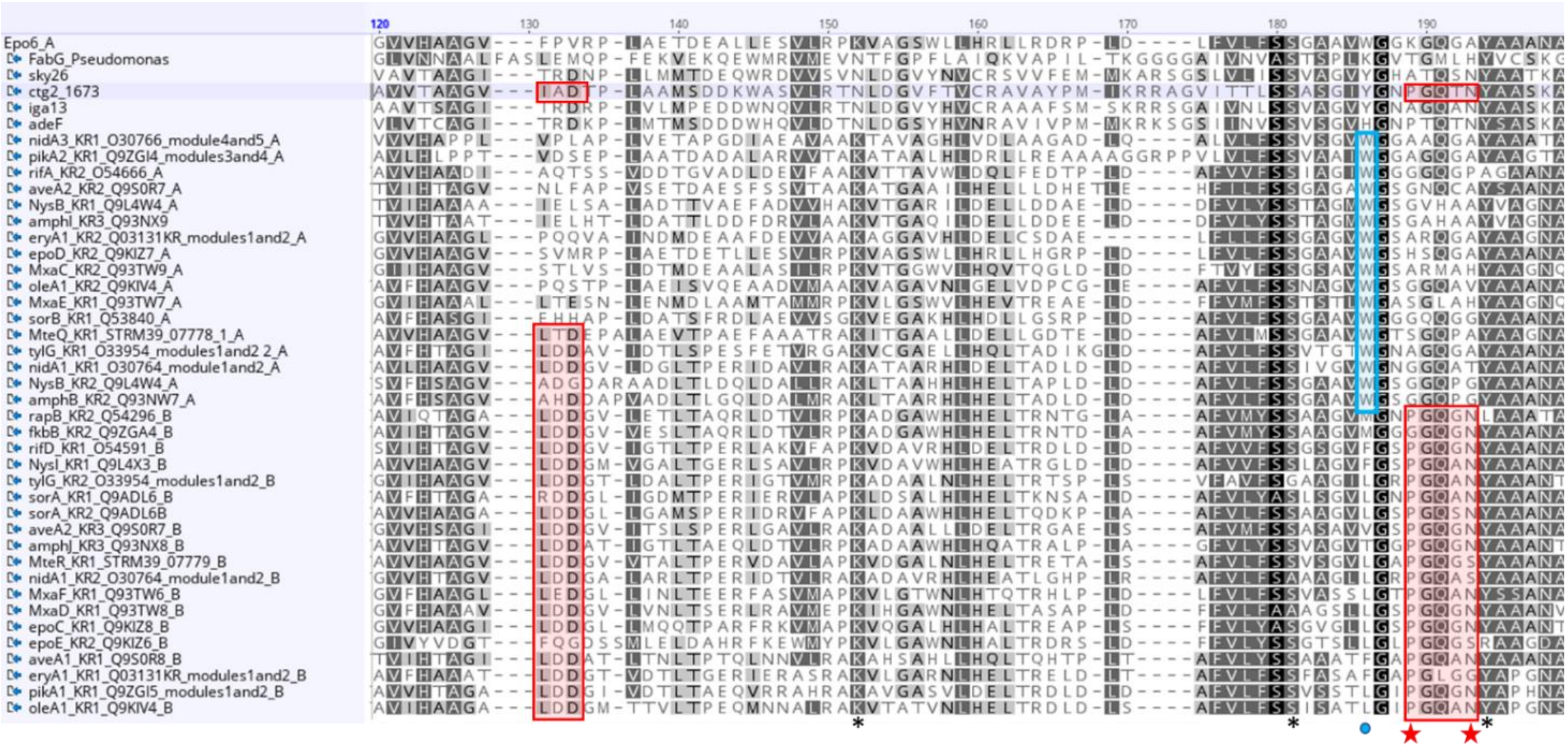

Supplementary Figure 75. Alignment of KR domain of gene cluster region ctg2_1673. Sequences from skyllamyicn, ishigamide, ADEP1 and other A- and B-type ketoreductases from Caffrey et al. (2003).[31] were retrieved (accession numbers in sequence names) and aligned using the built-in ClustalW algorithm of Geneious Prime. The LDD motif and P144/N148 (in this alignment P189/N193, red stars) typical for B-type ketoreductases and the characteristic W141 (here W187) are highlighted in red and blue, respectively. Asterisks (*) mark catalytic residues.

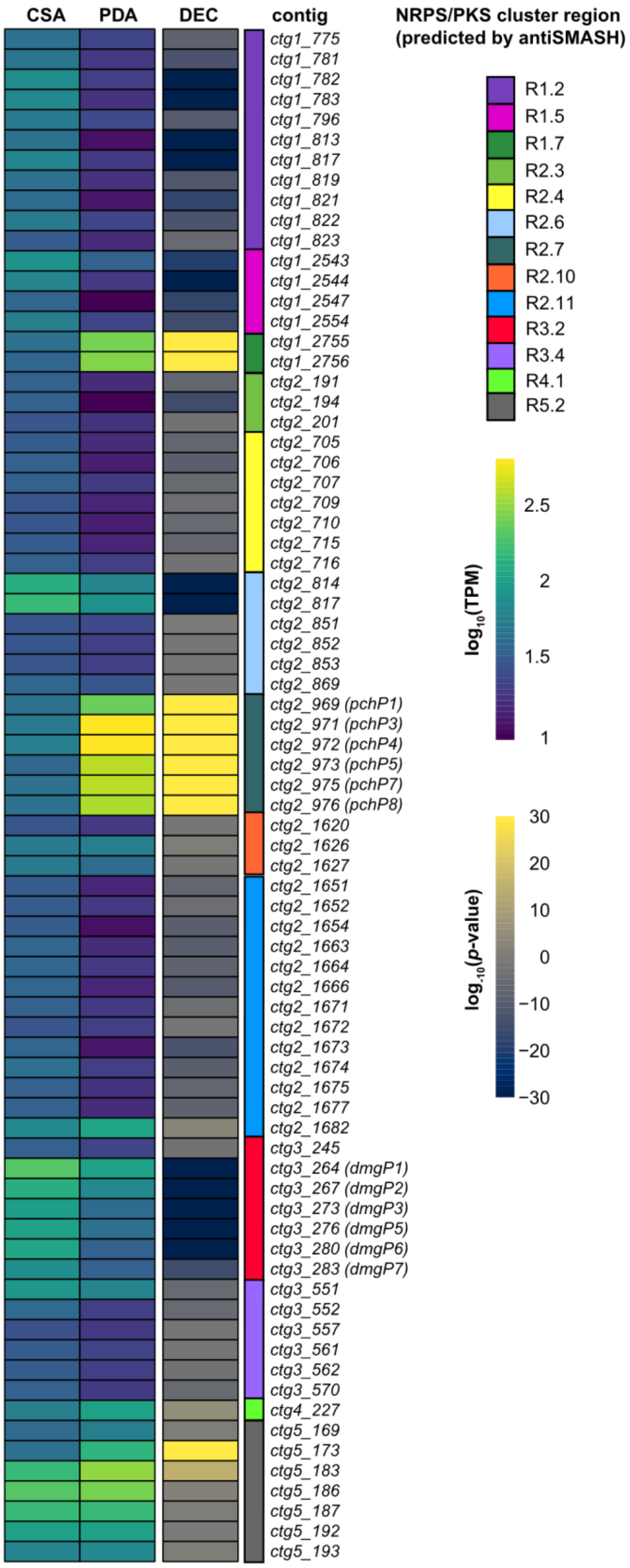

Supplementary Figure 76. Comparative expression analysis (growth on PDA and CSA, n=1) of core biosynthetic genes encoded in NRPS/PKS-containing regions within the PS_44_ISF1 genome. BGC regions were predicted by antiSMASH v.7beta (strict version). Analysis of the differential gene expression when grown on a lipopeptide high-production (PDA) and low-production (CSA) medium (DEC: differential expression confidence; n=1). Source data are provided as a Source Data file.

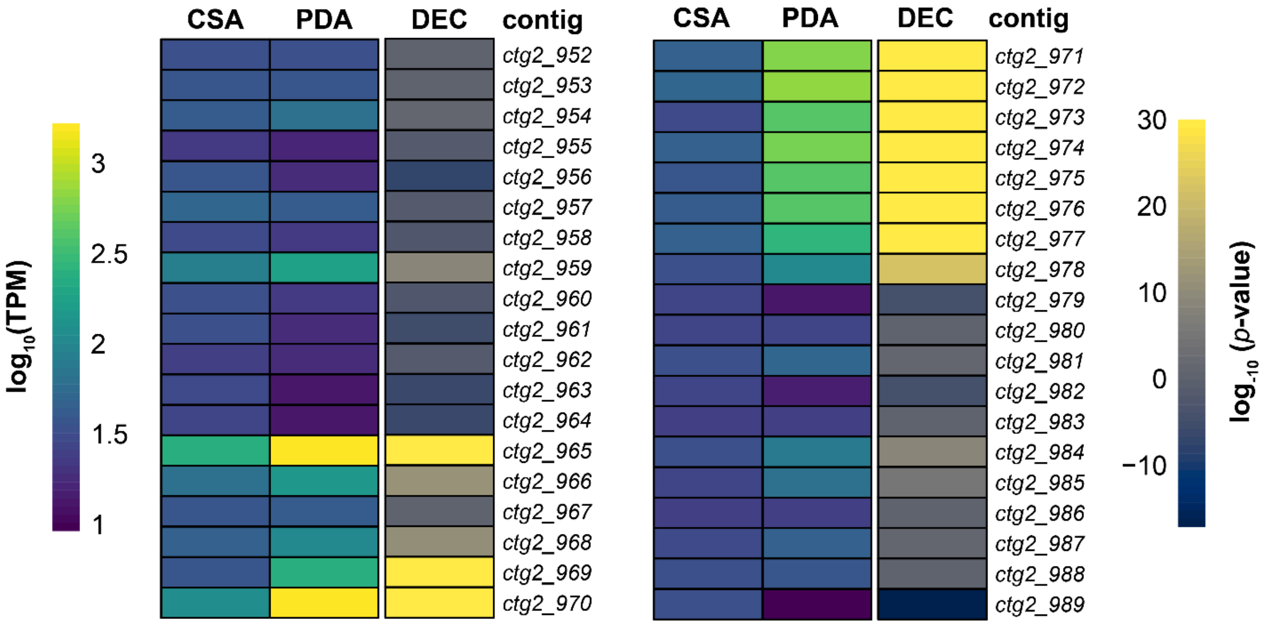

Supplementary Figure 77. Heatmap of the comparative expression analysis of biosynthetic genes in the *pch* BGC (region R2.7). Analysis of the differential gene expression when grown on a lipopeptide high-production (PDA) and low-production (CSA) medium (DEC: differential expression confidence; n=1). The *p*-value corresponds to the probability that, for a given gene, a randomly selected transcript would come from that gene and is calculated as number of transcripts mapped to that gene/total number of transcripts from each sample. The probabilities for each sample are then multiplied to from the *p*-value. Source data are provided as a Source Data file.

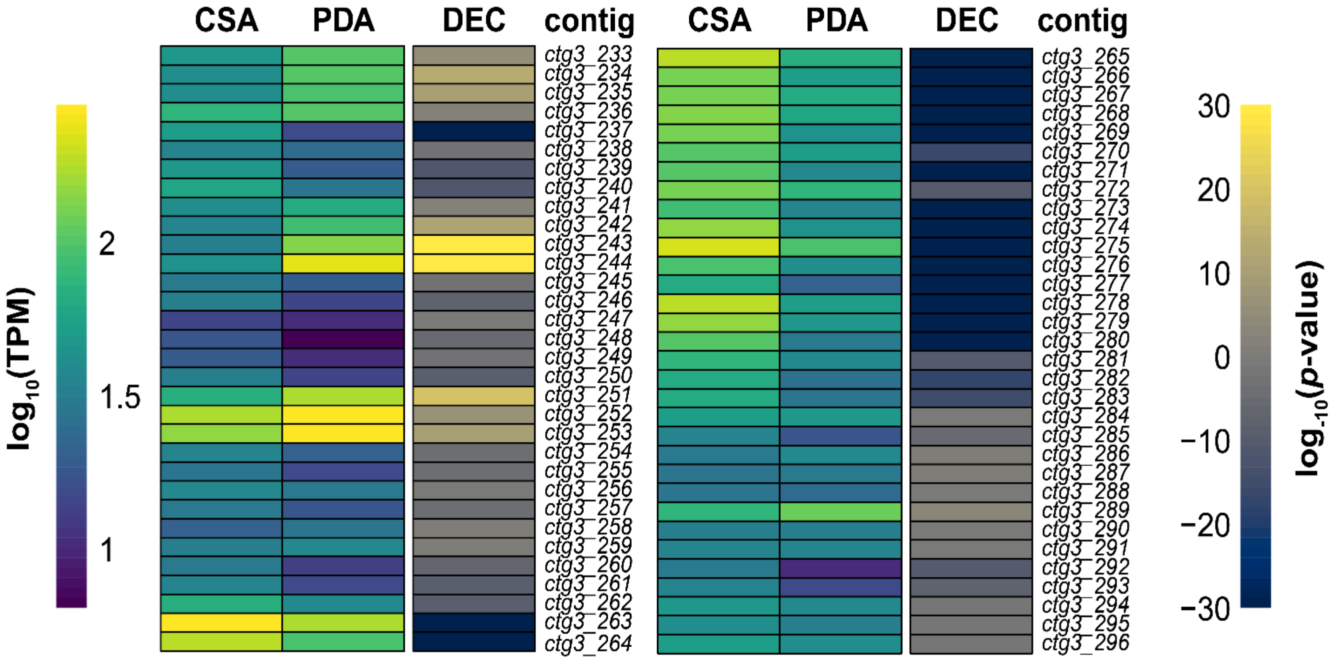

Supplementary Figure 78. Heatmap of the comparative expression analysis of biosynthetic genes in the *dmg* BGC. Analysis of the differential gene expression when grown on a lipopeptide high-production (PDA) and low-production (CSA) medium (DEC: differential expression confidence; n=1). The *p*-value corresponds to the probability that, for a given gene, a randomly selected transcript would come from that gene and is calculated as number of transcripts mapped to that gene/total number of transcripts from each sample. The probabilities for each sample are then multiplied to from the *p*-value. Source data are provided as a Source Data file.

Generation of *Amycolatopsis* sp. PS_44_ISF1 knockout mutants.

Knockout mutants of PS_44_ISF1 were generated via intergeneric conjugational transfer. Methylation-deficient *E. coli* ET12567/pUZ8002 was transformed with pKJ55-derived suicide vector via electroporation. For each vector construct three conjugation were prepared as follows: 10 mL ET12567 (pUZ8002/pKJ55-ko) in LB medium (supplemented with 50 µg/ml Apr, 25µg/ ml Kan, 25 µ/ml Cam) were grown at 37 °C to an OD_{600} of 0.4-0.5, washed twice with ice-cold LB to remove residual antibiotics, and resuspended in fresh 1 mL LB without antibiotics. PS_44_ISF1 spore suspensions (50 µl, $3.5x10^9$ spores/ml, $2x10^8$ spores in 25% glycerol) were added to 500 µl of 2x YT medium and heat shocked at 50 °C for 10 min. Equal amounts (v/v) of donor and recipient cells were mixed and the bacteria were pelleted by centrifugation. The medium was discarded, and the pellets were resuspended in residual 100 µl of medium. The suspension was streaked on MS agar plates, supplemented with 10 mM $MgCl_2$ and varying concentrations of $CaCl_2$ (10, 25, 50 mM). In contrary to many *Streptomyces* spp., conjugational transfer to *Amycolatopsis* spp. was shown to be crucially dependent on the $CaCl_2$ concentration.[32] After incubation for 16-20 h, the plates were overlaid with 1 mL of water containing 0.5 mg of nalidixic acid (NA) and 1.25 mg of apramycin, and incubation at 30 °C was continued for 5-7 days. Ex-conjugants were streaked on MS (with 50 µg/ml Apr and 25 µg/ml NA). For verification of knock out mutants, a PCR using primers pairs P1P2 and P1P3 was conducted. Therefore, ex-conjugants were inoculated in ISP2 broth containing 50 µg/mL Apr and grown for 3-5 days. After incubation the cultures were centrifuged, and the pellet was frozen in liquid nitrogen and disrupted by grinding. Genomic DNA was extracted using DNeasy Plant Mini Kit (Qiagen) according to manufacturer's instructions. PCR conditions were as follows: 98 °C/5 min, 35 cycles (98 °C/30 sec, 61 °C/45 sec, 72 °C/2 min), 72 °C/10 min. Reactions were performed in GC buffer supplemented with 5% DMSO. The elongation time of 2 min allowed the amplification of the wild type sequence (~2.5 kb) but was too short to observe an amplicon in the single crossover mutants using P1P2. PCR amplification using P1P3, with P3 targeting the suicide vector backbone, results in product formation (~2.5 kb) in the mutant but no product is expected in the wild type. PCR products were then confirmed by Sanger sequencing (Eurofins Genomics, Germany) using primers P1P2 or P1P3, respectively. Confirmed mutants were stored as 50% glycerol stocks prior to further use.

Supplementary Table 14. Plasmids used in this study.

Strain	Description	Reference
pKJ55	*E. coli* – *Streptomyces* shuttle vector, AprR, *oriT* for conjugational transfer	[58]
pKJ55-*2543*-ko	internal fragment of *ctg1_2543* in pKJ55 (*XbaI/SphI*) introduced via Gibson Assembly	this study
pKJ55-*2608*-ko	internal fragment of *ctg1_2608* in pKJ55 (*XbaI/SphI*) introduced via Gibson Assembly	this study
pKJ55-*2755*-ko	internal fragment of *ctg1_2755* in pKJ55 (*XbaI/SphI*) introduced via Gibson Assembly	this study
pKJ55-*191*-ko	internal fragment of *ctg2_191* in pKJ55 (*XbaI/SphI*) introduced via Gibson Assembly	this study
pKJ55-*814*-ko	internal fragment of *ctg2_814* in pKJ55 (*XbaI/SphI*) introduced via Gibson Assembly	this study
pKJ55-*817*-ko	internal fragment of *ctg2_817* in pKJ55 (*XbaI/SphI*) introduced via Gibson Assembly	this study
pKJ55-*869*-ko	internal fragment of *ctg2_869* in pKJ55 (*XbaI/SphI*) introduced via Gibson Assembly	this study
pKJ55-*971*-ko	internal fragment of *ctg2_971* in pKJ55 (*XbaI/SphI*) introduced via Gibson Assembly	this study
pKJ55-*1663*-ko	internal fragment of *ctg2_1663* in pKJ55 (*XbaI/SphI*) introduced via Gibson Assembly	this study
pKJ55-*245*-ko	internal fragment of *ctg3_245* in pKJ55 (*XbaI/SphI*) introduced via Gibson Assembly	this study
pKJ55-*276*-ko	internal fragment of *ctg3_276* in pKJ55 (*XbaI/SphI*) introduced via Gibson Assembly	this study
pKJ55-*552*-ko	internal fragment of *ctg3_552* in pKJ55 (*XbaI/SphI*) introduced via Gibson Assembly	this study
pKJ55-*339*-ko	internal fragment of *ctg4_339* in pKJ55 (*XbaI/SphI*) introduced via Gibson Assembly	this study
pKJ55-*186*-ko	internal fragment of *ctg5_186* in pKJ55 (*XbaI/SphI*) introduced via Gibson Assembly	this study

AprR – apramycin resistance

Supplementary Table 15. Primers used in this study.

Name	Sequence (5’ – 3’)[a]	Purpose
GA_*ctg2_817*_fwd	tccgtcgacctgcaggcatgATCAACTCCTACGGGCCG	forward primer for cloning of pKJ55-*817*-ko
GA_*ctg2_817*_rev	cgcgcgcggccgcggatcctTGACCTGACCGAGGAAGTC	reverse primer for cloning of pKJ55-*817*-ko
GA_*ctg2_971*_fwd	tccgtcgacctgcaggcatgCCCGAAAGACCGAGAAGAC	forward primer for cloning of pKJ55-*971*-ko
GA_*ctg2_971*_rev	cgcgcgcggccgcggatcctCTCAGGGAAAGCTCGTCC	reverse primer for cloning of pKJ55-*971*-ko
GA_*ctg2_1663*_fwd	tccgtcgacctgcaggcatgCACTCCACGTTCCACGAAC	forward primer for cloning of pKJ55-*1663*-ko
GA_*ctg2_1663*_rev	cgcgcgcggccgcggatcctACCGTGTTGAGGTACATG	reverse primer for cloning of pKJ55-*1663*-ko
GA_*ctg3_276*_fwd	tccgtcgacctgcaggcatgGATCACCGGTCCCCTCGATC	forward primer for cloning of pKJ55-*276*-ko
GA_*ctg3_276*_rev	cgcgcgcggccgcggatcctTCGAAGTTGATCGCGGAGAAC	reverse primer for cloning of pKJ55-*276*-ko
GA_*ctg5_186*_fwd	tccgtcgacctgcaggcatgTGAGACCTATGGGAGCGTC	forward primer for cloning of pKJ55-*186*-ko
GA_*ctg5_186*_rev	cgcgcgcggccgcggatcctGATCTGGGACGATAGCAG	reverse primer for cloning of pKJ55-*186*-ko
Seq_pKJ55_IF_fwd	TCTACACGAACCCTTTGGCAA	forward primer for sequencing pKJ55-*gene*-ko vectors
Seq_pKJ55_IF_rev	TTCCGGCTCGTATGTTGTGT	reverse primer for sequencing pKJ55-*gene*-ko vectors
cPCR_*ctg3_276*_fwd (P1)	GTCATCTCACCCTCGTCGTC	forward primer for colony PCR of *Δ276* KO mutant in PS_44_ISF1
cPCR_*ctg3_276*_rev (P2)	GTGAACAGCTCCTGGAACGA	reverse primer for colony PCR of *Δ276* KO mutant in PS_44_ISF1
cPCR_*ctg5_186*_fwd (P1)	CTCGTTCCACACTCGATGGA	forward primer for colony PCR of *Δ186* KO mutant in PS_44_ISF1
cPCR_*ctg5_186*_rev (P2)	AGGATCAGCGTGTTCACGAA	reverse primer for colony PCR of *Δ186* KO mutant in PS_44_ISF1
cPCR_*ctg2_1663*_fwd (P1)	TGTTCCACTCCTACGCGTTC	forward primer for colony PCR of *Δ1663* KO mutant in PS_44_ISF1
cPCR_*ctg2_1663*_rev (P2)	TGAAGAAGGTGTCGACCAGC	reverse primer for colony PCR of *Δ1633* KO mutant in PS_44_ISF1
cPCR_*ctg2_971*_fwd (P1)	GGCTTCGTTCGAATACCGGA	forward primer for colony PCR of *Δ971* KO mutant in PS_44_ISF1
cPCR_*ctg2_971*_rev (P2)	CTCCTTGAACCAGTAGCCGG	reverse primer for colony PCR of *Δ971* KO mutant in PS_44_ISF1
cPCR_*ctg2_817*_fwd (P1)	TCCGGCCTACGTCATCTACA	forward primer for colony PCR of *Δ817* KO mutant in PS_44_ISF1
cPCR_*ctg2_817*_rev (P2)	GCTCGGTGCGGTATTCGAT	reverse primer for colony PCR of *Δ817* KO mutant in PS_44_ISF1
P3	TGTGTGGAATTGTGAGCGGA	reverse primer for colony PCR of KO mutant in PS_44_ISF1

[a] Gene-specific primer binding sequence in upper case letters, overhangs for Gibson assembly in lower case letters.

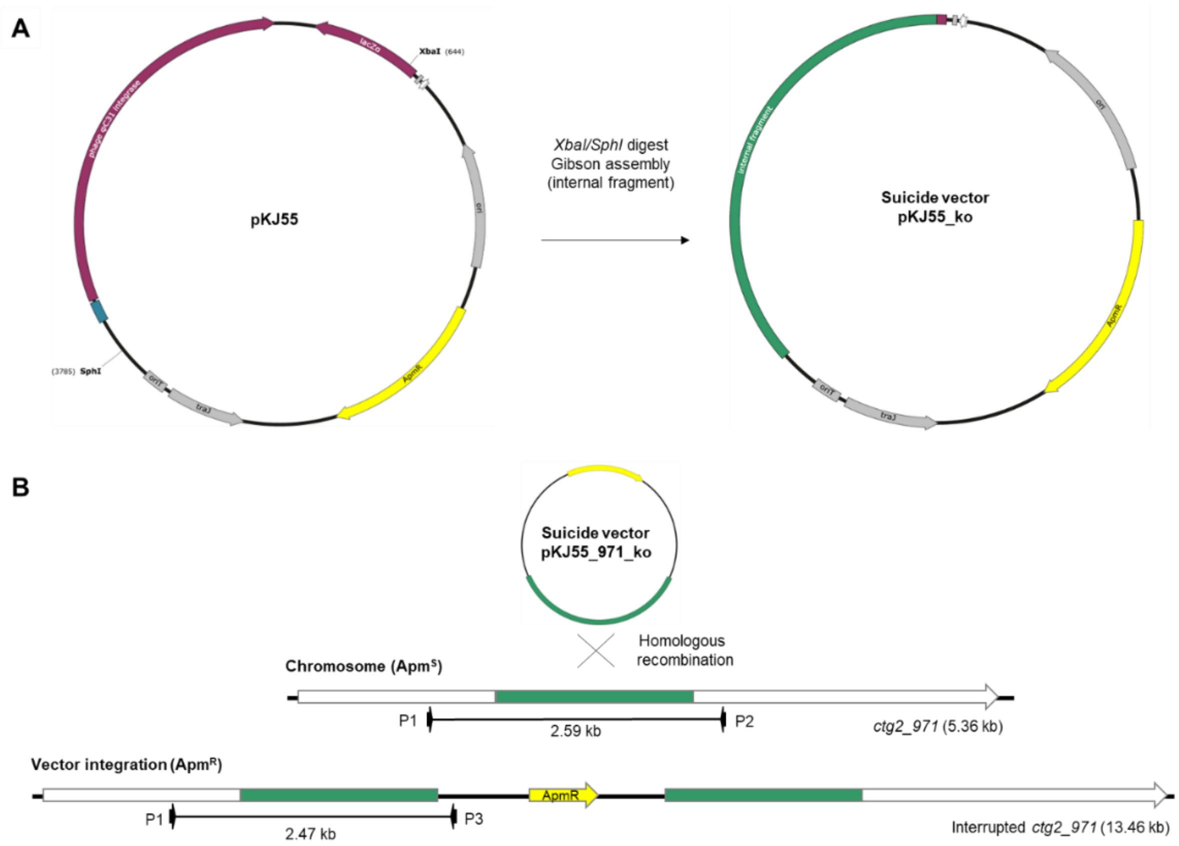

Supplementary Figure 79. Strategy for the generation of knockout mutants of *Amycolatopsis* sp. PS_44_ISF1. (A) General illustration of the construction of suicide vectors deriving from pKJ55. After Gibson Assembly, the genes encoding for page phiC13 integrase including, the attP site and lacZ are replaced by the internal fragment of the target genes. (B) Strategy for the generation of single crossover knockout mutants of relevant NRPS and PKS gene candidates using pKJ55-derived suicide vectors, illustrated for *ctg2_971 (dmgP3)* from BGC region 2.7. For the confirmation of single crossover mutants, primers P1, P2 and P3 were designed.

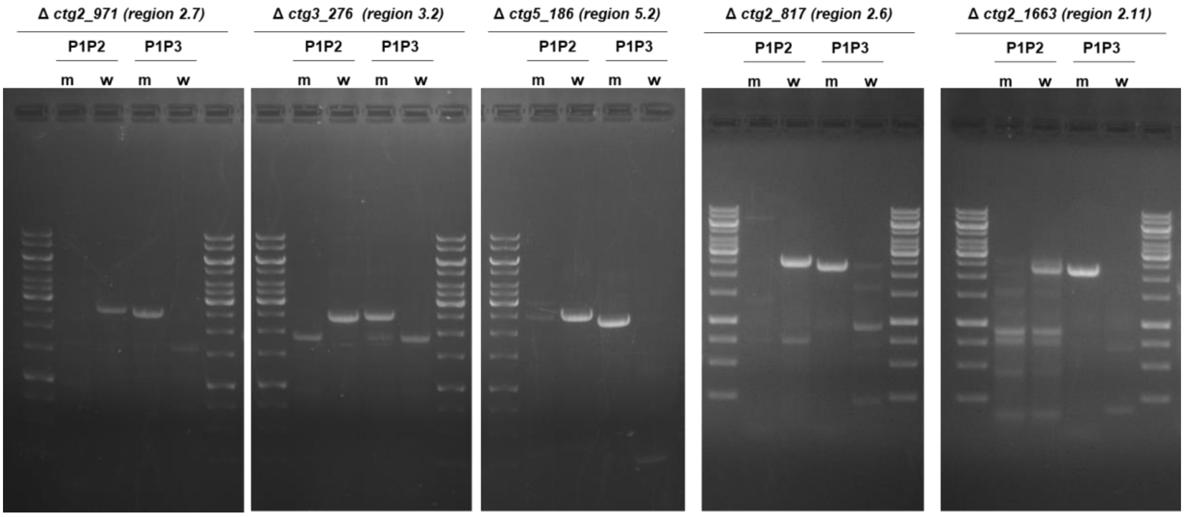

Supplementary Figure 80. Verification of *Amycolatopsis* sp. PS_44_ISF1 knock out mutants. PCR reactions (20 µl) were applied into a 1% agarose gel and electrophoresis was conducted for 45 min at 110V. Primer pairs P1P2 and P1P3 were designed to distinguish between wildtype colonies and insertion mutants. The elongation time of 2 min allowed the amplification of the wildtype sequence (~2.5 kbp) but was too short to observe an amplicon in the single crossover mutants using P1P2. P1P3 with P3 targeting suicide vector backbone result in a product (~2.5 kb) in the mutant but no product in the wildtype. M – Generuler^{TM} 1kb DNA ladder (Thermo Fisher Scientific Inc., USA), templates: m – purified mutant gDNA, w – purified wildtype gDNA.

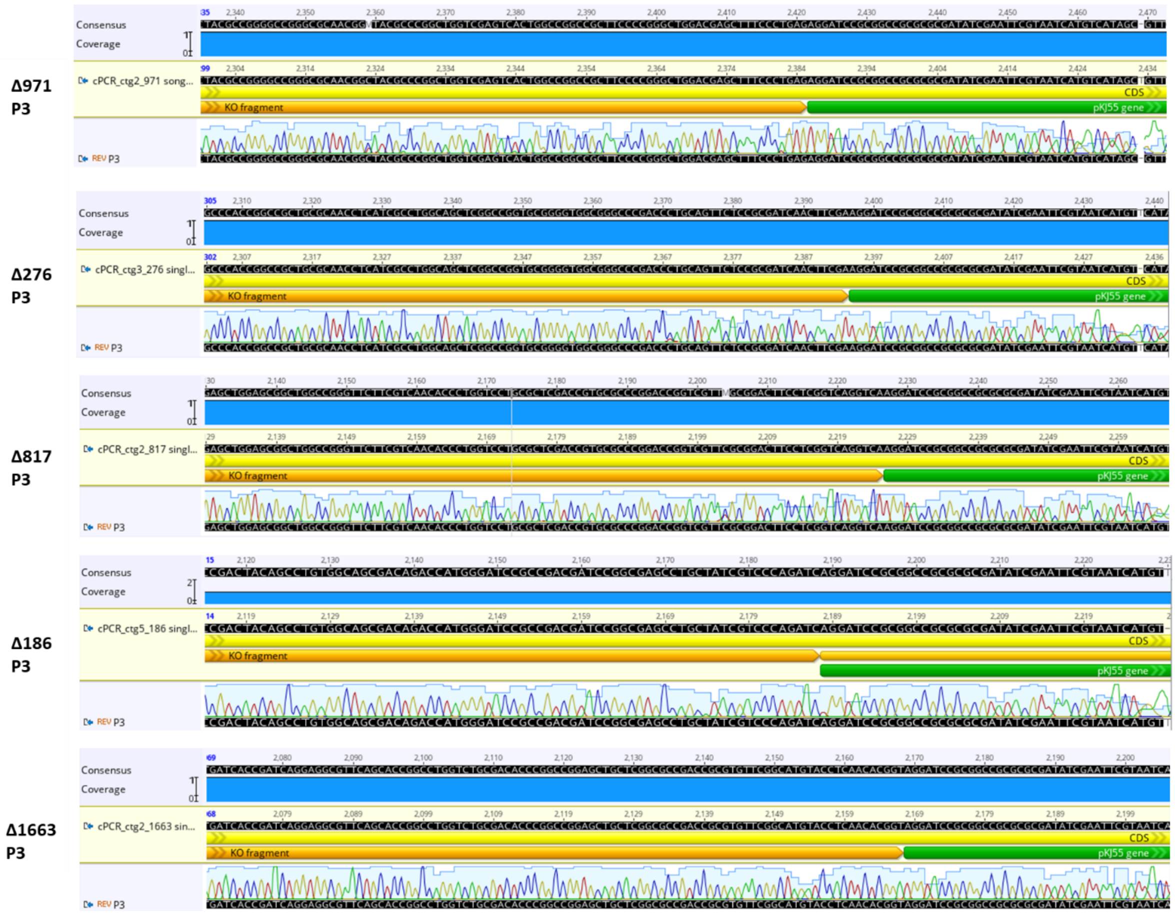

Supplementary Figure 81. Sequencing of *Amycolatopsis* sp. PS_44_ISF1 knock out mutants. After colony PCR (Supplementary Figure 80), PCR products were sequenced using primers P1 and P3. Visualized is the border region at the insertion site of the suicide vector (green area, pKJ55), amplified by primer P3.

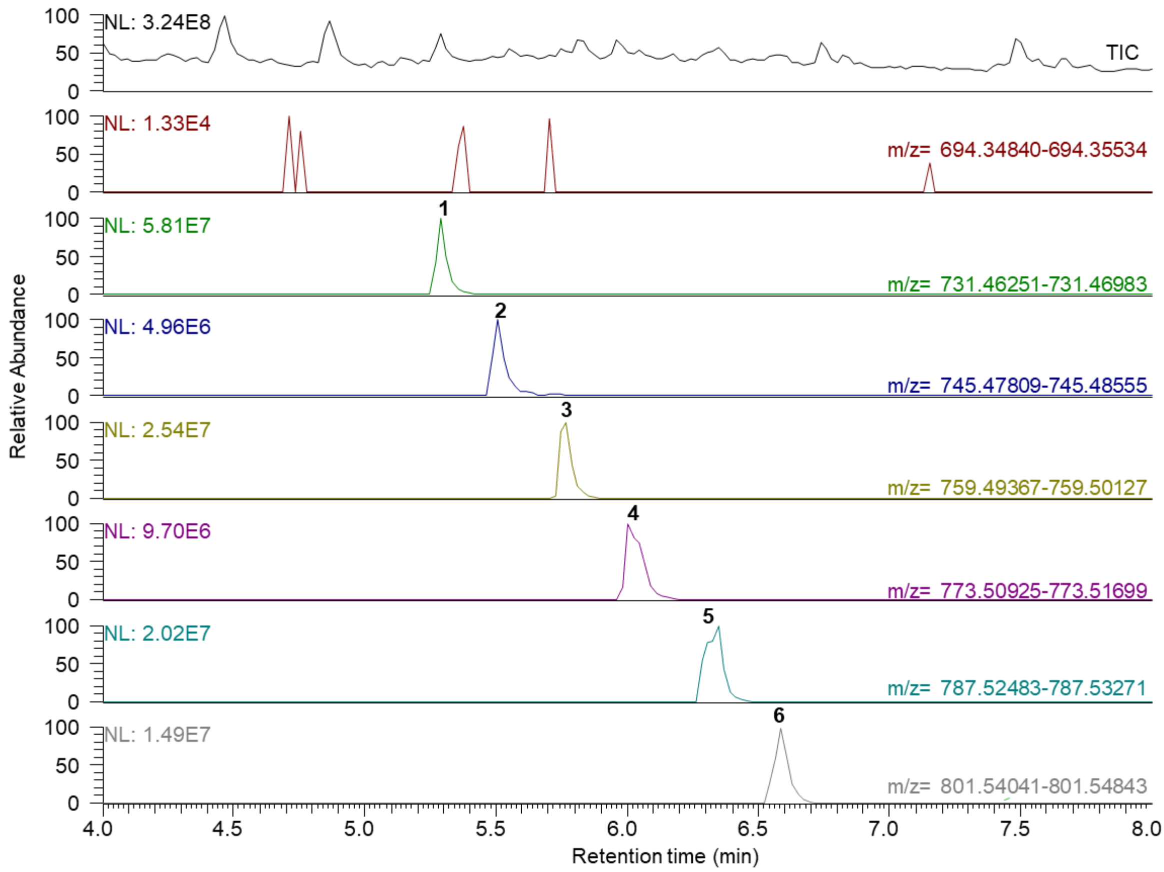

Supplementary Figure 82. Screening of knock out mutant Δ276 (*dmgP5*) for pachycephalamide and demiguisin production (n=3).

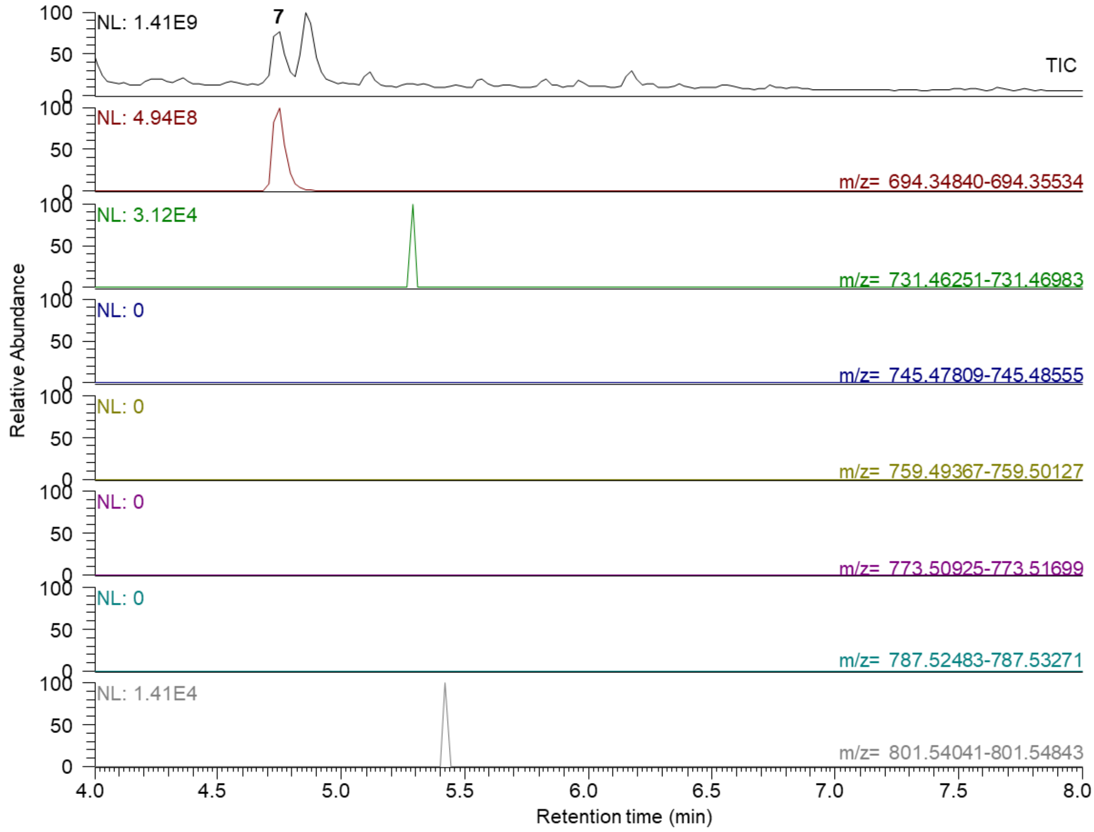

Supplementary Figure 83. Screening of knock out mutant Δ971 (*pchP3*) for pachycephalamide and demiguisin production (n=3).

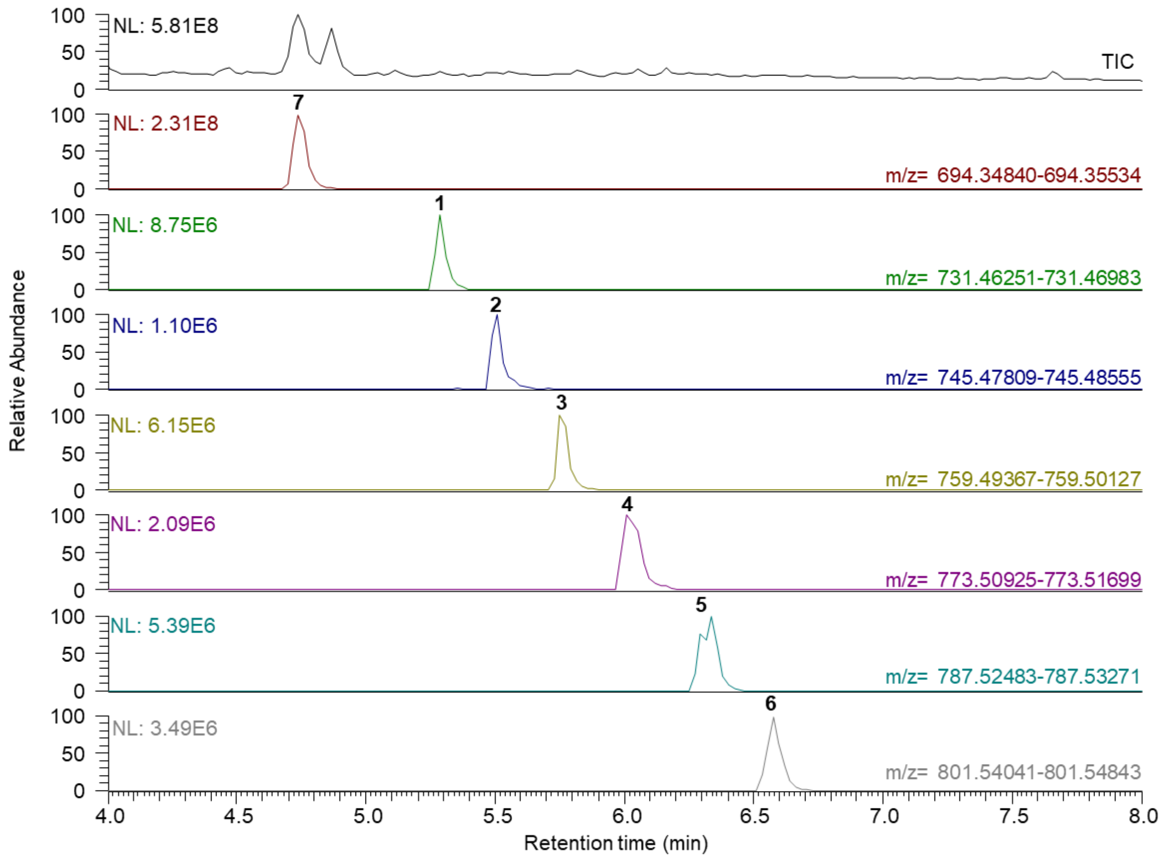

Supplementary Figure 84. Screening of knock out mutant Δ186 for pachycephalamide and demiguisin production (n=3).

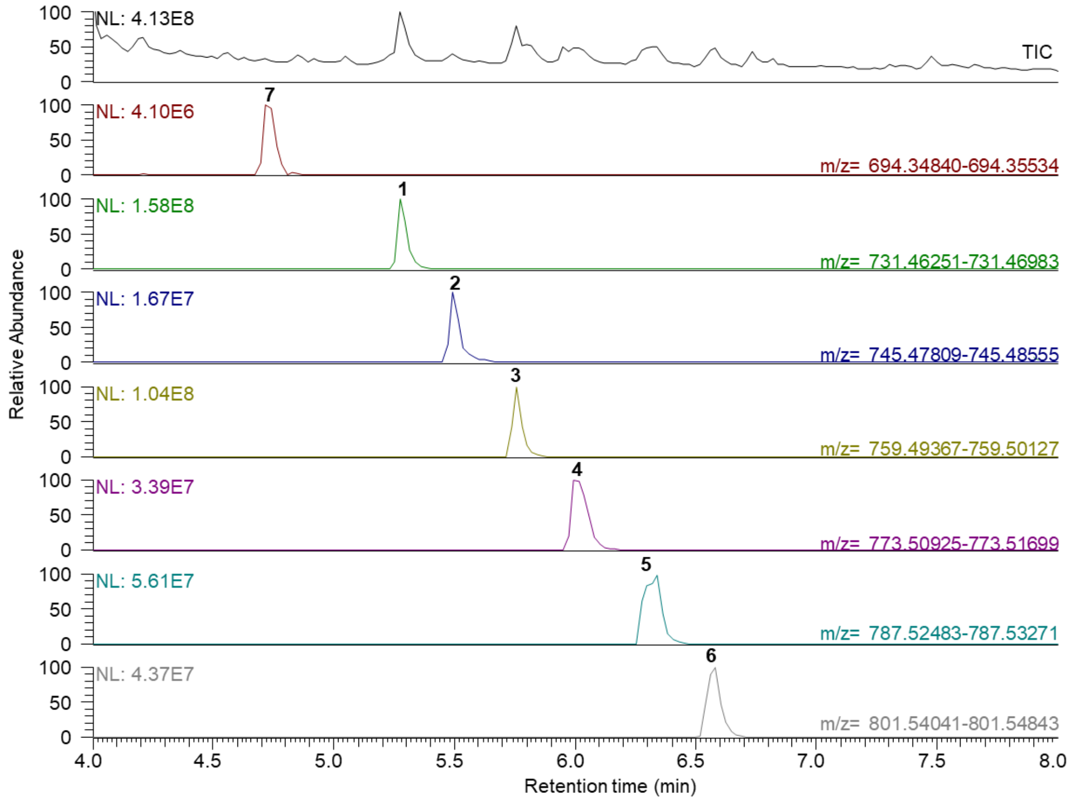

Supplementary Figure 85. Screening of knock out mutant Δ817 for pachycephalamide and demiguisin production (n=3).

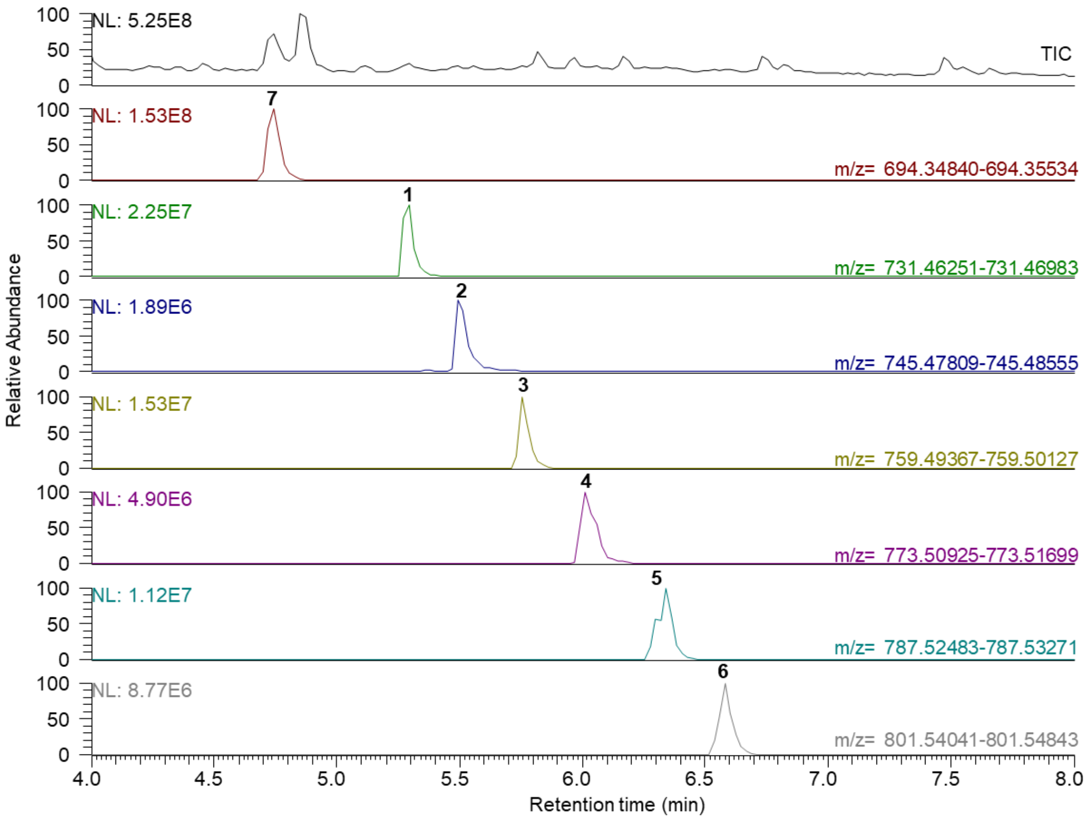

Supplementary Figure 86. Screening of knock out mutant Δ1663 for pachycephalamide and demiguisin production (n=3).

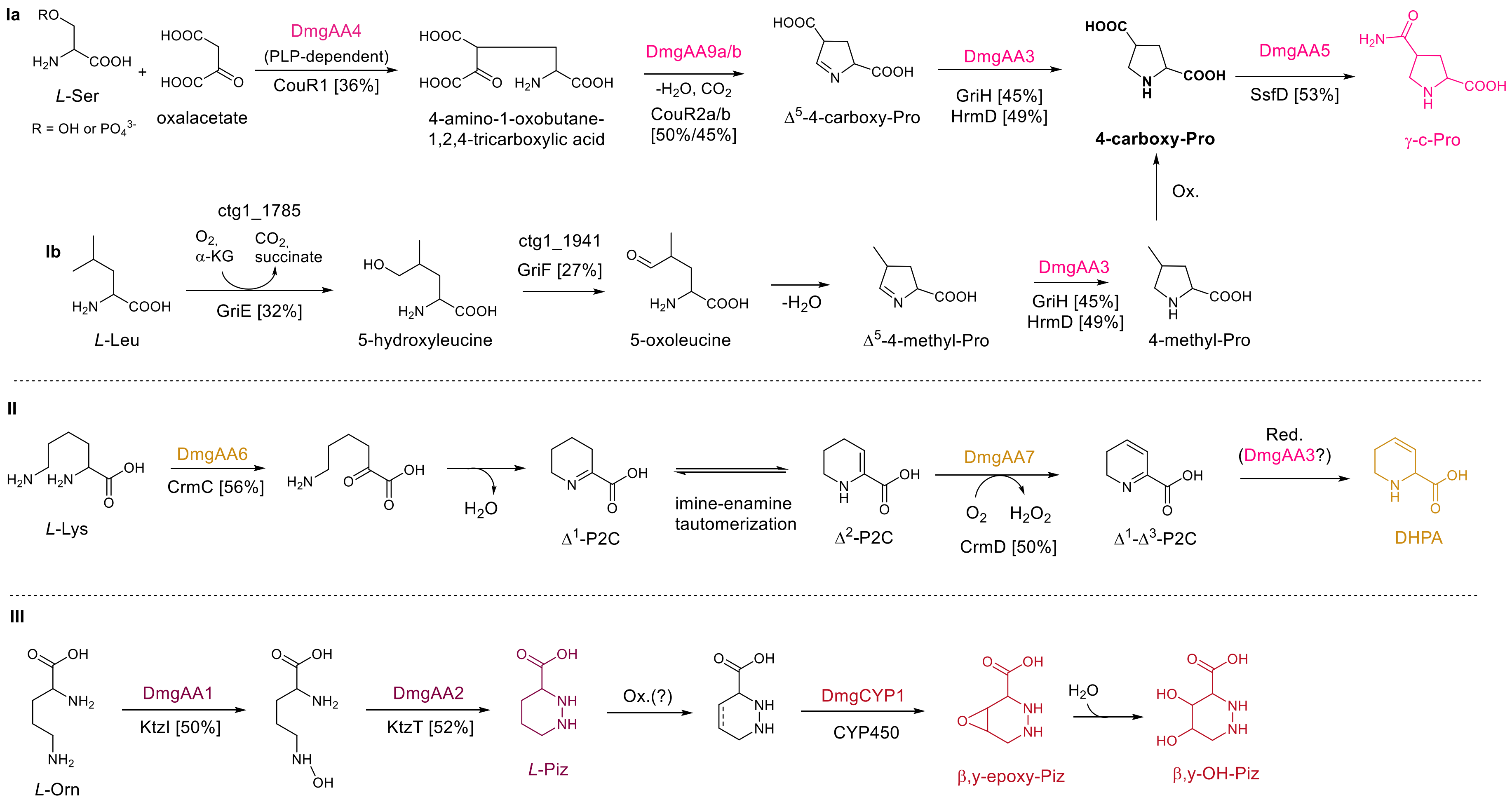

Supplementary Figure 87. Putative biosynthesis of amino acid building blocks necessary for the biosynthesis of demiguisin (*dmg)*.

Droplet-collapsing test of lipopetides.

Pure compounds were dissolved in water (final concentration 10 mM). Then, 20 µl of the solutions were placed on a hydrophobic surface (parafilm 'M'). Tensioactive properties were determined by the observation of collapsed droplets whereas the control (water, no additive) did not collapse. [33,34] Tween-20 (10 mM) was used as tensioactive positive control. For better visualization, 0.0025% crystal violet was added to the droplet, having no effect on the droplet shape.

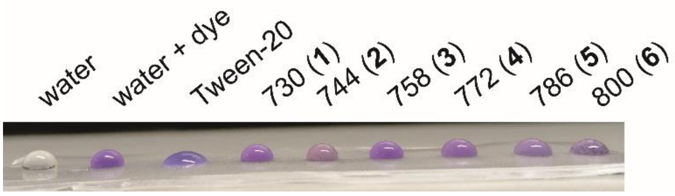

Supplementary Figure 88. Droplet-collapsing assay testing the tensioactive properties of lipopeptides (n=3).

Disc-diffusion antimicrobial activity assays against ecologically relevant indicator strains.

SPE fractions and pure compounds (10 mg/mL in DMSO) were tested for their antimicrobial activity against ecologically relevant indicator strains, including feather-degrading bacteria *B. licheniformis* DSM13, *P. monteilii* DSM1388 and *K. rhizophila* DSM11926, and non-degrading feather isolates *B. thuringiensis* DSM104061 and *S. epidermidis* DSM103867, as well as the skin pathogenic yeast *C. albicans* SC5314. For this a disc diffusion assay was applied using 5 µl compound solution (50 µg/mL). Antimicrobial activity was determined by measuring the inhibition zone surrounding the disc in mm. Ciprofloxacin and Amphotericin B served as control.

Supplementary Table 16. Disc-diffusion assays of lipopeptides. Bl: *Bacillus licheniformis* DSM13, Kr: *Kocuria rhizophila* DSM11926, Bt: *B. thuringiensis* DSM104061, Pm: *Pseudomonas monteilii* DSM1388, Se: *Staphylococcus epidermidis* DSM103867, Ca2: *Candida albicans* SC5314, n.t.: not tested. All compounds were also tested in a 1:1 (v/v) ratio (mixture of all compounds in equal amounts, 10 mg/ml) and in the calculated ratio observed in MeOH extracts (ratio (v/v) 1:2:3:4:5:6 = 8:1:5:1:3:1) (n=1).

			Indicator strains					
cmp.	conc.	solvent	Bl	Kr	Bt	Pm	Se	Ca2
**1**	10 mg/mL	DMSO	0	0	0	0	0	0
**2**	10 mg/mL	DMSO	0	0	0	0	0	0
**3**	10 mg/mL	DMSO	0	0	0	0	0	0
**4**	10 mg/mL	DMSO	0	0	0	0	0	0
**5**	10 mg/mL	DMSO	0	7	0	0	0	0
**6**	10 mg/mL	DMSO	0	10	0	0	0	0
**1-6**	1:1 ratio	DMSO	0	14	0	0	0	0
**1-6**	Ratio in enriched MeOH extracts (8:1:5:1:3:1)	DMSO	0	11	0	0	0	0
		DMSO	0	0	0	0	0	0
Cip	5 µg/mL	HCl	35	22	28	33	40	n.t.
Amph B	10 µg/mL	DMSO/H_2O	n.t.	n.t.	n.t.	n.t.	n.t.	20

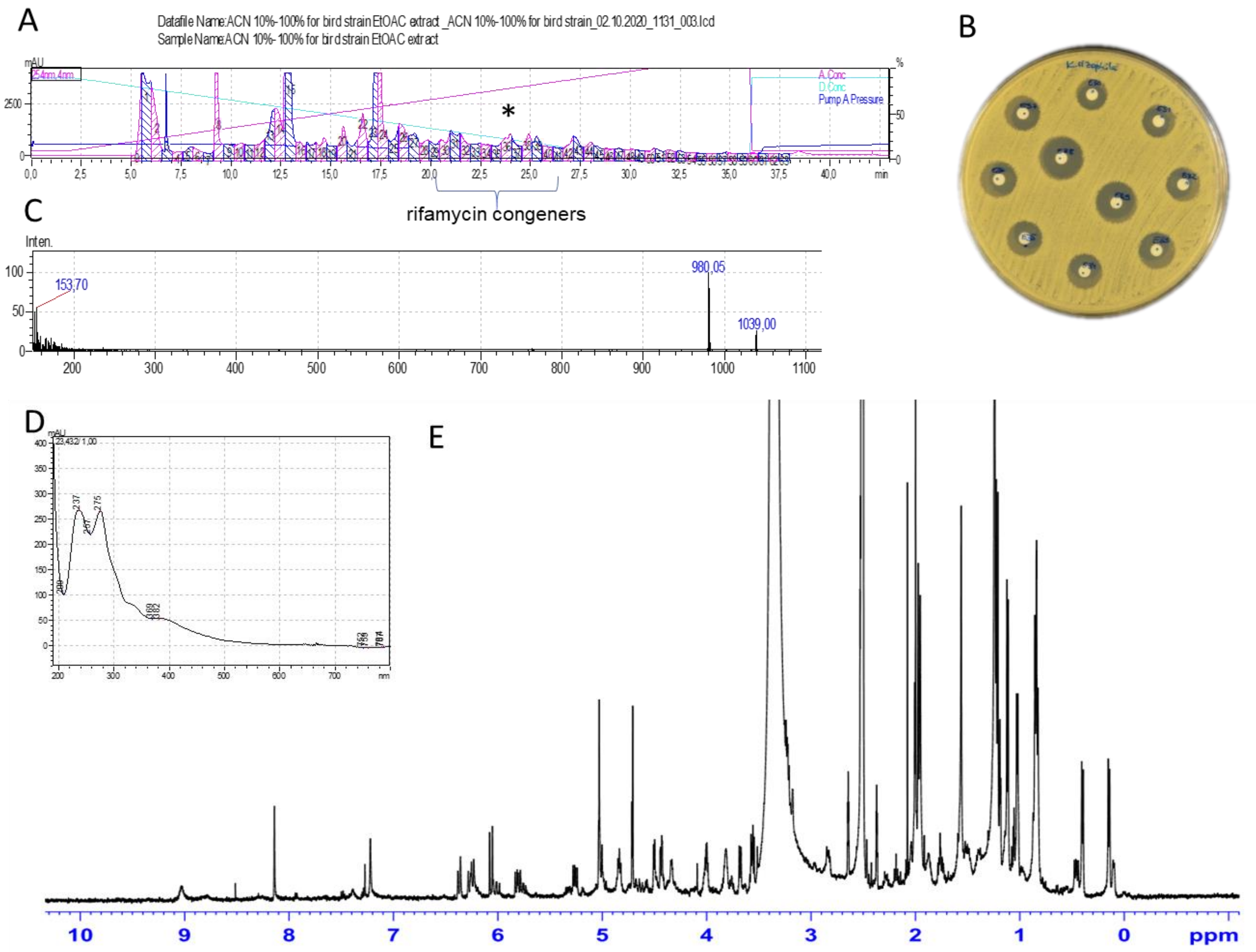

Supplementary Figure 89. Co-secreted ansamycin-based rifamycin congeners. A) HPLC chromatogram showing fractionation of EtOAc-based culture extracts. B) Representative disc-diffusion assay of HPLC fractions 31-39 against *K. rhizophila*. C) Low-resolution analysis of enriched rifamycin fraction and D) UV-Vis spectrum of enriched fraction. E) ^{1}H NMR of fraction-enriched with one dominant rifamycin congener.

Disc-diffusion antimicrobial activity assays against clinically relevant indicator strains.

Pure compounds (1 mg/mL in DMSO or 50% MeOH) were tested for their antimicrobial activity against a variety of bacterial and fungal indicator strains: *B. subtilis* 6633 (Bs), *S. aureus* SG511 (Sa1), *E. coli* SG458 (Ec), *P. aeruginosa* K799/61 (Pa), *S. aureus* 134/94 (MRSA) (Sa2), *E. faecalis* 1528 (VRSA) (Ef), *Mycobacterium vaccae* 10670 (Mv), *Sporobomyces salmonicolor* 549 (Ss), *C. albicans* C.A. (Ca), and *Penicillium notatum* JP36 (Pn). On each disc, 50 µl compound solution (50 µg) were applied. Antimicrobial activity was determined by measuring the inhibition zone surrounding the disc in mm (n=3).

Supplementary Table 17. Antimicrobial activity based on disc-diffusion assays of isolated compounds from *Amycolatopsis* sp. PS_44_ISF1. Bs: B*. subtilis* 663, Sa1: *S. aureus* SG511, Ec: *E. coli* SG458, Pa: *P. aeruginosa* K799/61, Sa2: *S. aureus* 134/94 (MRSA), Ef: *E. faecalis* 1528 (VRSA), Mv: *M. vaccae* 10670, Ss: *S. salmonicolor* 549, Ca: *C. albicans* C.A., Pn: *P. notatum* JP36. P: colonies in the inhibition zone, p: single colonies in the inhibition zone. A: no to low inhibition, n.t.: not tested. For tests with DMSO an inhibitory activity of the solvent was observed. Therefore, values exceeding the inhibition of the solvent are highlighted in green.

			Indicator strains									
cmp.	conc.	solvent	Bs	Sa1	Ec	Pa	Sa2	Ef	Mv	Ss	Ca	Pn
**1**	1 mg/mL	DMSO	0	0	0	18P	0	0/A	0	15p	0/A	11p
**2**	1 mg/mL	DMSO	0	0	15P	18P	0	0/A	0/A	14p	0	13p
**3**	1 mg/mL	DMSO	0	0	15P	11/18P	0	0/A	0/A	22p	0	13p
**4**	1 mg/mL	DMSO	0	0	13P	18P	0	0/A	0	25p	0	12p
**5**	1 mg/mL	DMSO	0	0	15P	17P	0	13p	0/A	19p	0	14p
**6**	1 mg/mL	DMSO	0	0	13P	18P	0	13p	13p	23p	0	13p
**7**	1 mg/mL	DMSO	0	0	0	0	0	0	0	0	0	0
**7+8 (9:1)**	1 mg/mL	DMSO	0	0	15P	11/18P	0	0/A	0	15p	15P	12p
control		DMSO	12P	11P	12P	12P	0	12p	11P	13P	0	12P
Cip	5 µg/mL	H_2O/HCl	28	19	23/31p	26/33p	0	16F	21p	n.t.	n.t.	n.t.
Amph B	10 µg/mL	DMSO/H_2O	n.t.	n.t.	n.t.	n.t.	n.t.	n.t.	n.t.	17p	20	18p

Supplementary Table 18. Antimicrobial activity based on disc-diffusion assays of compound mixtures. Bs: *B. subtilis* 663, Sa1: *S. aureus* SG511, Ec: *E. coli* SG458, Ca: *C. albicans* C.A., P: colonies in the inhibition zone, n.t.: not tested. To analyze if the compounds act synergistically in mixture, all compounds were mixed in a 1:1 ratio (n=1).

					Indicator strains			
Entry	Mixture of comp	conc. of each cmp.	Total conc.	solvent	Ca	Bs	Sa1	Ec
1	**7+8 (9:1)**		1 mg/mL	50% MeOH	42P	0	0	0
2	**7+8 (9:1)**		0.5 mg/mL	50% MeOH	39P	0	0	0
3	**7+8 (9:1)**		0.14 mg/mL	50% MeOH	30P	0	0	0
4	**1-8**	0.14 mg/ml	1 mg/mL	50% MeOH	25P	0	0	0
5	**1-6**	0.17 mg/mL	1 mg/mL	50% MeOH	0	0	0	0
6	**1+7+8**	0.5 mg/mL	1 mg/mL	50% MeOH	30P	0	0	0
7	**2+7+8**	0.5 mg/mL	1 mg/mL	50% MeOH	20P	0	0	0
8	**3+7+8**	0.5 mg/mL	1 mg/mL	50% MeOH	38P	0	0	0
9	**4+7+8**	0.5 mg/mL	1 mg/mL	50% MeOH	35P	0	0	0
10	**5+7+8**	0.5 mg/mL	1 mg/mL	50% MeOH	25P	0	0	0
11	**6+7+8**	0.5 mg/mL	1 mg/ml	50% MeOH	40P	0	0	0
12	control			50% MeOH	0	0	0	0
13	Cip		5 µg/mL	HCl	n.t.	26/33p	0	22p
14	Amph B		10 µg/mL	DMSO/H_2O	21	n.t.	n.t.	n.t.

Protease inhibition assays.

Compounds were dissolved in DMSO (final concentration 20 mM). Fluorimetric assays were then performed at a concentration of 200 µM. The inhibitory effect of the compounds was determined for the following enzymes: rhodesain, cruzain, cathepsin L, cathepsin B, SARS-CoV2 M^{pro}, SARS-CoV PL^{pro}, DENV2 NS2B-NS3, ZIKV NS2B-NS3, urokinase (uPA), sortase A (SrtA). [35,36] The increase of the fluorescence was recorded over a period of 10 minutes in intervals of 30 seconds (exception: SrtA 30 minutes). Inhibition was calculated from the slope of fluorescence increase in the presence of the compound in relation to the slope in the presence of DMSO. The Inhibition rate is shown in percentage as mean value ± standard deviation and was determined in triplicates (n=3). Assay conditions were: 45 µL buffer, 1.25 µL enzyme in buffer, 2.5 µL compound in DMSO, 1.25 µL substrate in DMSO (total volume 50 µL).

Supplementary Table 19. Protease inhibition rate of metabolites at 200 µM (n=3, standard deviation).

	**1**	**2**	**3**	**4**	**5**	**6**
**Rhodesain**	55.29 ± 2.15	47.71 ± 2.73	58.33 ± 1.36	72.76 ± 1.07	48.22 ± 1.63	53.72 ± 0.31
**Cruzain**	64.85 ± 1.50	49.62 ± 3.44	63.82 ± 1.43	73.97 ± 1.54	43.80 ± 2.31	60.52 ± 3.73
**Cathepsin L**	61.88 ± 2.85	50.42 ± 1.21	62.51 ± 4.09	78.67 ± 2.19	47.31 ± 2.53	58.30 ± 6.91
**Cathepsin B**	55.19 ± 2.16	54.94 ± 2.96	56.16 ± 6.18	73.50 ± 4.05	47.52 ± 9.52	57.57 ± 11.52
**M^{pro} (SARS-CoV2)**	12.82 ± 8.66	57.34 ± 9.94	9.25 ± 7.28	24.32 ± 2.62	14.75 ± 7.06	6.92 ± 5.97
**PL^{pro} (SARS-CoV)**	1.01 ± 1.74	14.94 ± 1.31	0 ± 0	2.05 ± 2.29	0 ± 0	0 ± 0
**DENV2**	8.57 ± 3.83	29.25 ± 7.00	9.19 ± 3.06	33.81 ± 3.08	19.02 ± 6.12	18.47 ± 1.66
**ZIKV**	7.99 ± 3.53	29.95 ± 3.36	8.07 ± 8. 26	35.04 ± 1.21	22.71 ± 1.47	22.62 ± 1.19
**uPA**	8.32 ± 4.90	0 ± 0	0 ± 0	0.56 ± 0.90	0 ± 0	0 ± 0
**SrtA**	12.86 ± 2.35	61.07 ± 4.93	14.00 ± 4.62	28.78 ± 4.59	17.94 ± 1.28	20.39 ± 3.94

Supplementary References

[1] https://www.frontiersin.org/articles/10.3389/fmicb.2020.01735/full

[2] Miller CS, Handley KM, Wrighton KC, Frischkorn KR, Thomas BC, Banfield JF. Short-Read Assembly of Full-Length 16S Amplicons Reveals Bacterial Diversity in Subsurface Sediments. *PLOS ONE* **8**, e56018 (2013).

[3] Meier-Kolthoff JP, Göker M. TYGS is an automated high-throughput platform for state-of-the-art genome-based taxonomy. *Nature Com*. 10, 2182 (2019).

[4] Ondov BD, et al. Mash: fast genome and metagenome distance estimation using MinHash. Genome Biol 17, 132 (2016).

[5] Lagesen K, Hallin P, Rødland EA, Stærfeldt H-H, Rognes T, Ussery DW. RNAmmer: consistent and rapid annotation of ribosomal RNA genes. Nucleic Acids Research 35, 3100-3108 (2007).

[6] Camacho C, et al. BLAST+: architecture and applications. BMC Bioinformatics 10, 421 (2009).

[7] Meier-Kolthoff JP, Auch AF, Klenk HP, Göker M. Genome sequence-based species delimitation with confidence intervals and improved distance functions. *BMC Bioinformatics* **14**, 60 (2013).

[8] Meier-Kolthoff JP, Carbasse JS, Peinado-Olarte RL, Göker M. TYGS and LPSN: a database tandem for fast and reliable genome-based classification and nomenclature of prokaryotes. *Nucleic Acids Res* 50, D801-d807 (2022).

[9] Lefort V, Desper R, Gascuel O. FastME 2.0: A Comprehensive, Accurate, and Fast Distance-Based Phylogeny Inference Program. Mol Biol Evol 32, 2798-2800 (2015).

[10] Kreft Ł, Botzki A, Coppens F, Vandepoele K, Van Bel M. PhyD3: a phylogenetic tree viewer with extended phyloXML support for functional genomics data visualization. Bioinformatics 33, 2946-2947 (2017).

[11] Meier-Kolthoff JP, Hahnke RL, Petersen J, Scheuner C, Michael V, Fiebig A, et al. Complete genome sequence of DSM 30083T, the type strain (U5/41T) of Escherichia coli, and a proposal for delineating subspecies in microbial taxonomy. Stand Genomic Sci., 9, 2 (2014).

[12] Meier-Kolthoff JP, Göker M. TYGS is an automated high-throughput platform for state-of-the-art genome-based taxonomy. Nature Communications 10, 2182 (2019).

[13] Adamek M, et al. Comparative genomics reveals phylogenetic distribution patterns of secondary metabolites in Amycolatopsis species. BMC Genomics 19, 426 (2018).

[14] Gopinath SCB, Anbu P, Hilda A. Extracellular enzymatic activity profiles in fungi isolated from oil-rich environments. *Mycoscience* **46**, 119-126 (2005).

[15] Singh R, Gupta N, Goswami VK, Gupta R. A simple activity staining protocol for lipases and esterases. *Appl Microbiol Biotechnol* **70**, 679-682 (2006).

[16] Jacob J. Hydrocarbon and multibranched ester waxes from the uropygial gland secretion of grebes (Podicipediformes). *J Lipid Res* **19**, 148-153 (1978).

[17] Singh R, Gupta N, Goswami VK, Gupta R. A simple activity staining protocol for lipases and esterases. *Appl Microbiol Biotechnol* **70**, 679-682 (2006).

[18] Han M, Luo W, Gu Q, Yu X. Isolation and characterization of a keratinolytic protease from a feather-degrading bacterium Pseudomonas aeruginosa C11. *African Journal of Microbiology Research* **6**, 2211-2221 (2012).

[19] Dada MT, Wakil SM. Screening and Characterisation of Keratin-Degrading Bacillus sp. from Feather Waste. *Biotechnology Journal International* **23**, 1-12 (2019).

[20] Kieser T, Foundation JI. *Practical Streptomyces Genetics*. John Innes Foundation (2000).

[21] Hoffmann T, Dorrestein PC. Homogeneous matrix deposition on dried agar for MALDI imaging mass spectrometry of microbial cultures. J Am Soc Mass Spectrom 26, 1959-1962 (2015).

[22] Crameri F, Shephard GE, Heron PJ. The misuse of colour in science communication. Nature Communications 11, 5444 (2020).

[23] Watrous JD, *et al.* Microbial metabolic exchange in 3D. *The ISME Journal* **7**, 770-780 (2013)

[24] Oh DC, Poulsen M, Currie CR, Clardy J. Dentigerumycin: a bacterial mediator of an ant-fungus symbiosis. *Nat Chem Biol* **5**, 391-393 (2009).

[25] Klau LJ, *et al.* The Natural Product Domain Seeker version 2 (NaPDoS2) webtool relates ketosynthase phylogeny to biosynthetic function. *Journal of Biological Chemistry* **298**, 102480 (2022).

[26] Larkin MA, *et al.* Clustal W and Clustal X version 2.0. *Bioinformatics* **23**, 2947-2948 (2007).

[27] Price MN, Dehal PS, Arkin AP. FastTree 2 – Approximately Maximum-Likelihood Trees for Large Alignments. *PLOS ONE* **5**, e9490 (2010).

[28] Community TG. The Galaxy platform for accessible, reproducible and collaborative biomedical analyses: 2022 update. *Nucleic Acids Research* **50**, W345-W351 (2022).

[29] Medema MH, *et al.* antiSMASH: rapid identification, annotation and analysis of secondary metabolite biosynthesis gene clusters in bacterial and fungal genome sequences. *Nucleic Acids Research* **39**, W339-W346 (2011).

[30] Klau LJ, *et al.* The Natural Product Domain Seeker version 2 (NaPDoS2) webtool relates ketosynthase phylogeny to biosynthetic function. *Journal of Biological Chemistry* **298**, 102480 (2022)

[31] Caffrey P. Conserved Amino Acid Residues Correlating With Ketoreductase Stereospecificity in Modular Polyketide Synthases. *ChemBioChem* **4**, 654-657 (2003).

[32] Li C, Zhou L, Wang Y, Zhao G, Ding X. Conjugation of ϕBT1-derived integrative plasmid pDZL802 in Amycolatopsis mediterranei U32. *Bioengineered* **8**, 549-554 (2017).

[33] Dose B, Niehs SP, Scherlach K, Flórez LV, Kaltenpoth M, Hertweck C. Unexpected Bacterial Origin of the Antibiotic Icosalide: Two-Tailed Depsipeptide Assembly in Multifarious Burkholderia Symbionts. *ACS Chemical Biology* **13**, 2414-2420 (2018).

[34] Jain DK, Collins-Thompson DL, Lee H, Trevors JT. A drop-collapsing test for screening surfactant-producing microorganisms. *Journal of Microbiological Methods* **13**, 271-279 (1991).

[35] A) Millies B, *et al.* Proline-Based Allosteric Inhibitors of Zika and Dengue Virus NS2B/NS3 Proteases. *Journal of Medicinal Chemistry* **62**, 11359-11382 (2019); b)Klein P, *et al.* Naphthoquinones as Covalent Reversible Inhibitors of Cysteine Proteases—Studies on Inhibition Mechanism and Kinetics. *Molecules* **25**, 2064 (2020); c)

Welker A, *et al.* Structure-Activity Relationships of Benzamides and Isoindolines Designed as SARS-CoV Protease Inhibitors Effective against SARS-CoV-2. *ChemMedChem* **16**, 340-354 (2021).

[36] Barthels F, *et al.* Asymmetric Disulfanylbenzamides as Irreversible and Selective Inhibitors of Staphylococcus aureus Sortase A. *ChemMedChem* **15**, 839-850 (2020).
