# Supplementary material for: Bacteria from the Amycolatopsis genus associated with a toxic bird secrete protective secondary metabolites
Source: Nat Commun. 2024 Oct 2;15:8524. doi: 10.1038/s41467-024-52316-3 (PMC11446937; doi:10.1038/s41467-024-52316-3)
Supplement: Supplementary file 3 — Description of additional supplementary files [file 41467_2024_52316_MOESM3_ESM.pdf]

<u>Description of Additional Supplementary Files</u>

Supplementary Data 1: dDDH values used for phylogenetic analysis and strain identification

Supplementary Data 2: Data of type strains used for strain identification

Supplementary Data 3: Proteins encoded in the *pch* BGC and comparison to NCBI and MiBIG database

Supplementary Data 4: Proteins encoded in the *dmg* BGC (region R3.2) and comparison to NCBI and MiBIG database
