# Supplementary material for: Bacteria from the Amycolatopsis genus associated with a toxic bird secrete protective secondary metabolites
Source: Nat Commun. 2024 Oct 2;15:8524. doi: 10.1038/s41467-024-52316-3 (PMC11446937; doi:10.1038/s41467-024-52316-3)
Supplement: Supplementary file 8 — Reporting Summary [file 41467_2024_52316_MOESM8_ESM.pdf]

nature portfolio

Corresponding author(s): Christine Beemelmanns

Last updated by author(s): Aug 26, 2024

Reporting Summary

Nature Portfolio wishes to improve the reproducibility of the work that we publish. This form provides structure for consistency and transparency in reporting. For further information on Nature Portfolio policies, see our Editorial Policies and the Editorial Policy Checklist.

Statistics

For all statistical analyses, confirm that the following items are present in the figure legend, table legend, main text, or Methods section.

n/a	Confirmed	
☐	☒	The exact sample size (*n*) for each experimental group/condition, given as a discrete number and unit of measurement
☐	☒	A statement on whether measurements were taken from distinct samples or whether the same sample was measured repeatedly
☐	☒	The statistical test(s) used AND whether they are one- or two-sided<br>*Only common tests should be described solely by name; describe more complex techniques in the Methods section.*
☒	☐	A description of all covariates tested
☒	☐	A description of any assumptions or corrections, such as tests of normality and adjustment for multiple comparisons
☐	☒	A full description of the statistical parameters including central tendency (e.g. means) or other basic estimates (e.g. regression coefficient) AND variation (e.g. standard deviation) or associated estimates of uncertainty (e.g. confidence intervals)
☐	☒	For null hypothesis testing, the test statistic (e.g. *F*, *t*, *r*) with confidence intervals, effect sizes, degrees of freedom and *P* value noted<br>*Give P values as exact values whenever suitable.*
☒	☐	For Bayesian analysis, information on the choice of priors and Markov chain Monte Carlo settings
☒	☐	For hierarchical and complex designs, identification of the appropriate level for tests and full reporting of outcomes
☒	☐	Estimates of effect sizes (e.g. Cohen's *d*, Pearson's *r*), indicating how they were calculated

Our web collection on statistics for biologists contains articles on many of the points above.

Software and code

Policy information about availability of computer code

Data collection	MinKNOW software version 4.0.20 (Oxford Nanopore Technologies), flexAnalysis 3.3, flexImaging 3.0 and SCILS Lab 2015b; LTSC Thermo Scientific Xcalibur 4.7; TopSpin; Chromeleon
Data analysis	Sequencing: QIIME2 and DADA2 pipelines, phyloseq and vegan packages; FastQC version 0.11.8 and MultiQC version 1.7; Jellyfish version 2.2.10 and GenomeScope; MinKNOW software version 4.0.20, Guppy version 4.2.2; Nanofilt; MaSuRCA version 3.4.1; POLCA.<br>Mass spectrometry: GNPS platform (http://gnps.ucsd.edu); MS-Convert; Cytoscape 3.8.0 ; MassFrontier 8.0; flexAnalysis 3.3, flexImaging 3.0 and SCILS Lab 2015b; TopSpin 3.2 and Mnova NMR. ClustalW multiple alignment tool; natural product domain seeker database NaPDoS2_v13b; FASTTREE tool; Galaxy platform; Geneious Prime; FigTree v1.4.4; antiSMASH v.7beta (strict version); GIMP; Chemdraw; Word 2016; Adobe Illustrator; RStudio v.1.4.1106 ; pheatmap package v1.0.12; viridis package v0.6.2

For manuscripts utilizing custom algorithms or software that are central to the research but not yet described in published literature, software must be made available to editors and reviewers. We strongly encourage code deposition in a community repository (e.g. GitHub). See the Nature Portfolio guidelines for submitting code & software for further information.

Data

Policy information about availability of data

All manuscripts must include a data availability statement. This statement should provide the following information, where applicable:

- Accession codes, unique identifiers, or web links for publicly available datasets
- A description of any restrictions on data availability
- For clinical datasets or third party data, please ensure that the statement adheres to our policy

Supplementary Information contains all details to experimental and analytical data. Analytical data (1D, 2D NMR) is available on Zenodo [doi: 10.5281/zenodo.13125628] but currently under embargo until the 31-08-2024].
The WGS data used in this study are available in the NCBI database under accession code JANUXO000000000 [https://www.ncbi.nlm.nih.gov/search/all/?term=JANUXO000000000]. The SRA data used in this study are available in the NCBI database under accession code SRR21206835-SRR21206837 [https://www.ncbi.nlm.nih.gov/search/all/?term=SRR21206835]. RNA sequencing data used in this study are available in the NCBI database under accession code SRR21206833 and SRR21206834 and data analysis of this study are provided in the Supplementary Source Data file. The amplicon sequencing data generated in this study have been deposited in the NCBI database under accession code SAMN30849808-SAMN30849828 [will be released upon publication]. We have uploaded the MS-Data to the MAssIVE Server (ID: MassIVE MSV000093302) [https://massive.ucsd.edu/ProteoSAFe/dataset.jsp?task=7c1ba2e849ed42f88592c69f6a599d83]

Research involving human participants, their data, or biological material

Policy information about studies with human participants or human data. See also policy information about sex, gender (identity/presentation), and sexual orientation and race, ethnicity and racism.

Reporting on sex and gender	n/a
Reporting on race, ethnicity, or other socially relevant groupings	n/a
Population characteristics	n/a
Recruitment	n/a
Ethics oversight	n/a

Note that full information on the approval of the study protocol must also be provided in the manuscript.

Field-specific reporting

Please select the one below that is the best fit for your research. If you are not sure, read the appropriate sections before making your selection.

☒ Life sciences ☐ Behavioural & social sciences ☐ Ecological, evolutionary & environmental sciences

For a reference copy of the document with all sections, see nature.com/documents/nr-reporting-summary-flat.pdf

Life sciences study design

All studies must disclose on these points even when the disclosure is negative.

Sample size	To compare production levels across strains and samples, we selected representative cultivation time points of microbial cultures, As we only performed comparisons of relative abundances in microbiological samples, we performed at three biological replicates per experiments (n = 3) and at least two technical measurements (n= 2) of the same sample to ensure reproducibility of our metabolomic data set.
Data exclusions	we have not excluded data
Replication	for metabolomic studies, we performed three biological experiments if possible (n = 3), each of which was measured as one technical replicate measurements. For RNAseq data, the biomass of three biological samples were pooled for one sequencing run to enable sufficient RNA amount and quality. Note: Any statement related to significance were omitted throughout
Randomization	Study focuses on the metabolome and chemical entities of microorganisms but does not include any clinical trial settings nor aims to establish causal inferences within a treatment group. In this study causal relations are drawn from genomic studies and chemical interpretation<br><br>Samples were allocated into groups depending on their origin or specimen type: e.g. feather samples were grouped, as well as UG samples. As feather and UG samples were obtained from the same bird type but from different location, comparison within biosample in relation to their geographic location was possible but not pursued due to the low number of samples. In case of bacterial samples they were grouped according to the specimen to allow further targeted chemical and genomic analysis.

Blinding | Study focuses on the semi-targeted metabolomic analysis and elucidation of chemical entities of microorganisms but does not include any clinical trial settings nor aims to establish causal inferences within a treatment group

Reporting for specific materials, systems and methods

We require information from authors about some types of materials, experimental systems and methods used in many studies. Here, indicate whether each material, system or method listed is relevant to your study. If you are not sure if a list item applies to your research, read the appropriate section before selecting a response.

Materials & experimental systems

n/a	Involved in the study
☒	☐ Antibodies
☒	☐ Eukaryotic cell lines
☒	☐ Palaeontology and archaeology
☐	☒ Animals and other organisms
☒	☐ Clinical data
☒	☐ Dual use research of concern
☒	☐ Plants

Methods

n/a	Involved in the study
☒	☐ ChIP-seq
☒	☐ Flow cytometry
☒	☐ MRI-based neuroimaging

Animals and other research organisms

Policy information about studies involving animals; ARRIVE guidelines recommended for reporting animal research, and Sex and Gender in Research

Laboratory animals: n/a

Wild animals: We collected P. schlegelii individuals from three localities in Papua New Guinea under the research permit 99902341112 and according to the Natural History Museum of Denmark collection guidelines. We report for each individual also the Natural History Museum ID.

Reporting on sex: n/a

Field-collected samples: The research is locally relevant, and we have worked closely with our local partners. The fieldwork protocol, including ethical standards follows Guidelines to the Use of Wild Birds in Research formulated by The Ornithological Council. Samples were collected in 2018 and 2019 from at three locations in Papua New Guinea (PNG) under the research permits no. 99902341112 (to Kasun Bodawatta) and no. 99902260244 (to Knud Jønsson) and exported under export permits no. 019067 in the years 2018 and no. 019362 and no. 019423 in 2019. We , for which we would like to acknowledge support from the New Guinea Binatang Research Centre in Madang and the PNG National Museuam and Art Gallery in Port Moresby for facilitating the fieldwork in Papua New Guinea.

Ethics oversight: The study follows the Ornithological guidelines (best practices for mistnetting and euthanizing birds) as well as general international ethical guidelines for mistnetting birds. Our fieldwork research proposal (along with ethical considerations) for this research was accepted by the National Research Institute in Port Moresby, Papua New Guinea.
Five samples were collected at Yawan, Huon Peninsula, Papua New Guinea (6.10 S, 146.92 E) in 2018 by Kasun Bodawatta (Research permit visa no. 99902341112) and Knud Jønsson (Research permit visa no. 99902260244) and exported under export permit no. 019067. Three samples were collected at Mount Wilhelm, Papua New Guinea (5.76 S 145.18 E) in 2019 by Kasun Bodawatta under Research permit visa no. 99902341112 and exported under export permit no. 019362. And five samples were collected at Mount Scratchley, Papua New Guinea (8.78 S 147.51 E) in 2019 by Knud Jønsson under Research permit visa no. 99902260244 and exported under export permit no. 019423.

Note that full information on the approval of the study protocol must also be provided in the manuscript.

Plants

Seed stocks: n/a

Novel plant genotypes: n/a

Authentication: n/a
